# Capture of an *In Situ* Formed Distanna-S-heterocyclic Carbene

**DOI:** 10.1021/acs.inorgchem.5c00272

**Published:** 2025-04-01

**Authors:** Roman Kimmich, Ralf H. Kern, Markus Strienz, Hartmut Schubert, Claudio Schrenk, Klaus Eichele, Lars Wesemann, Andreas Schnepf

**Affiliations:** Institut für Anorganische Chemie, Auf der Morgenstelle 18, 72076 Tübingen, Germany

## Abstract

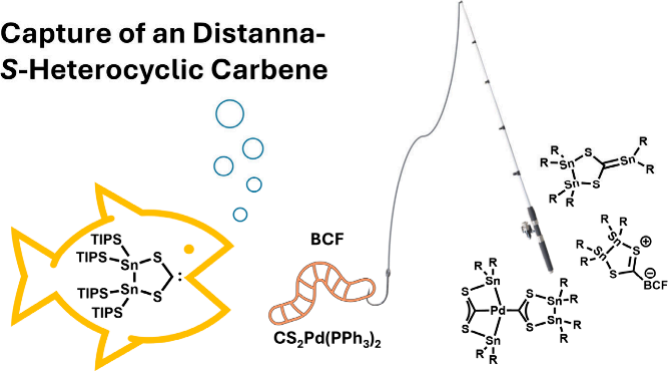

The planar, non-twisted distannene Sn_2_(TIPS)_4_ (**1**, TIPS = Si^*i*^Pr_3_) reacts with CS_2_ to form a tetrathiaethylene derivative via the dimerization of two S-heterocyclic carbenes (SHCs). These intermediary-formed SHCs can be transferred to palladium as ligands or captured with B(C_6_F_5_)_3_ (BCF) and, furthermore, facilitate a new pathway for formation of the stannaethene SHC=Sn(TIPS)_2_ by the reaction of **1** with (PPh_3_)_2_Pd-CS_2_. In addition to the characterization of the new complexes, theoretical calculations of the frontier orbitals were performed, which indicate a high π-acceptor character of the SHC.

## Abstract 2

The reaction of the distannene Sn_2_TIPS_4_ (**1**, TIPS = Si^*i*^Pr_3_) with CS_2_ gives a tetrathiaethylene derivative via the dimerization of an S-heterocyclic carbene (SHC). These intermediary-formed SHCs can be transferred to palladium as ligands or captured with B(C_6_F_5_)_3_ and give access to a new stannaethene.

## Introduction

**Chart 1:**
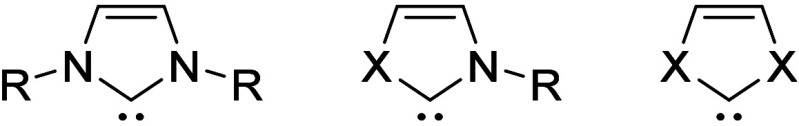
Difference in NHCs, NXHCs, and XXHCs, All Based on the Imidazole Framework with a Saturated or Unsaturated Carbon Backbone (X = S, CR_2_, O)

N-heterocyclic carbenes (NHCs) play a significant role in a variety of fields in inorganic^1^ and organic chemistry.^2,3^ They are widely used in metal complexes and have been extensively investigated and reviewed in numerous articles.^2,4−9^ The majority of NHCs are based on the imidazole framework (Chart 1) in which the carbenoid carbon atom is neighbored by nitrogen atoms, while the backbone can be either saturated or unsaturated. In contrast to triplet carbenes,^10^ NHCs exist in a singlet ground state. This is a consequence of the mesomeric and inductive stabilization of the singlet ground state by the nitrogen atoms. Additionally, kinetic stabilization can be achieved by varying the substituents on the nitrogen atoms.^4−7^

While the usage of NHCs has been extensively discussed in numerous review articles,^1,11−16^ the monosubstituted N,X-heterocyclic carbenes (NXHCs, X = O, S) and their applications in chemistry were investigated rarely. Although there are a few examples of N,O-heterocyclic carbenes and their complexes,^17,18^ the most frequently, yet comparatively little, investigated N,X-heterocyclic carbene is the sulfur structural analogue (NSHC).^19^ These compounds offer the potential for different electronic properties due to the change in the electronic impact of the heteroatom X.^20^ It is frequently observed that NXHCs undergo dimerization, forming a carbon–carbon double bond. An example of a stable NSHC was reported by Arduengo and co-workers who were able to stabilize a NSHC by using the sterically demanding 2,6-di(isopropyl)phenyl (dipp) substituent on the remaining nitrogen atom to achieve kinetic stabilization of the monomeric NSHC.^21^ The majority of known NSHC-metal complexes were synthesized via two principal methods: first, by starting from the alkene and undergoing C–C cleavage, or second, by direct synthesis at a metal center via deprotonation.^19^ The question arises whether it is possible to substitute the second, remaining nitrogen atom in an NSHC by another sulfur atom, resulting in the formation of S,S-heterocyclic carbenes (SHCs). To date there are no examples of “free” S,S-heterocyclic carbenes reported in the literature. In the following, we present the facile synthesis of a SHC formed *in situ*, along with synthetic proof for its existence. Additionally, we present reactions, in which the SHC plays a significant role as ligand and reaction partner.

## Results and Discussion

**Figure 1:**
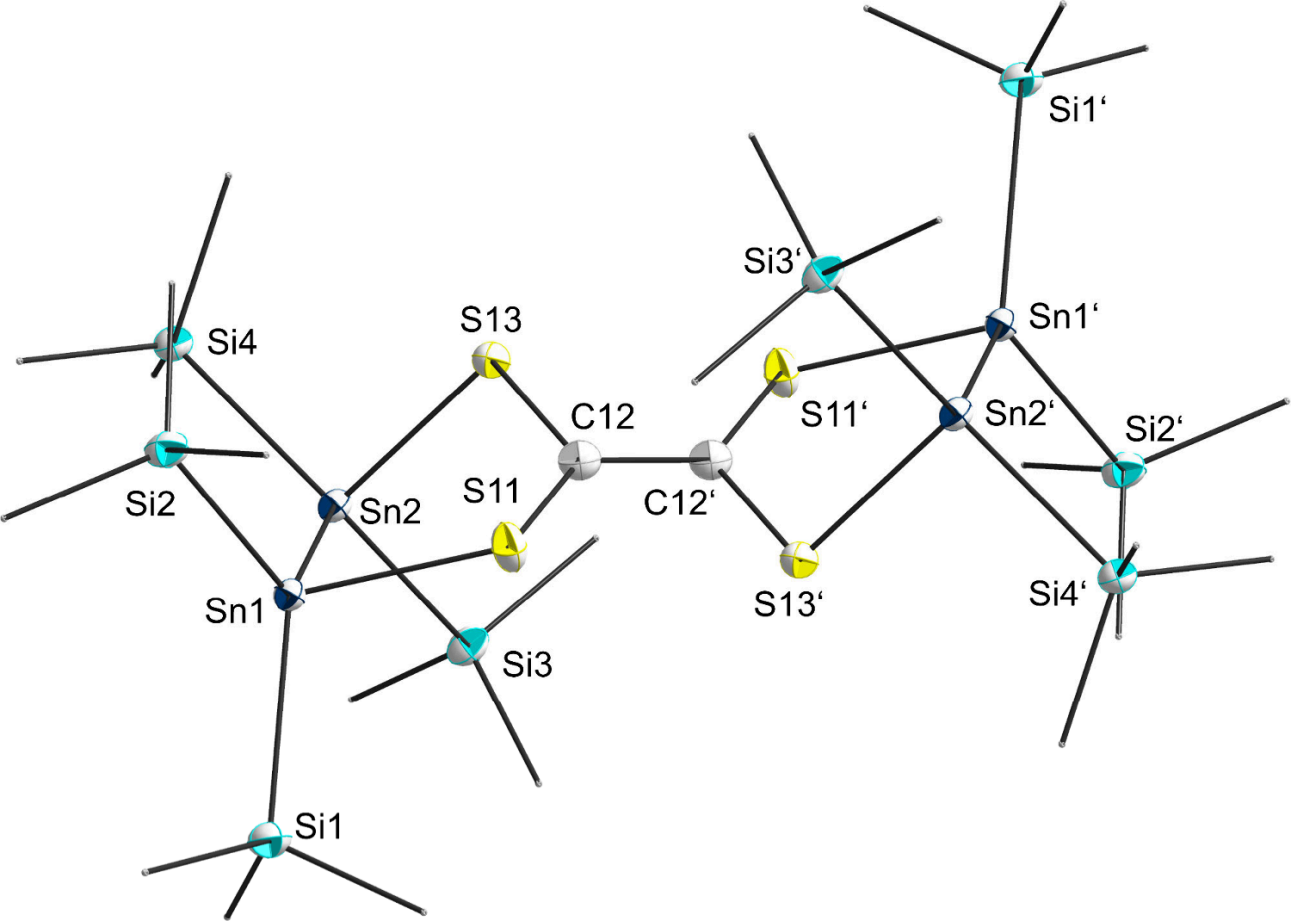
Molecular structure of **2**. Hydrogen atoms and methyl groups of the TIPS substituents are omitted for clarity. Carbon atoms of the TIPS substituents are displayed as a wire model. Silicon (light blue), carbon (gray), sulfur (yellow), and tin (dark blue) atoms are shown as their displacement ellipsoids with 50% probability. Selected bond lengths [pm] and angles [deg]: Sn1–Sn2 284.00(7), Sn1–S11 247.4(2), Sn2–S13 246.3(2), S11–C12 177.3(8), S13–C12 176.2(8), C12–C12′ 138.7(15); S11–Sn1–Sn2 91.84(5), S13–Sn2–Sn1 87.77(5), C12–S11–Sn1 107.2(3), C12–S13–Sn2 102.0(3), S13–C12–S11 122.1(4), C12′–C12–S11 117.9(8), C12′–C12–S13 119.4(8).

During reactivity studies of the previously reported distannene **1**(22) toward cumulenes, only the reaction with CS_2_ gave a clean reaction product. Thereby, distannene **1** was dissolved in *n*-pentane, CS_2_ was added and within a short time, the violet reaction mixture changed its color to yellow. After 3 h of continuous stirring the product precipitated as a yellow powder. Following subsequent concentration of the solution, the product [(TIPS)_4_Sn_2_S_2_C]_2_ (**2**) was obtained in good yields of 81%. By cooling a diluted solution of **2** in heptane at −30 °C for 2 days, yellow single crystals suitable for X-ray diffraction (XRD) were obtained. Tetrathioethylene **2** crystallizes in a triclinic space group *P*1. The crystal structure analysis reveals a newly formed carbon–carbon double bond with a five-membered-ring backbone on each sp^2^-carbon atom (Figure 1). The five-membered ring is complemented by two sulfur and two tin atoms, which are tetra-coordinated with two additional TIPS-substituents at each Sn atom.

**Chart 2:**
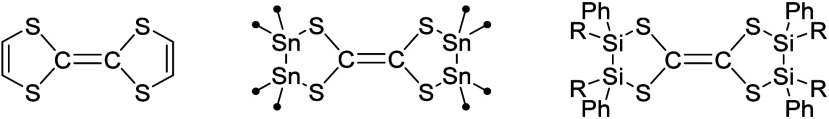
TTF (left) and Structures of **2** (middle, **•** = Si^*i*^Pr_3_) and **3** (right, R = Si^*t*^Bu_3_)

The interatomic distance between C12 and C12′ is 138.7(15) pm and corresponds to a carbon–carbon double bond (calculated for carbon in a double-bond environment: 134 pm^23^). Together with four sulfur atoms the structural motive of a tetrathiaethylene^24^ is realized which can be found in tetrathiafulvalene (TTF) and its derivatives (Chart 2). TTF gained significant recognition through its utilization as a promising organic semiconductor.^25^ In a similar manner, Wiberg and co-workers reacted a disilene with CS_2_, resulting in the formation of an analogous olefine (**3**; Chart 2).^26^ Despite the structural similarities, some differences can be observed in the solid-state structures.

**Figure 2:**
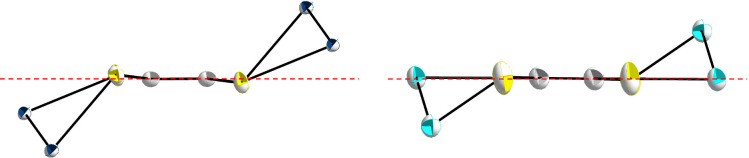
Structural differences in the five-membered rings in **2** (left) and **3** (right). The substituents are omitted for clarity. Silicon (light blue), carbon (gray), sulfur (yellow), and tin (dark blue) atoms are shown as their displacement ellipsoids with 50% probability.

Structural differences between **2** and **3** become clear when looking at both molecules in direction of the C_2_S_4_-plane. In the case of **3**, one of the silicon atoms of the five-membered ring is oriented in the C_2_S_4_ plane while in contrast, both tin atoms of the five-membered ring in **2** are positioned outside the C_2_S_4_ plane (Figure 2). These structural differences could be explained by the different substitution of the silicon atoms in the five-membered ring, opposed to the identical substitution of the tin atoms in **2**. Furthermore, **2** has a *trans*-bent angle of 7.2(1)° and hence must be considered as “quasiplanar”. In contrast, the *trans*-bent angle of **3** is only 1.1(1)°. This result leads to an increased orbital overlap for **3** and consequently a shorter C–C double bond of 133.5(5) pm. This is identical to that observed in a classic C–C double bond. In comparison, **2** has a significantly longer C–C double bond of 138.7(15) pm. This may be a consequence of the reduced orbital overlap resulting from the *trans*-bent angle in the structure of **2**.

The observed C(sp^2^)–S distances are in similar ranges for **2** [176.2(8) and 177.3(8) pm] and **3** [177.1(3) and 176.6(3) pm]. These values are comparable to other examples with a TTF framework.^27,28^ The Sn–Sn distance in **2** is 284.00(7) pm and in good agreement with other Sn–Sn single bonds with a silanide substituent^29^ or in a five-membered ring environment.^30,31^

**Scheme 1:**
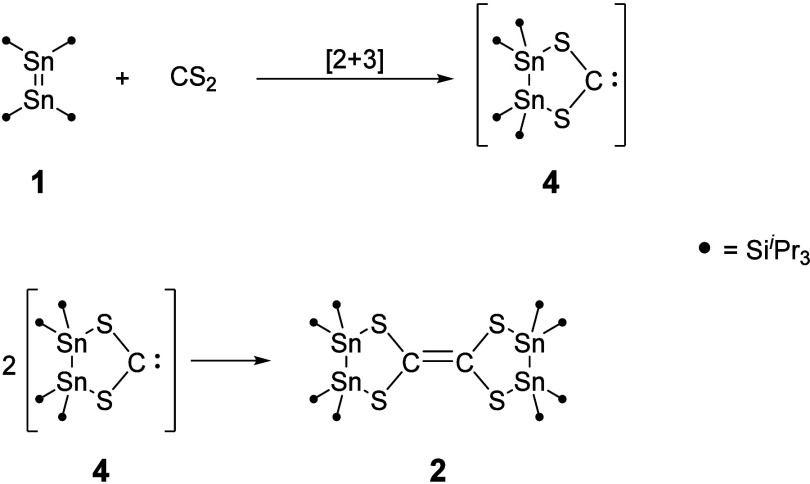
Proposed Mechanism of the Formation of **2** Resulting from Dimerization of the SHC **4**

Wiberg and co-workers explained the formation of **3** by *in situ* formation of an S-heterocyclic carbene and subsequent dimerization. However, they were unable to provide evidence for the existence of a “free” SHC that undergoes dimerization. In the case of **2**, a hypothetical reaction pathway involves a [2 + 3]-cycloaddition reaction of a distannene molecule with CS_2_. The cycloaddition product would be the *in situ* formed S-heterocyclic carbene (TIPS)_4_Sn_2_S_2_C (**4**, SHC) which undergoes dimerization to form the olefine **2** (Scheme 1). This reaction pathway is energetically favored by 427 kJ/mol according to density functional theory (DFT) calculations.

**Chart 3:**
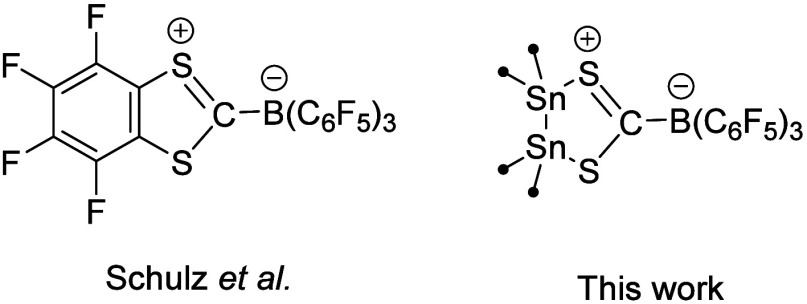
(Left) Capture of C_6_F_4_ by Schulz et al. and (Right) SHC-BCF Adduct **5**

In order to capture the SHC formed *in situ* before it undergoes irreversible dimerization, B(C_6_F_5_)_3_ (BCF) was used to form a Lewis adduct. Such trapping experiments for SHCs have been described in two examples by Nikawa et al. in 2005^32^ and in follow-up work by Kako et al. in 2019.^33^ Both research groups have demonstrated the intermediate formation of SHCs by Lewis-acid–base adducts with fullerenes. In addition, Schulz was able to confirm the formation of a SHC indirectly through adduct formation with BCF.^34^ Schulz and co-workers were able to trap a C_6_F_4_ intermediate with CS_2_ in the presence of BCF under adduct formation (Chart 3).

In our case, to verify the presence of **4**, distannene **1** combined with BCF was suspended in pentane and then CS_2_ was added at room temperature, followed by an immediate color change from dark purple to pale yellow. The solvent was removed *in vacuo* after 10 min of reaction time. To obtain single crystals of the newly formed SHC-BCF adduct (**5**) a concentrated solution of **5** in *o*-difluorobenzene was stored at room temperature overnight. The SHC-BCF adduct **5** can be obtained as yellow crystals in a crystalline yield of 82%. Prior to this experiment, we confirmed that neither distannene **1** nor tetrathioethylene **2** reacts with BCF in the absence of CS_2_ at room temperature in a period of 24 h.

**Figure 3:**
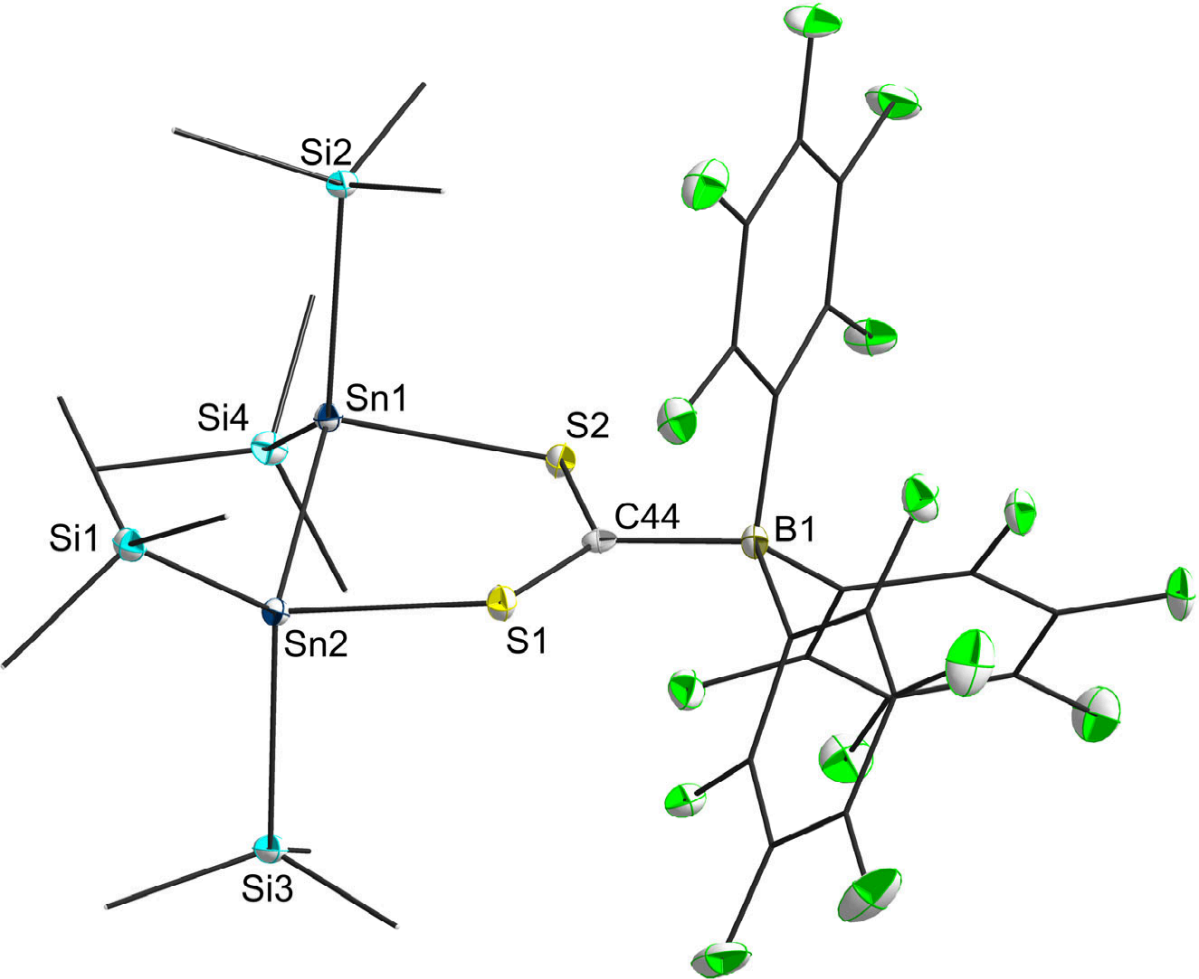
Molecular structure of **5**. Hydrogen atoms and methyl groups of the TIPS substituents are omitted for clarity. Carbon atoms of the TIPS substituents are displayed as a wire model. Silicon (light blue), carbon (gray), fluorine (green), sulfur (yellow), boron (dark green), and tin (dark blue) atoms are shown as their displacement ellipsoids with 50% probability. Selected bond lengths [pm] and angles [deg]: Sn1–Sn2 285.31(2), Sn1–S2 252.52(6), Sn2–S1 254.62(6), S1–C44 168.8(2), S2–C44 169.3(2), C44–B1 169.2(3); S2–Sn1–Sn2 91.776(14), S1–Sn2–Sn1 89.202(14), C44–S1–Sn2 113.08(8), C44–S2–Sn1 111.84(7), B1–C44–S2 114.59(15), S1–C44–B1 117.66(16), S1–C44–S2 127.33(13).

SHC-BCF adduct **5** crystallizes in a monoclinic space group *P*2_1_/*n* (Figure 3). The molecular structure in the solid-state reveals the formation of the Lewis-adduct. Thereby the reaction with BCF is a strong indicator for the intermediate formation of SHC **4**, which subsequently reacts with BCF to **5** before the irreversible dimerization to **2** occurs.

**Scheme 2:**
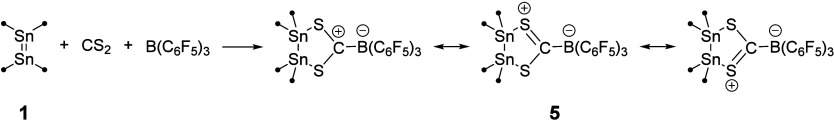
Synthesis of **5** and Resonance Stabilization of the Positive Charge

The coordination environment around C44 is almost trigonal-planar with respect to the S–C–B [114.59(15) and 117.66(16)°] and S–C–S [127.33(13)°] angles, which are nearly 120°. Comparable to the dimer **2**, adduct **5** does not show abnormalities in the Sn–Sn distance [285.31(2) pm]. The C–S distances observed for C44–S1 = 168.8(2) pm and C44–S2 = 169.3(2) pm are significantly shorter than the C–S distances in the dimer **2** [176.2(8) pm], which is a result of the delocalization of the nonbonding lone pairs of the sulfur atoms into the vacant p orbital of the carbene (Scheme 2).

The interatomic distance C44–B1 [169.2(3) pm] in **5** is in good agreement with a known, analogue NHC-BCF adduct from the literature [NHC = 1,3-bis(2,6-di(isopropyl)phenyl)imidazolin-2-ylidene; C_carbene_–B = 169.6(3) pm].^35^

**Figure 4:**
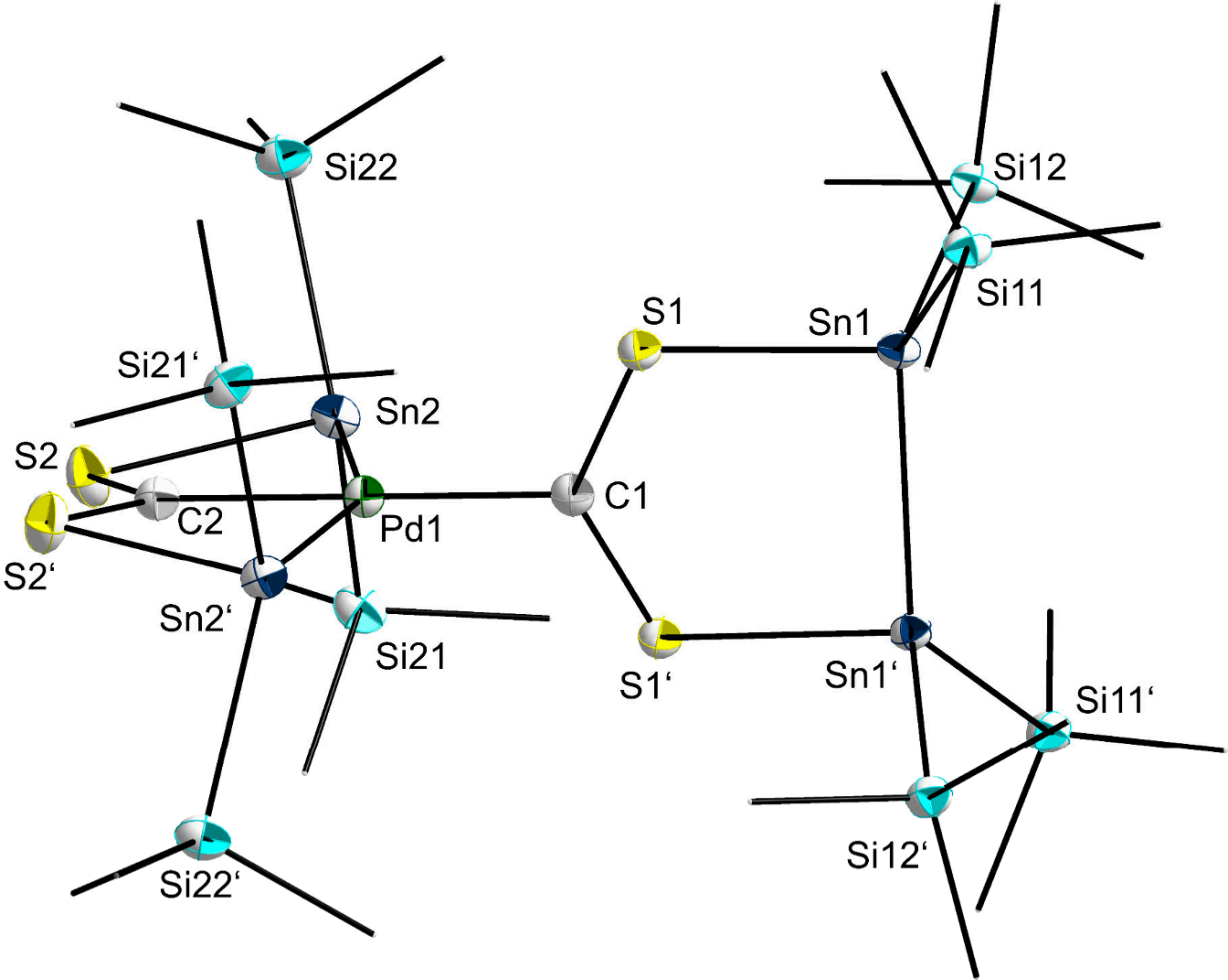
Molecular structure of **7**. Hydrogen atoms and methyl groups of the TIPS substituents are omitted for clarity. Carbon atoms of the TIPS substituents are displayed as a wire model. Silicon (light blue), carbon (gray), palladium (green), sulfur (yellow), and tin (dark blue) atoms are shown as their displacement ellipsoids with 50% probability. Selected bond lengths [pm] and angles [deg]: Sn1–Sn1 281.773(18), Sn1–S1 251.72(4), Sn2–Pd1 264.107(10), Sn2–S2 255.13(4), Pd1–Sn2 264.113(10), Pd1–C1 204.4(2), Pd1–C2 203.2(2), S1–C1 169.85(10), S2–C2 169.90(12); S1–Sn1–Sn1′ 92.026(8), S2–Sn2–Pd1 77.589(10), Sn2–Pd1–Sn2′ 152.247(7), C1–Pd1–Sn2 103.876(4), C2–Pd1–Sn2 76.124(4), C2–Pd1–C1 180.0, C1–S1–Sn1 113.50(6), C2–S2–Sn2 84.26(6), S1–C1–Pd1 116.58(6), S1–C1–S1′ 126.85(13), S2–C2–Pd1 121.18(6), S2–C2–S2′ 117.64(13).

In the following we investigated whether the SHC formed *in situ* can be used as a ligand in transition metal chemistry. In a first attempt, distannene **1** was reacted with CS_2_ in the presence of metal complexes (see Supporting Information, Part 4). Despite numerous attempts, the dimerization of the SHC to **2** is preferred over the substitution of a ligand on a metal atom. In the case of NSHCs, examples can be found in which the dimeric form reacts with metal complexes under C–C bond cleavage.^19^ However, this synthetic approach cannot be applied to **2** for the substrates investigated (see Supporting Information, Part 4). Another possible pathway to form SHC-metal complexes is the reaction of a metal-CS_2_ complex with alkynes by cycloaddition reaction on the metal center. To date, only a few SHC-metal complexes were synthesized by this approach.^36−44^ Following this procedure, we synthesized the literature known (PPh_3_)_2_Pd-CS_2_ complex^45^ (**6**) and reacted it with distannene **1** with the aim to synthesize the SHC-Pd(PPh_3_)_2_ complex. This was achieved by dissolving the precursor (PPh_3_)_2_Pd-CS_2_ in tetrahydrofuran (THF) and reacting it with a solution of **1** in THF. The reaction mixture was stirred for 30 min and stored at room temperature afterward. After a short period of time, dark red, octahedral crystals of **7** were obtained in a crystalline yield of 8%. The crystals were suitable for single-crystal XRD. The newly formed complex crystallizes in a monoclinic space group *I*2/*a* (Figure 4).

The molecular structure in the solid-state reveals successful formation of a Pd-SHC complex **7**. To our surprise, however, the target compound SHC-Pd(PPh_3_)_2_ could not be isolated and PPh_3_ substituents are no longer part of the complex either. Instead, Pd1 is bound to another carbenoid carbon atom C2, which is linked to two stannylene units [Sn(TIPS)_2_] via the sulfur atoms S2 and S2′. The two stannylene units are both bound to the palladium atom Pd1, forming a bicyclic arrangement with the bridging atoms Pd1 and C2. The atoms C1, Pd1, and C2 are located on the 2-fold axis which is why the angle of C1–Pd1–C2 must be stated at 180.0°, while the Sn2–Pd1–Sn2′ angle is 152.247(7)°. This results in the formation of a distorted square planar coordination environment for Pd1. One possible mechanism for the formation of **7** is described in the Supporting Information (Scheme 1S).

The Pd-complex **7** can be described as an SHC and bisstannyl dianion-stabilized Pd(II) center which is in good agreement with the distorted square planar coordination environment in complex **7**. The unusual bicyclic arrangement can be seen as a pincer type ligand. Pd-pincer complexes are well investigated for their catalytic activity in numerous reactions. The used pincer complexes are classified as DCD or DND pincers (Donor–C–Donor, Donor–N–Donor with D = PR_2_, P(OR)_2_, NR_2_, SR, OR, SeR)^46^ while Pd-complex **7** can be seen as the first example of a tin-based SnCSn pincer system, stabilizing a Pd(II) center. Complex **7** is completely insoluble in any organic solvent which prevents the follow-up investigation of its reactivity in solution. When CS_2_ is added to the complex, **7** dissolves under rapid decomposition (Figure S23). Additional analytical data was obtained by energy-dispersive X-ray (EDX) and elemental analysis measurements (Table S1).

**Scheme 3:**
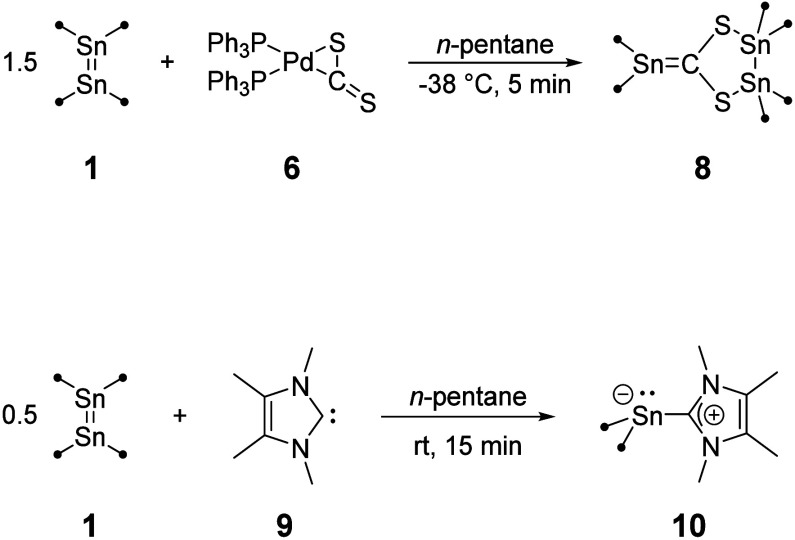
Synthesis of **8** and **10**

**Figure 5:**
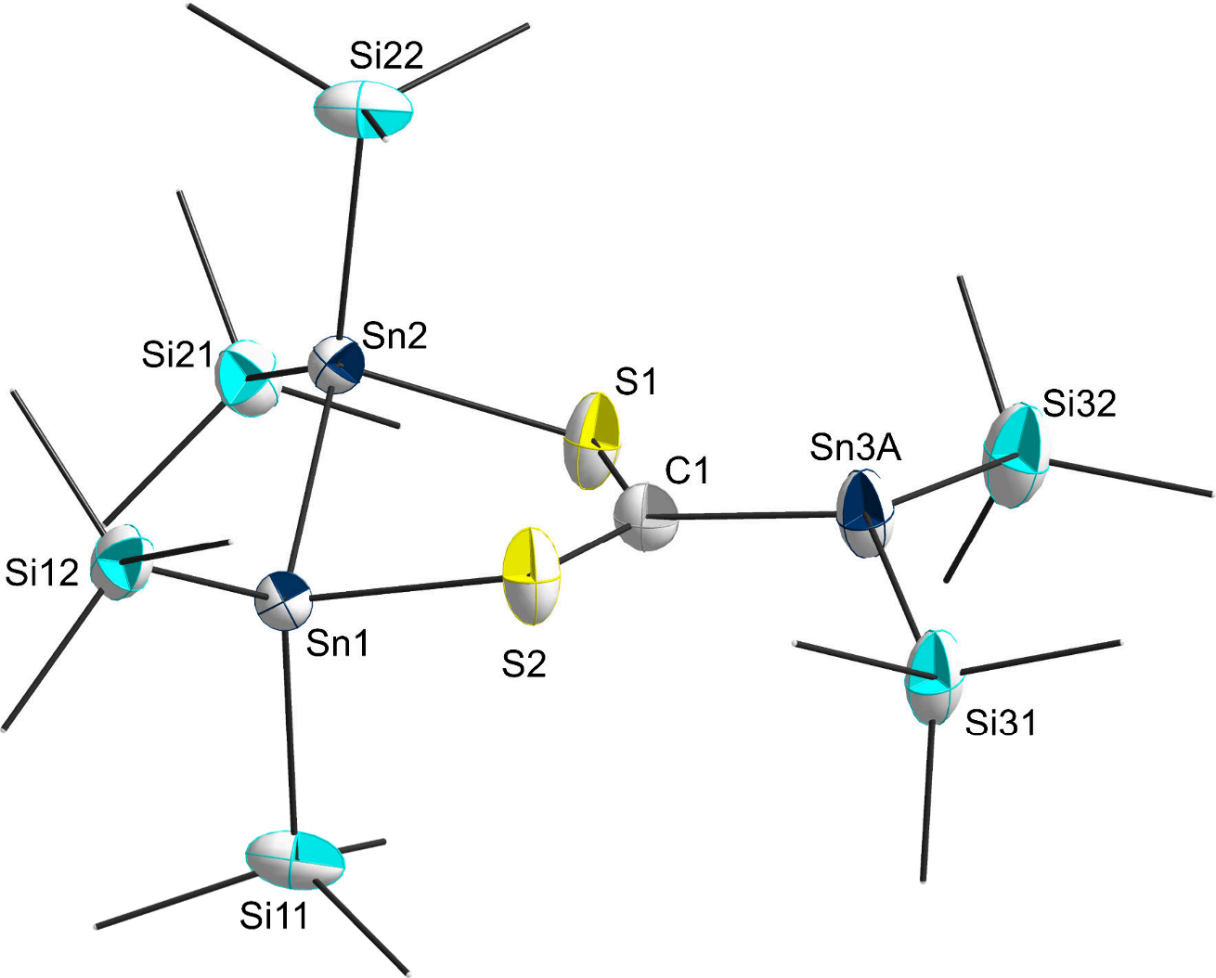
Molecular structure of **8**. Hydrogen atoms and methyl groups of the TIPS substituents are omitted for clarity. Carbon atoms of the TIPS substituents are displayed as a wire model. Silicon (light blue), carbon (gray), sulfur (yellow), and tin (dark blue) atoms are shown as their displacement ellipsoids with 50% probability. Selected bond lengths [pm] and angles [deg]: Sn1–Sn2 281.85(3), Sn1–S2 247.72(9), Sn2–S1 247.84(9), Sn3A–C1 207.3(3), S1–C1 174.1(3), S2–C1 174.2(3); S2–Sn1–Sn2 89.23(2), S1–Sn2–Sn1 89.21(2), C1–S1–Sn2 109.12(11), C1–S2–Sn1 109.13(11).

The low yield of **7** indicates a complex reaction system with numerous unknown side reactions. A hint toward the complicated reaction mixture is the behavior of the reactant (PPh_3_)_2_Pd-CS_2_ in solution. The authors of the (PPh_3_)_2_Pd-CS_2_ complex report that the compound is persistent and soluble in chlorinated solvents but not in diethyl ether.^45^ In contrast, we also observed a solubility of **6** in Et_2_O and THF; however, a rapid color change of the solution from yellow to green was observed. In the case of THF, the solution undergoes a color change to dark brown within a few hours. By allowing a solution of **6** in thf to rest for multiple days, a decomposition product can be isolated (Figure S26). In order to suppress unwanted side reactions with the solvent, a reaction was performed in pentane at −38 °C. To a cold solution of **6** in pentane, **1** dissolved in pentane was added slowly (Scheme 3). The reaction mixture was warmed to room temperature, stirred for 1 h and the dark-violet suspension filtered subsequently. All remaining volatile components were removed *in vacuo* and the remaining solid dissolved in a small amount of pentane. To our surprise, after subsequent concentration of the solution, we could isolate a new compound SHC-Sn(TIPS)_2_ (**8**) in very good yields of 71%. The best preparative results were obtained when using 1.5 equiv of the distannene **1** with respect to (PPh_3_)_2_Pd-CS_2_. The obtained crystals were suitable for single-crystal XRD measurement. **8** crystallizes in a triclinic space group *P*1 (Figure 5).

The molecular structure of **8** in the solid-state shows a compound with a newly formed C–Sn bond. The overall structure can be described as SHC-adduct of Sn(TIPS)_2_. Both subparts carry TIPS substituents, and the structure is indicative for a reaction of an *in situ* formed SHC with an excess of distannene **1** under Sn–Sn bond cleavage. In contrast to **7**, there is no Pd atom present in the newly formed compound and in addition, ^31^P NMR investigations indicate no formation of PPh_3_ but of a palladium phosphine species, whose exact stoichiometric ratio of Pd and phosphine could not be further determined by NMR. The interatomic distance between C1 and Sn3A is 207.3(3) pm. In comparison to published stannaethenes, **8** has a slightly longer double bond.^47−51^ The newly formed double bond can be explained by the π-acceptor abilities of the SHC **4**. The sum of angles around C1 is 359.51°, while the sum of angles around Sn3A is 351.97°. Opposed to the planar coordination environment around C1, the TIPS substituents around Sn3A are (*trans*-bent) angled with an angle out of the S1–C1–Sn3A plane of 31.54°. This is in contrast to the already published stannaethenes, which show either a quasiplanar^48^ or a twisted^47,49−52^ arrangement of the substituents around the Sn–C double bond. Stannaethene **8** exhibits the smallest value for the ^119^Sn NMR chemical shift at δ = 111.7 ppm in comparison to other known stannaethenes (270–835 ppm).^47−51^

To compare **8**, we synthesized another carbene-stannylene complex not with sulfur but with nitrogen substitution in the carbene (NHC) by simple addition of an NHC to the distannene **1** as described for numerous examples.^53−61^ The reaction of 1,3,4,5-tetramethylimidazol-2-ylidene (^4Me^NHC, **9**) with **1** in a 2:1 ratio in pentane yielded a pale-yellow reaction solution (Scheme 3). After workup and crystallization in diethyl ether, the corresponding NHC-stannylene (**10**) complex was isolated in 81% crystalline yield. The crystals obtained were suitable for single-crystal XRD measurements. The stannylene-NHC adduct **10** crystallizes in a triclinic crystal system in the space group *P*1 (Figure S25).

Comparing the solid-state structures of stannaethene **8** and the NHC adduct **10**, major differences are visible. In **10**, the C3–Sn1 bond length is 230.18(11) pm and corresponds to a long C–Sn single bond. In contrast to this, **8** has a C–Sn bond of 207.3(3) pm. While the substituents around the tin atom Sn3A in **8** show a *trans*-bent angle around the double bond, in **10** the tin atom Sn1 exhibits a distorted trigonal pyramidal coordination environment bearing two TIPS and one NHC substituent [Si2–Sn1–C3 = 98.21(3)°, Si1–Sn1–C3 = 98.85(3)°, and Si1–Sn1–Si2 = 111.89(1)°]. This is in agreement with a nonbonding lone pair on Sn1. The experimental results allow the description of **10** as a NHC stabilized stannylene while **8** is a stannaethene derivative. This is further supported by DFT calculations with a Wiberg bond index (WBI) for **10** of 0.58, while the WBI for stannaethene **8** is 1.26. The frontier orbitals of **8** show the π-orbitals expected for a double bond, while for **10** the lone pair on tin does not interact with the vacant p orbital on the carbene (Figures S38 and S39).

**Figure 6:**
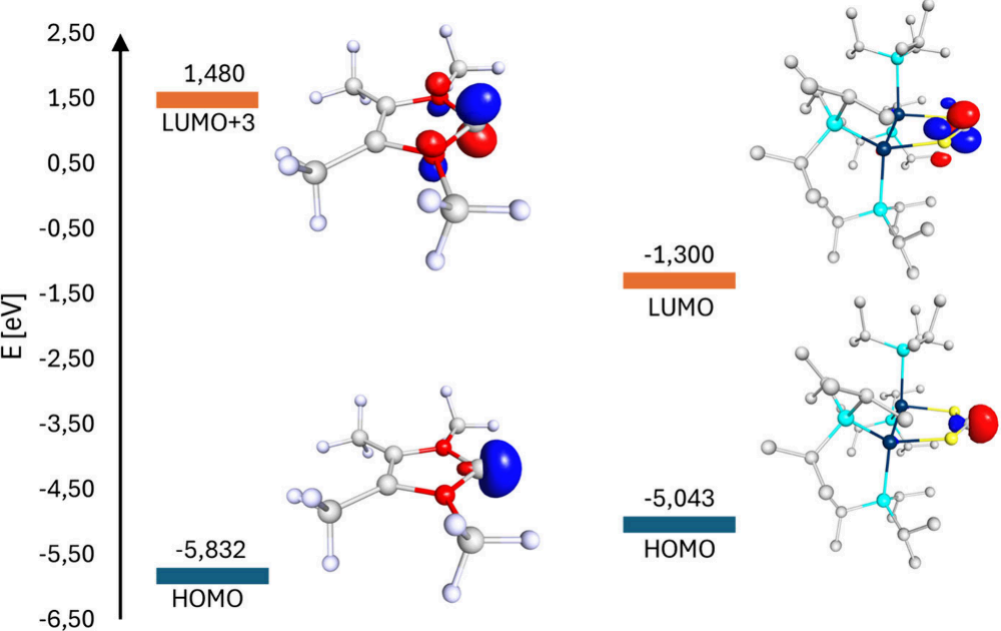
Calculated HOMO, LUMO, and LUMO+3 with their energies of NHC **9** (left) and SHC **4** (right).

The structural differences between **8** and **10** can be attributed to the different electronic properties of the carbenes. In the case of **8**, the π-acceptor ability seems to be enhanced compared to **10** which results in the formation of a Sn–C double bond. In order to gain a better insight into the electronic properties of the carbenes, we calculated the electronic structure for the “free” SHC **4** and the NHC **9** using DFT calculations at the BP86-def2SVP//PBE0-def2TZVP level of theory. We calculated the minimum structure for the “free” SHC **4** in the triplet and singlet ground states. The calculations show an energetically favored singlet ground state by 102.69 kJ/mol. The π-acceptor ability is inversely proportional to the energy level of the corresponding p orbital on the carbenoid carbon atom (Figure 6).

We assigned the molecular orbitals representing the vacant p orbital for both **4** and **9**. In **4**, the vacant p orbital on the carbenoid carbon atom is represented by the LUMO at −1.300 eV. In contrast, the p-like orbital in **9** is found in the LUMO+3 at +1.480 eV. The values for the corresponding HOMO-MOs for both carbenes are in close proximity at −5.832 eV (**9**) and −5.043 eV (**4**). Both carbenes can be described as similar strong σ-donors.

The different bonding situations in **8** and **10** are associated with different optical properties. While **8** has an intense violet color, **10** has a pale-yellow color in solution. This difference is also visible in the corresponding UV/vis spectra for both components (Figures S21 and S22).

Thereby, **8** shows a strong absorption band at 518 nm and a shoulder at 399 nm in solution (Figure S21). Time-dependent DFT (TD-DFT) calculations were conducted to get further insights into the color giving transition. For **8**, the strong absorption band corresponds to the calculated HOMO–LUMO (π–π*-transition) transition at 506.7 nm (Figure S40). This underlines the newly formed double bond and the resulting optical properties.

While the quantum chemical calculations support the results of the experiment the question arises which property of the carbene is responsible for the large energy difference of the p orbital on the carbenoid carbon atom in **4** and the NHC **9**, which ultimately results in structural and optical differences. To get better insight, we distinguished two cases: the influence of the heteroatom in the heterocyclic carbene and the influence of the Group 14 backbone in the five-membered ring.

## Influence of the Heteroatom on the Electronic Situation of the Carbene

**Figure 7:**
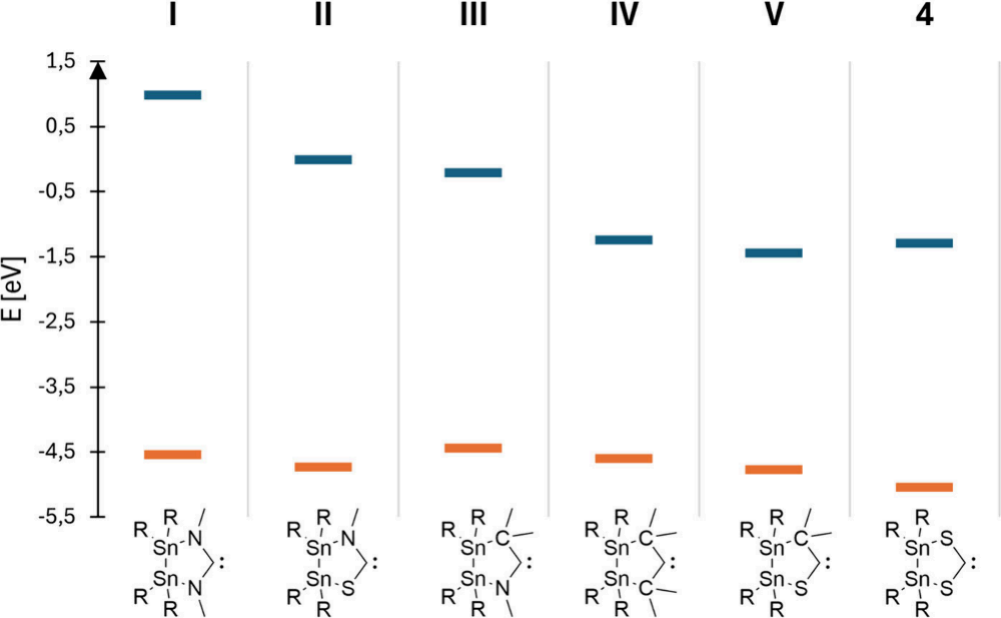
Calculated HOMO/LUMO* energies for the model compounds **I**–**V** and SHC **4**. R = Si^*i*^Pr_3_. *: The LUMO energy refers to the lowest-lying MO representing the carbene-localized p-orbital with the ability to form π-back bonds. **I**: LUMO+9. **II**: LUMO+3. **III**: LUMO+2.

The role of the heteroatom in carbene **4** was investigated in a series of five model compounds **I**–**V** in which one or two sulfur atoms were substituted by NMe or CMe_2_ units (Figure 7). In the presented row, the “classic” NHC **I** exhibits the highest energy for the carbene-localized p orbital (LUMO+9), which is consistent with the low tendency to dimerize for NHCs. The carbenes **II** and **III**, in which one nitrogen atom is substituted, also demonstrate high-lying MOs for the LUMO localized on the carbene atom (**II**, LUMO+3; **III**, LUMO+2). Similar to **I**, **II** and **III** should show weaker π-back-bonding capabilities than the following carbenes. Opposed to carbenes in which a nitrogen atom is present as heteroatom, the carbenes **IV**, **V**, and **4** show comparable low-lying LUMOs and should exhibit better π-acceptor ability than **I**–**III**. The *C*,*S*- or *S*,*S*-carbene **V** and **4** as well as the NSHC **II** have the lowest-lying HOMOs (**II**, −4.732 eV; **V**, −4.773 eV; **4**, −5.043 eV) and represent the weakest σ-donors.

It seems as if the substitution of one nitrogen atom by carbon or sulfur results in a significant lowering of the energy of the carbene-localized p orbital. When both nitrogen atoms are substituted, the corresponding LUMO energy is reduced even further.

## Influence of the Backbone in the Five-Membered Ring on the Electronic Situation of the Carbene

**Figure 8:**
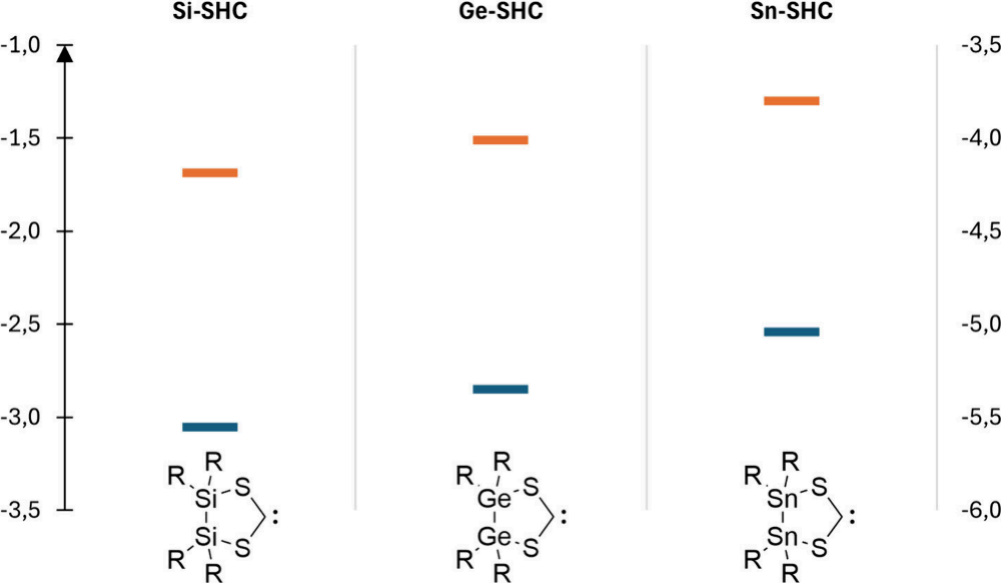
Calculated HOMO/LUMO energies for the model compounds Si-SHC, Ge-SHC, and **4** (Sn-SHC). R = Si^*i*^Pr_3_.

In order to investigate the influence of the Group 14 backbone, the minimum structures of the model compounds Si-SHC and Ge-SHC and Sn-SHC **4** were calculated. A minimum structure for the carbon analogue was not identified, which we attribute to the short C–C bond, resulting in an increased steric repulsion of the TIPS substituents. In contrast to the substitution of the heteroatoms, the HOMO–LUMO gaps for Si-SHC = 3.867 eV, Ge-SHC = 3.841 eV, and Sn-SHC = 3.743 eV exhibit comparable values, while the energies of the corresponding orbitals change (Figure 8).

The HOMO and LUMO energies for the carbene-localized orbitals increase in the case of the heavier homologues. Consequently, a theoretical Si-SHC can be described as a worse σ-donor but a better π-acceptor than a Ge-SHC or a Sn-SHC.

## Conclusion

In conclusion, the reaction of distannene **1** with CS_2_ results in the formation of an S-heterocyclic carbene, which can be identified through the formation of a Lewis acid–base adduct and SHC metal complexes in good yields. Additionally, the formation of the stannaethene structure is indicative of a high π-acceptor character of the SHC, as corroborated by theoretical calculations. Thus, distannene-based SHC acts as a promising ligand in organometallic chemistry, offering the potential to synthesize novel metal complexes due to its ease of synthesis.

## Experimental Section

### General Considerations

All experiments were done in an inert gas atmosphere by using standard Schlenk techniques and gloveboxes. All organic solvents were dried with sodium or calcium hydride and purified via distillation and subsequently degassed by three freeze–pump–thaw cycles. All solids were stored in a glovebox under argon atmosphere. NMR spectroscopic measurements were done on a Bruker AVIIIHD-300, AVII+400, or AVII+500 spectrometer. The chemical shifts are given in ppm against the external standard SiMe_4_. C_6_D_6_ and THF-*d*_8_ were dried with 3 Å molecular sieves. The INEPT and dept45 pulse programs were used for the ^29^Si NMR experiments. For assignment of the proton and carbon signals, detailed analysis of ^1^H, ^13^C{^1^H}, ^1^H–^1^H COSY, ^1^H–^13^C HSQC, ^1^H–^13^C HMBC, and ^13^C{^1^H} DEPT 135 spectra was done. Sn_2_(TIPS)_4_,^22^ (PPh_3_)_2_Pd-CS_2_^45^ and ^4Me^NHC^62^ were synthesized according to literature procedures. BCF and CS_2_ were purchased from Sigma-Aldrich. BCF was used without further purification, and CS_2_ was distilled and degassed before use.

### Solid-State NMR

The ^13^C cross-polarization magic-angle-spinning (CP/MAS) spectrum was obtained on a 300 MHz Bruker Avance III HD NMR spectrometer equipped with a wide-bore magnet, a 4-mm-o.d. MAS probe head, operating at 300.13 MHz (^1^H) and 75.48 MHz (^13^C). The spinning frequency was 8 kHz. Cross-polarization from ^1^H was achieved with nutation frequencies of 90 kHz during a contact time of 1 ms; 44000 scans were acquired after a recycle delay of 2 s. Referencing with respect to Ξ = 25.145020% (^13^C)^63^ was established by adjusting the external magnetic field such that the high-frequency peak in the ^13^C CP/MAS NMR spectrum of an external sample of adamantane matched 38.55 ppm.^64^

### X-ray Crystallography

Crystals were mounted on the diffractometer at 150 or 100 K. Crystals were measured at the following devices:

Device A: Bruker APEX II DUO diffractometer equipped with an IμS microfocus sealed tube and QUAZAR optics for monochromated Mo Kα radiation (λ = 0.71073 Å).

Device B: Bruker Smart APEX II diffractometer with graphite-monochromated Mo Kα radiation (λ = 0.71073 Å),

Device C: Rigaku XtaLAB Synergy-S X-ray diffractometer for single-crystal XRD with an PhotonJet-S series of microfocus X-ray sources [Mo Kα radiation (λ = 0.71073 Å)].

All devices are equipped with an Oxford Cryosystems cryostat. The program packages *CrysAlisPro* (device C), *ApexII* (device B), and *ApexIII* (device A), including *SADABS* for semiempirical absorption correction, were applied. Crystal structure solution and refinement were done with the programs *SHELXS*, *SHELXT*, and *SHELXL*,^65^ implemented in *Olex2*,^66^*WinG9X*,^67^ or *ShelXle*.^68^ The hydrogen atom positions in all compounds were refined using a riding model. For CCDC numbers, see Tables S2 and S3.

### DFT Calculations

All quantum-chemical calculations were performed at a DFT level with the BP86 functional [exchange, LDA + Becke (B88); correlation, LDA (VWN) + Perdew (P86)], using *Turbomole* version 7.4.1 with *Tmolex GUI* version 4.4.1.^69−73^ The def2-SV(P) basis set was used.^74^ The structural optimization was started, if possible, from the obtained molecular structure in solid state. A second structural optimization was done with the PBE0 hybrid functional [3/4(LDA+PBE0)+1/4HF; correlation LDA (PW)+PBE] with Grimmes D3-BJ dispersion and the def2-TZVP basis set.^75−78^ The convergence criterium was set to 10^–6^ H for both optimization steps. Tin atoms were described with the def2-ecp. The minimum structure was confined with vibration analysis for all compounds.

The WBI for **8** and **10** were calculated as implemented in the *Turbomole* package.

TD-DFT calculations were performed for the geometry optimized structure of compound **8** using the Tamm–Dancoff approximation on the PBE0/TZVPP level of theory with the COSMO model to simulate the solvent pentane with an epsilon of 1.356.^79,80^

Further information and results of the quantum-chemical calculations can be found in the Supporting Information.

### Synthesis of [(TIPS)_4_Sn_2_S_2_C]_2_ (**2**)


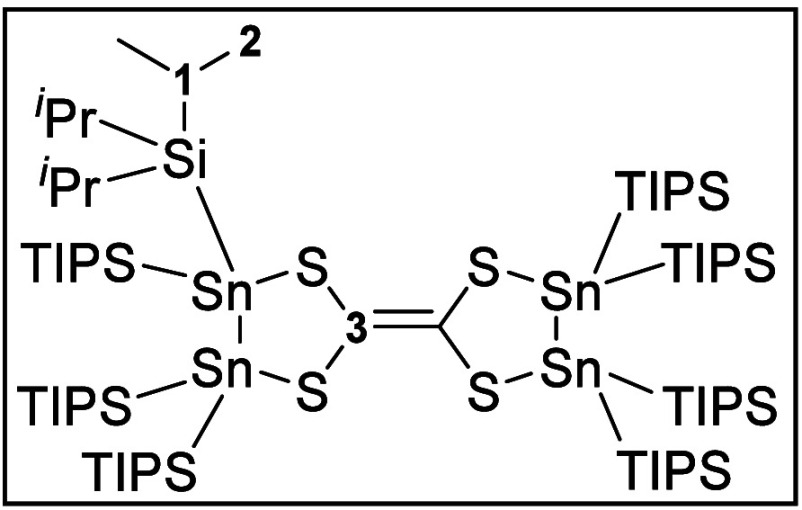
The distannene Sn_2_(TIPS)_4_ (**1**; 50.0 mg, 57.7 μmol, 1.00 equiv) was dissolved in *n*-pentane (5.00 mL) and mixed with CS_2_ (6.98 μL, 115 μmol, 2.00 equiv) at room temperature, whereby a color change from violet to orange red could be seen within 1 min. The reaction mixture was stirred for 3 h, resulting in a yellow solid precipitating out of the solution. To precipitate the product even more, the reaction mixture was cooled to −38 °C overnight. Subsequently the remaining solution was removed, and the yellow powder **2** was dried under reduced pressure (43.9 mg, 23.3 μmol, 81%). By keeping a diluted solution of the product **2** in *n*-heptane at −30 °C, yellow single crystals suitable for XRD were obtained within 2 days. The compound partially decomposes in solution at room temperature within 1–2 days and should be stored dried as a solid at room temperature or in solution at −38 °C.

^1^H NMR (300.13 MHz, C_6_D_12_): δ 1.20–1.37 (m, 144H, H-2), 1.56–1.74 (m, 24H, H-1). ^13^C{^1^H} NMR (75.47 MHz, C_6_D_12_): δ 16.7 (C-1), 20.3 (C-2), 20.8 (C-2), 139.2 (C-3). ^13^C MAS NMR (75.47 MHz): δ 127 (C-3). ^29^Si{^1^H} NMR (59.63 MHz, C_6_D_12_): δ 29.7 (s). ^119^Sn{^1^H} NMR (111.92 MHz, C_6_D_12_): δ −150.3 (s). UV/vis (*n*-pentane, *c* = 0.022 mmol·L^–1^): λ [nm] (ε [L mol^–1^ cm^–1^]): 430 (11800). Elem anal. Calcd (%) for C_74_H_168_S_4_Si_8_Sn_4_: C, 47.13; H, 8.94; S, 6.80. Found: C, 47.41; H, 8.90; S, 6.46.

### Synthesis of SHC-BCF (**5**)


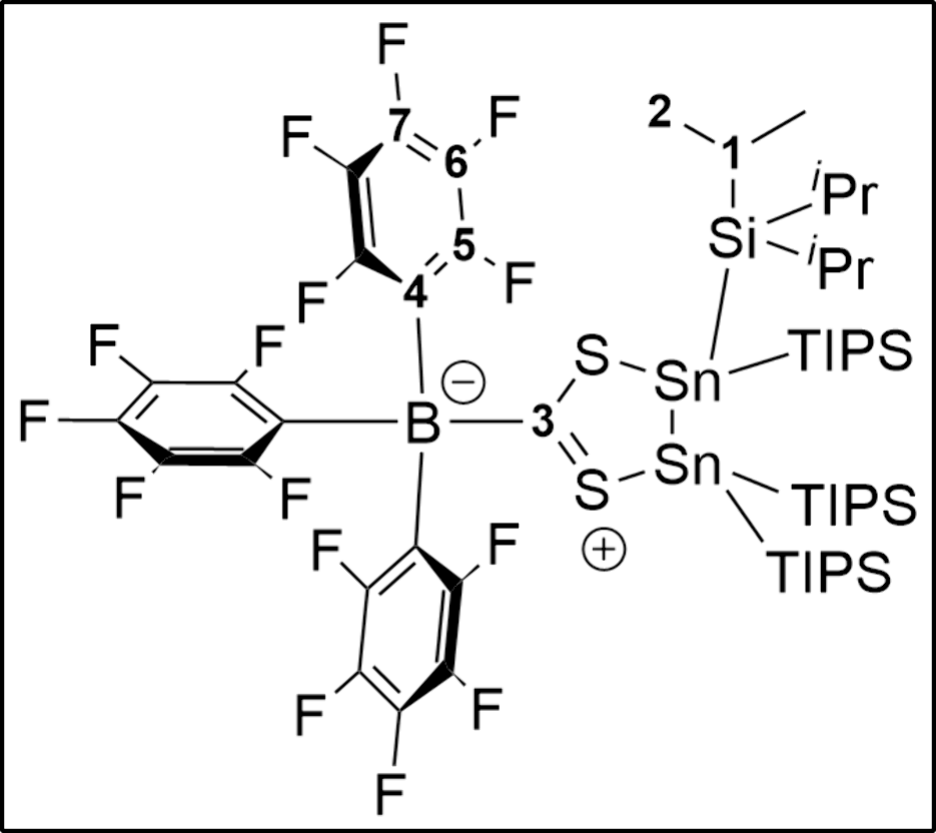
Tris(pentafluorophenyl)borane (47.3 mg, 92.3 μmol, 1.00 equiv) was combined with **1** (80.0 mg, 92.3 μmol, 1.00 equiv) and suspended in *n*-pentane (10.0 mL). CS_2_ (5.58 μL, 92.3 μmol, 1.00 equiv) dissolved in *n*-pentane (2.00 mL) was slowly added to the dark purple suspension, whereby an immediate color change to yellow was observed, as well as the precipitation of a yellow solid within 1 min. The yellow suspension was stirred for 10 min at room temperature followed by the removal of the solvent under reduced pressure. The residue was dissolved in *o*-DFB (3.00 mL), and fine suspended particles were filtered out of the mixture. Product concentration in the filtrate was increased by partial evaporation of the solvent under reduced pressure, and an overnight crystallization at room temperature resulted in yellow crystals of product **5**, which were suitable for X-ray crystallographic examination (111 mg, 82.3 μmol, 82%). The compound partially decomposes in solution at room temperature within 1–2 days and should be stored dried as a solid at room temperature or in solution at −38 °C.

^1^H NMR (700.21 MHz, C_6_D_6_, 298 K): δ 1.09–1.14 (m, 72H, H-2), 1.43 (sept, ^3^*J*_HH_ = 7.4 Hz, H-1). ^13^C{^1^H} NMR (176.08 MHz, C_6_D_6_, 298 K): δ 17.5 (C-1), 20.4 (C-2), 21.0 (C-2), 122.9 (m, very broad, C-3 or C-4), 137.7 (m, *N*_FC_ = 248 Hz, (C-6)), 139.9 (m, *N*_FC_ = 245 Hz, (C-7)), 148.6 (m, *N*_FC_ = 243 Hz, (C-5)). The ^13^C resonance at 122.9 ppm could be assigned to C-3 or C-4, but even various ^13^C{^19^F} experiments could not distinguish between these carbon atoms with complete certainty. Due to the strong broadening, presumably caused by an ^11^B (and possibly also a ^19^F) coupling, one of the two resonances could not be found. ^11^B{^1^H} NMR (128.37 MHz, C_6_D_6_, 299 K): δ −7.7. ^19^F{^1^H} NMR (376.48 MHz, C_6_D_6_, 299 K): δ −165.3 (s, br, F-6), 159.7 (t, ^3^*J*_FF_ = 20.4 Hz, F-7), −125.5 (s, very broad, F-5). ^29^Si NMR (119.23 Hz, C_6_D_6_, 283 K): δ 38.8 (s). ^119^Sn NMR (149.20 MHz, C_6_D_6_, 299 K): δ −19.3 (s). Elem anal. Calcd (%) for C_55_H_84_BF_15_S_2_Si_4_Sn_2_: C, 45.40; H, 5.82; S, 4.41. Found: C, 45.96; H, 5.97; S, 4.33.

### Synthesis of Pd-complex **7**


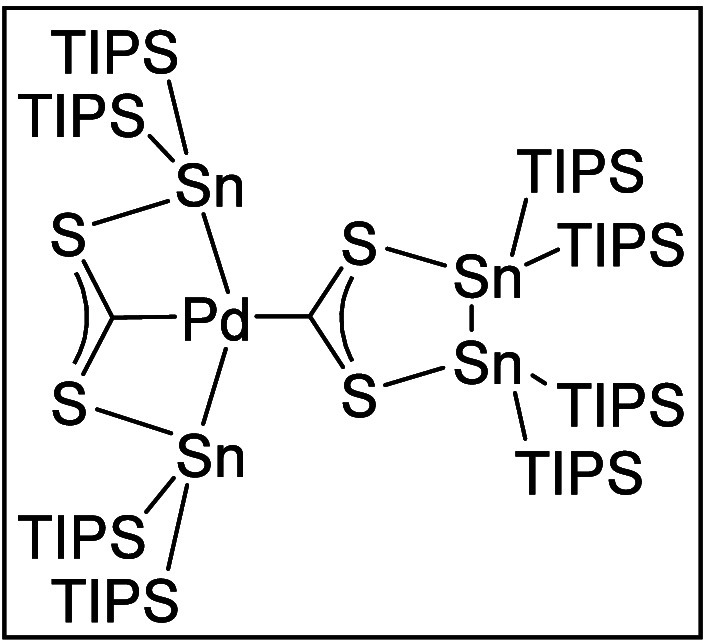
CS_2_Pd(PPh_3_)_2_ (32.6 mg, 46.1 μmol, 1.00 equiv) was mixed with **1** (40.0 mg, 46.1 μmol, 1.00 equiv), and THF (0.15 mL) was added at room temperature without stirring. The resulting dark brown-orange solution was stored for 2 h at room temperature, forming small, octahedral, intensely red to purple-colored crystals. The crystals were suitable for X-ray crystallographic examinations and could be purified simply by washing them with any standard solvent due to their low solubility. In our case, THF (5 mL) was used to wash the crystals in order to obtain product **7** in sufficient purity for elemental analysis (3.80 mg, 2.31 μmol, 8%).

#### NMR Spectroscopy

Due to the poor solubility of the Pd complex in aliphatic, aromatic, halogenated, and etheric solvents as well as acetone and pyridine, it was not possible to carry out NMR measurements in solution. If the purple complex was dissolved in CS_2_, a complete decomposition to a yellow degradation product can be seen within a few minutes. Elem anal. Calcd (%) for C_74_H_168_PdS_4_Si_8_Sn_4_: C, 44.61; H, 8.50; S, 6.44. Found: C, 44.56; H, 8.70; S, 6.63. UV/vis (CS_2_): λ [nm]: 497. (Due to the rapid decomposition of the Pd-complex in CS2, an exact concentration of the dissolved compound cannot be specified, nor can a molar extinction coefficient be determined.)

### Synthesis of Stannaethene **8**


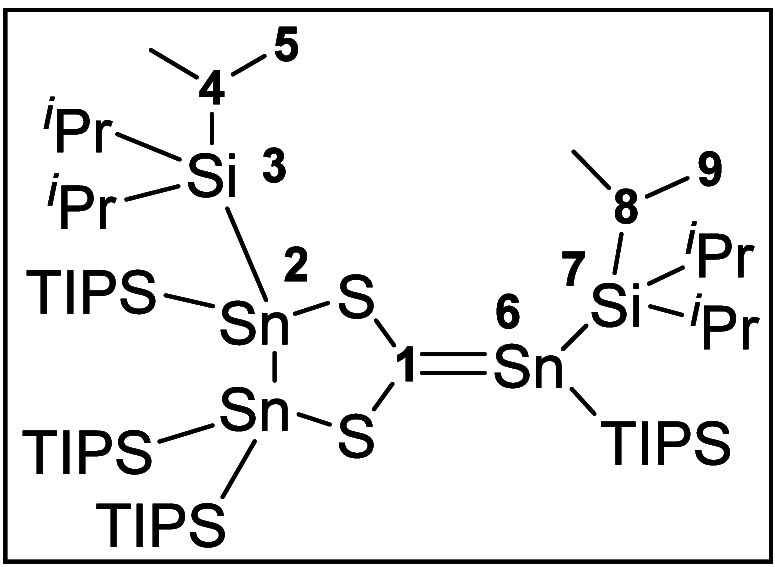
The Pd-complex (PPh_3_)_2_Pd(CS_2_) (27.2 mg, 38.5 μmol, 1.00 equiv) was suspended in *n*-pentane (10.0 mL) and cooled to −38 °C in the same way as a violet solution of distannene **1** (50.0 mg, 57.7 μmol, 1.50 equiv) in *n*-pentane (5.00 mL). At room temperature, the cold solution of the distannene **1** was added dropwise to the cold Pd-suspension over a period of 5 min. After addition, a violet suspension was obtained, which was stirred for 60 min at room temperature before suspended solids were filtered off. Subsequently, the solvent was removed under reduced pressure, and product **8** was extracted from the violet residue with *n*-pentane (3.00 mL), filtered, and dried again under reduced pressure. These steps were repeated until the entire residue dissolved instantaneously in *n*-pentane (3.00 mL). Finally, the concentration of the product was increased by partial evaporation of the solvent under reduced pressure and the product crystallized at room temperature overnight. Compound **8** crystallizes in the form of black-violet crystals, which are suitable for X-ray crystallographic examination (37.3 mg, 27.1 μmol, 71%).

^1^H NMR (600.13 MHz, C_6_D_12_): δ 1.23–1.31 (m, 108H, H-5 + H-9), 1.54 (sept, 12H, ^3^*J*_HH_ = 7.5 Hz, H-4), 1.75 (sept, 6H, ^3^*J*_HH_ = 7.5 Hz, H-8). ^13^C{^1^H} NMR (150.90 MHz, C_6_D_12_): δ 15.6 (C-8), 16.7 (C-4), 20.0 (C-5 or C-9), 20.4 (C-5 or C-9), 20.8 (C-5 or C-9), 200.1 (C-1). ^29^Si{^1^H} NMR (119.23 MHz, C_6_D_12_): δ 31.3 (s, Si-3), 39.7 (s, Si-7). ^119^Sn{^1^H} NMR (223.79 MHz, C_6_D_12_): δ −199.3 (s, Sn-2), 111.7 (s, Sn-6). UV/vis (*n*-pentane, *c* = 0.029 mmol L^–1^): λ [nm] (ε [L mol^–1^ cm^–1^): 518 (15200), 299 (24400). Elem anal. Calcd (%) for C_55_H_126_S_2_Si_6_Sn_3_ + C_5_H_12_ (*n*-pentane, cocrystallized): C, 49.75; H, 9.60; S, 4.43. Found: C, 49.63; H, 9.52; S, 4.44.

### Synthesis of NHC-Sn(TIPS)_2_ (**10**)


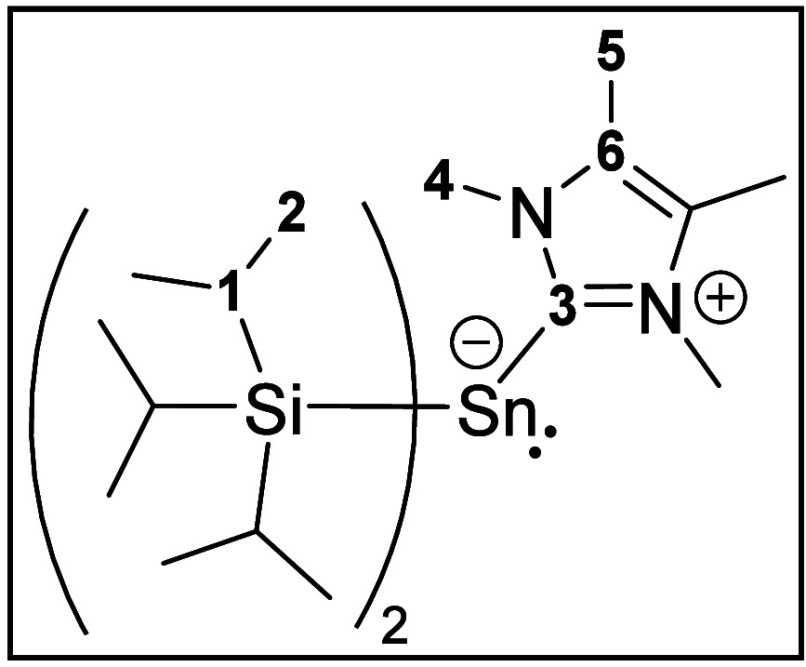
Distannene **1** (40.0 mg, 46.1 μmol, 1.00 equiv) was combined with 1,3,4,5-tetramethylimidazol-2-ylidene (^4Me^NHC) (11.5 mg, 92.3 μmol, 2.00 equiv), and the mixture was dissolved in *n*-pentane (3.00 mL) at room temperature, resulting in an immediate color change from dark purple to yellow. The reaction solution was stirred for 15 min at room temperature, and subsequently the solvent was removed under reduced pressure. The yellow residue was dissolved in Et_2_O (2.00 mL), and the product concentration was increased by partial evaporation of the solvent under reduced pressure. An overnight crystallization at −38 °C resulted in yellow crystals of product **10**, which were suitable for X-ray crystallographic examination (41.9 mg, 75.1 μmol, 81%).

^1^H NMR (400.11 MHz, C_6_D_12_): δ 1.06 (s, br, 18H, H-2), 1.15 (s, br, 18H, H-2), 1.20–1.30, m, 6H, H-1), 2.13 (s, 6H, H-5), 3.99 (s, 6H, H-4). ^13^C{^1^H} NMR (100.61 MHz, C_6_D_12_): δ 8.6 (C-5), 15.9 (C-1), 20.1 (C-2), 20.6 (C-2), 38.4 (C-4), 124.9 (C-6), 171.2 (C-3). ^29^Si{^1^H} NMR (79.49 MHz, C_6_D_12_): δ −110.0 (s). ^119^Sn{^1^H} NMR (149.20 MHz, C_6_D_12_): δ −465.5 (s). UV/vis (*n*-pentane, *c* = 0.072 mmol L^–1^): λ [nm] (ε [L mol^–1^ cm^–1^): 414 (3500), 349 (5600). Elem anal. Calcd (%) for C_25_H_54_N_2_Si_2_Sn: C, 53.85; H, 9.76; N, 5.02. Found: C, 54.03; H, 8.93; N, 5.04.

## Supporting Information Available

Analytical data and spectra, crystallographic data, and DFT calculations (PDF)
