# Supplementary material for: Capture of an In Situ Formed Distanna-S-heterocyclic Carbene
Source: Inorg Chem. 2025 Apr 1;64(14):6793–804. doi: 10.1021/acs.inorgchem.5c00272 (PMC12001250; doi:10.1021/acs.inorgchem.5c00272)
Supplement: Supplementary file 1 — ic5c00272_si_001.pdf [file ic5c00272_si_001.pdf]

Supporting Information

Capture of an *in situ* Formed Distanna-*S*-Heterocyclic Carbene

Roman Kimmich,[‡] Ralf H. Kern,[‡] Markus Strienz, Hartmut Schubert, Claudio Schrenk, Klaus Eichele, Lars Wesemann and Andreas Schnepf.*

Institut für Anorganische Chemie, Auf der Morgenstelle 18, 72076 Tübingen.

*andreas.schnepf@uni-tuebingen.de

Table of contents

1 Spectroscopic data 2

2 UV/Vis data 12

3 EDX-Measurements 14

4 Attempts to capture an SHC, as well as C-C bond cleavage in tetrathiaethylene 2 with metal complexes 15

5 Possible mechanism for the formation of 7 15

6 Crystallographic data 16

6.1 Crystal structure of **10** 16

6.2 Crystal structure of a Pd_6 complex 17

6.3 Crystallographic data for compounds **2**, **5**, **7**, **8** and **10** and the Pd_6 complex. 18

7 Theoretical Calculations 20

7.1 Geometry optimized structures 20

7.2 Selected frontier orbitals of **10** 35

7.3 Selected frontier orbitals of **8** 35

7.4 Calculated UV/Vis spectrum of **8** 36

8 References 37

1 Spectroscopic data

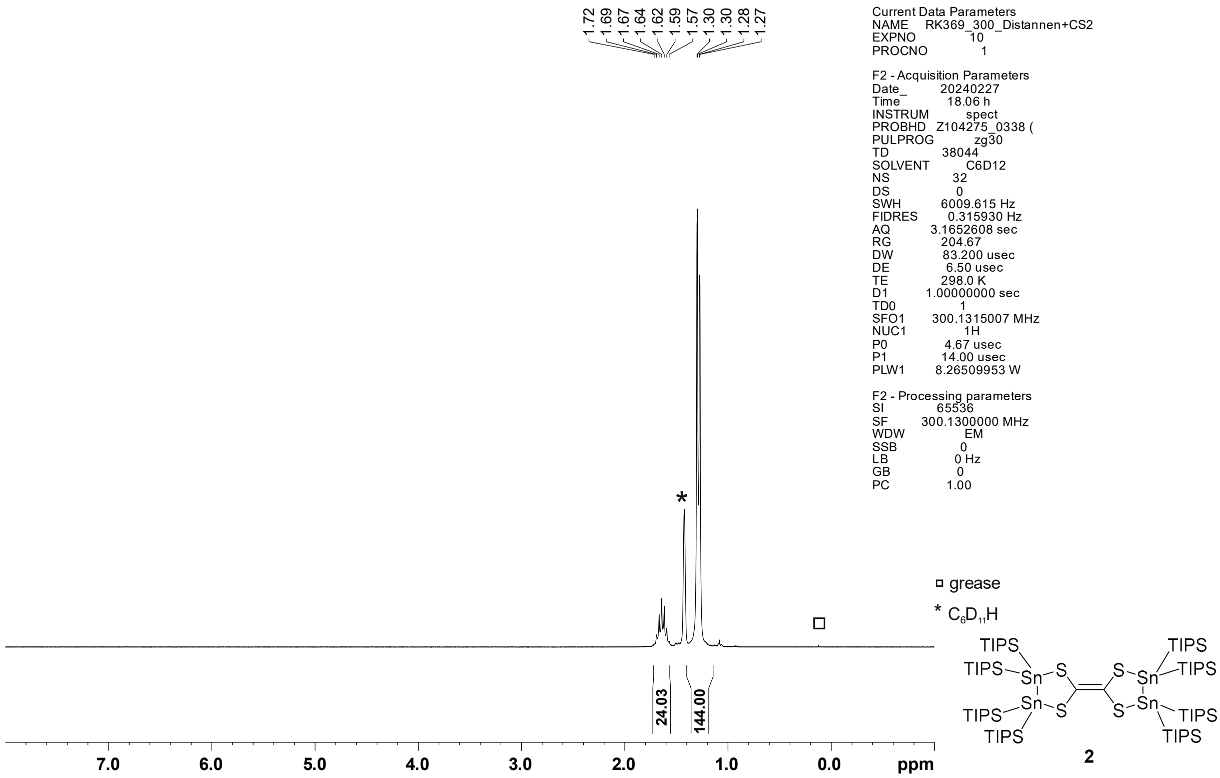

Figure S1: ^{1}H-NMR (300.13 MHz, C_6D_{12}) of **2**.

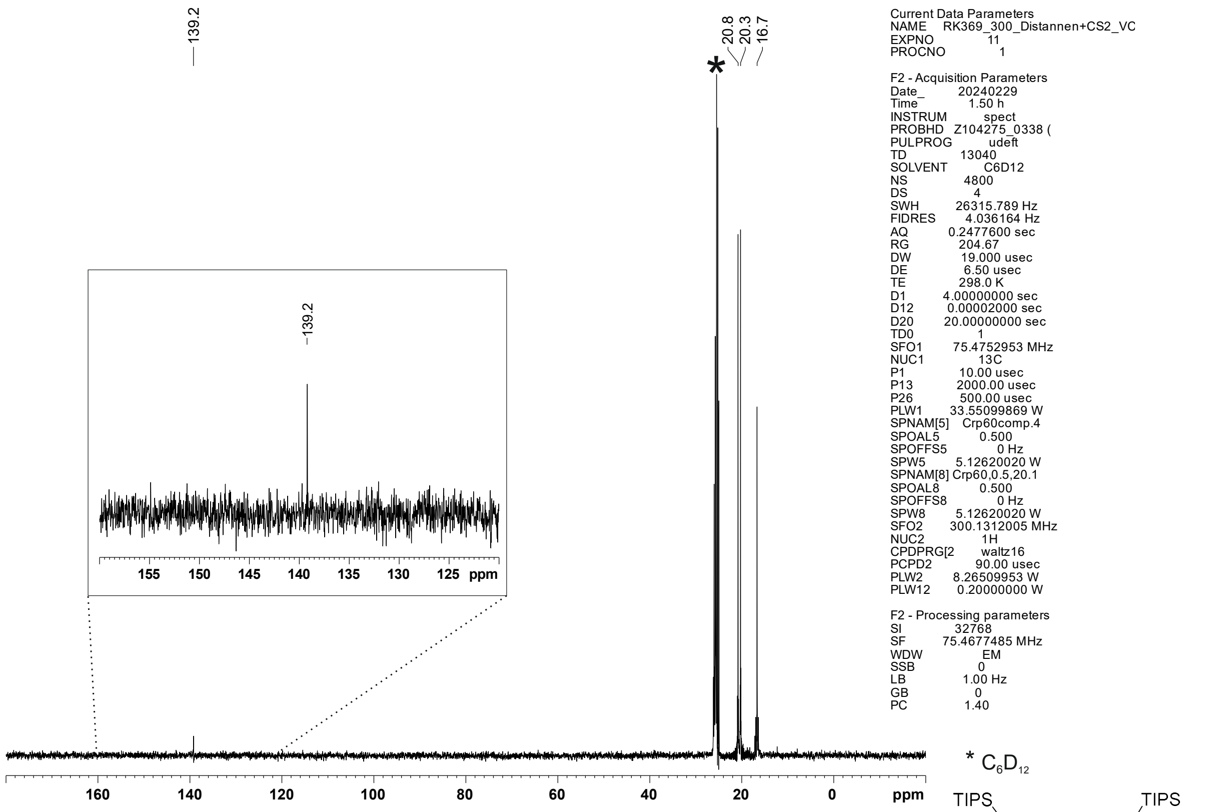

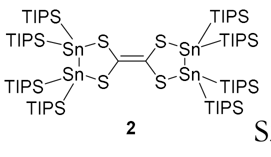

Figure S2: ^{13}C{^{1}H}-UDEFT-NMR (75.48 MHz, C_6D_{12}) of **2**.

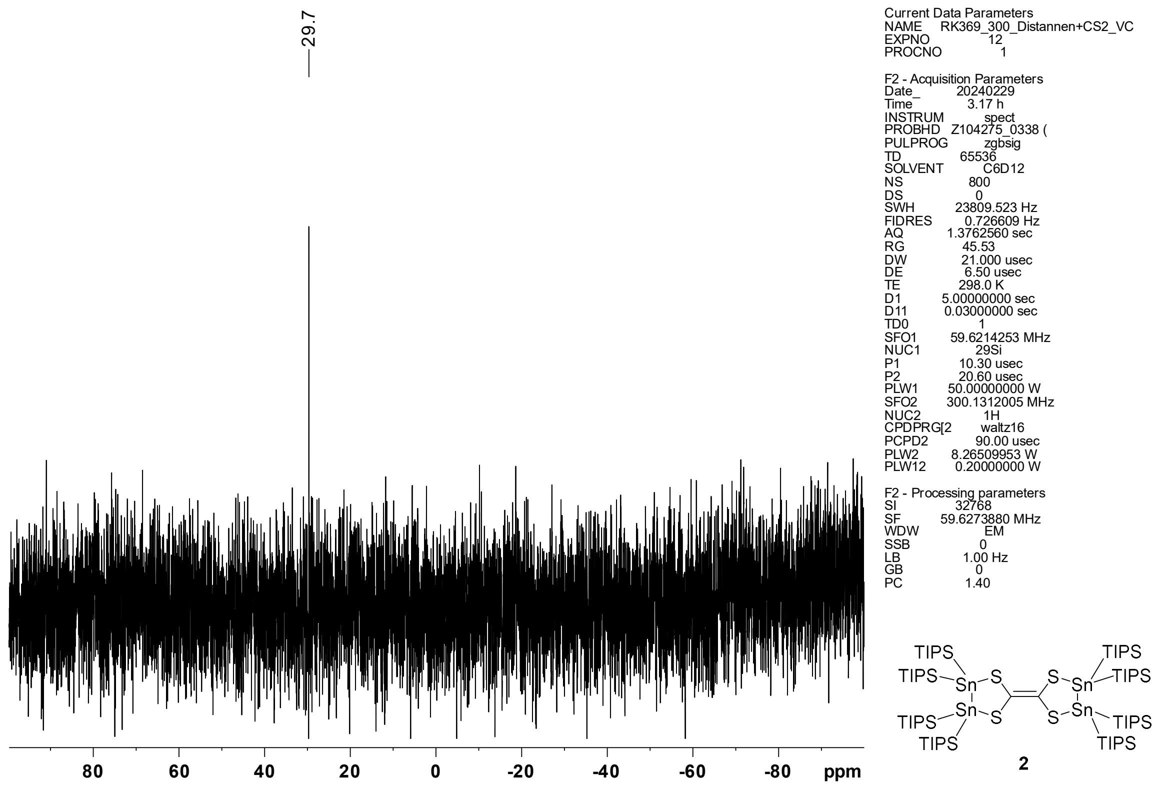

Figure S3: $^{29}Si\{^{1}H\}$-NMR (59.62 MHz, C_6D_{12}) of **2**.

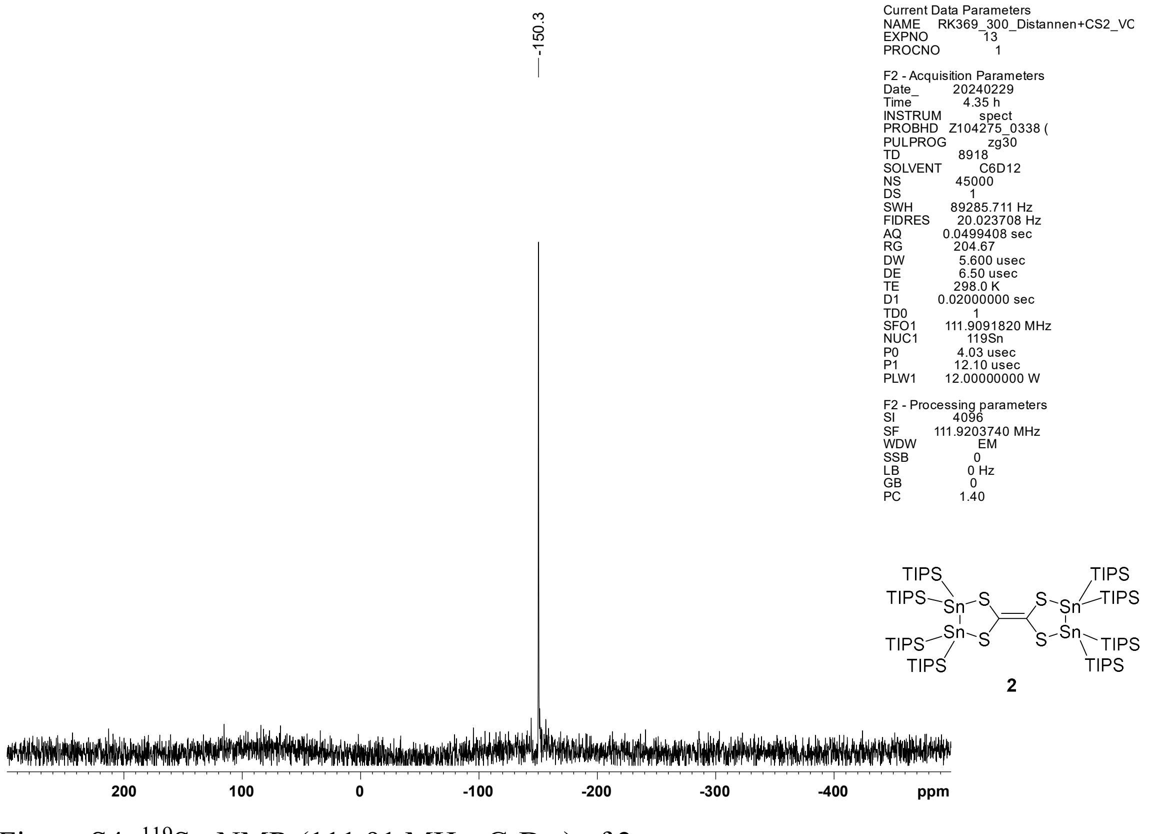

Figure S4: ^{119}Sn-NMR (111.91 MHz, C_6D_{12}) of **2**.

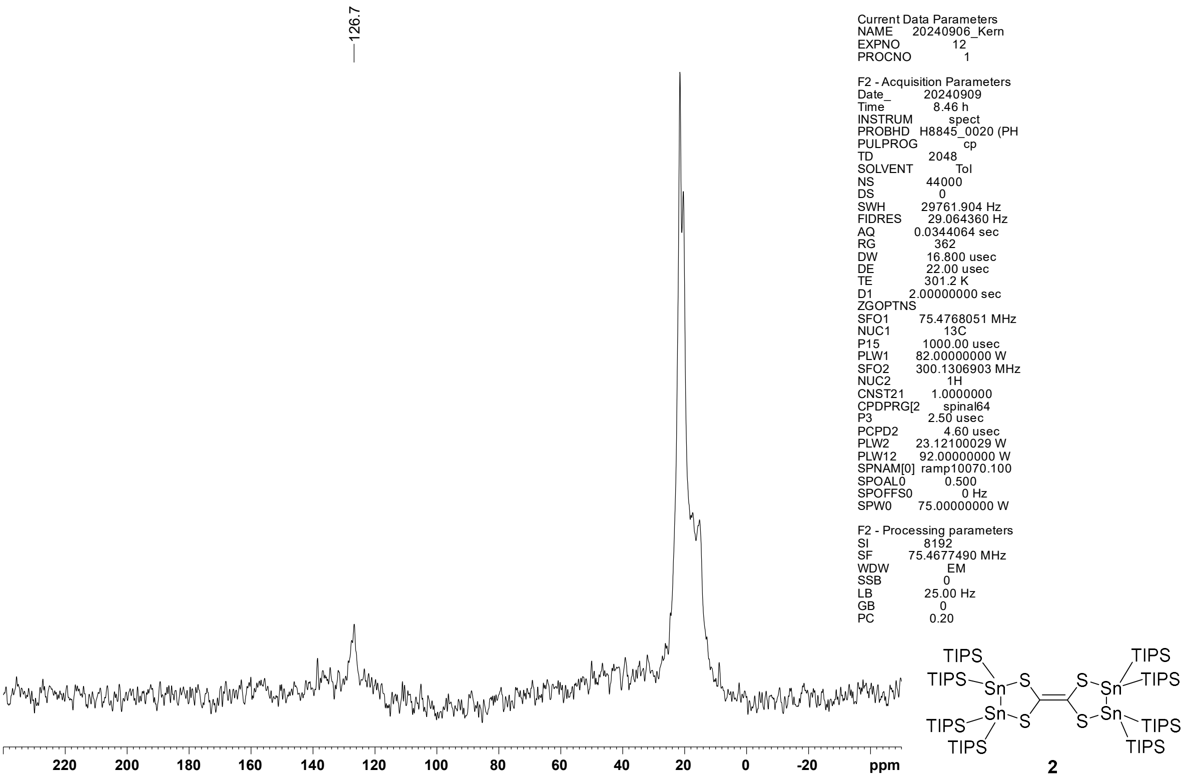

Figure S5: ^{13}C MAS-NMR of **2**.

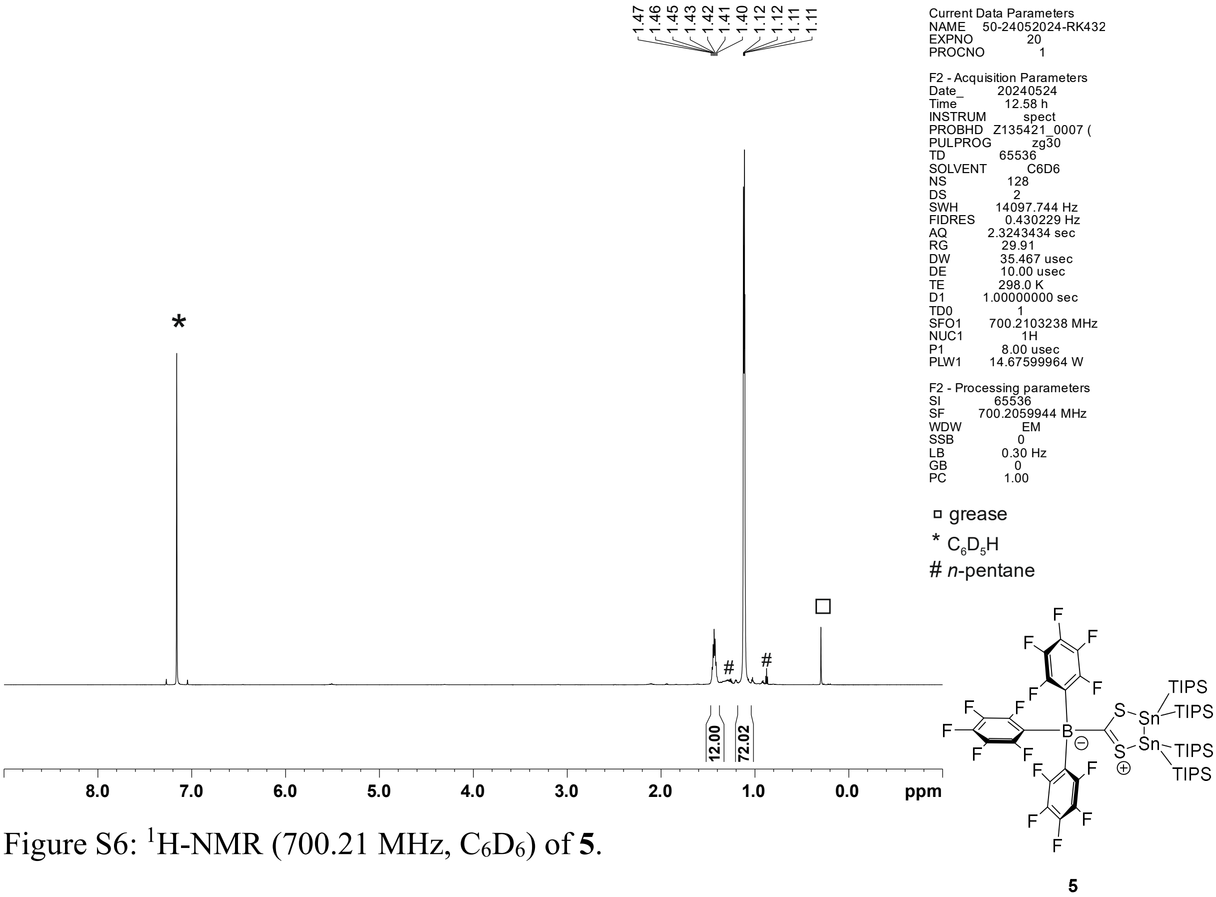

Figure S6: ^{1}H-NMR (700.21 MHz, C_6D_6) of **5**.

5

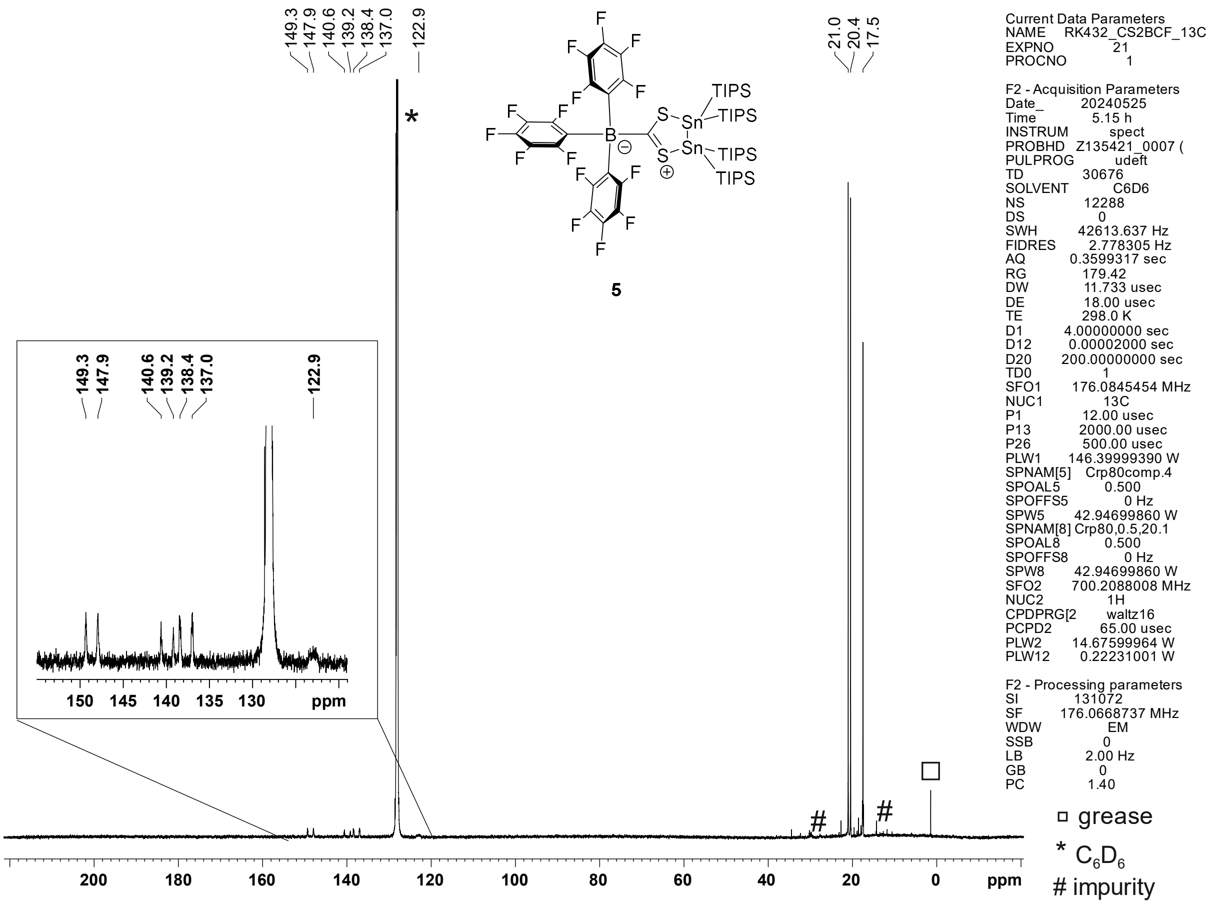

Figure S7: $^{13}C\{^1H\}$-UDEFT-NMR (176.08 MHz, C_6D_6) of **5.** The multiplets in $^{13}C\{^1H\}$-NMR spectra of pentafluorophenyl groups arise from isotopomers of ^{13}C-AA'BB'C spin systems.[1] Determination of coupling constants in higher-order multiplets requires detailed analysis, provided a sufficient number of transitions is observable. In many cases, only the separation N between the constituents of the principal doublet, representing 50% of the intensity of the multiplet, can be extracted.[2] This splitting corresponds to the sum of the $^1J_{A,C\text{-}13}$ and $^nJ_{A',C\text{-}13}$ coupling constants.

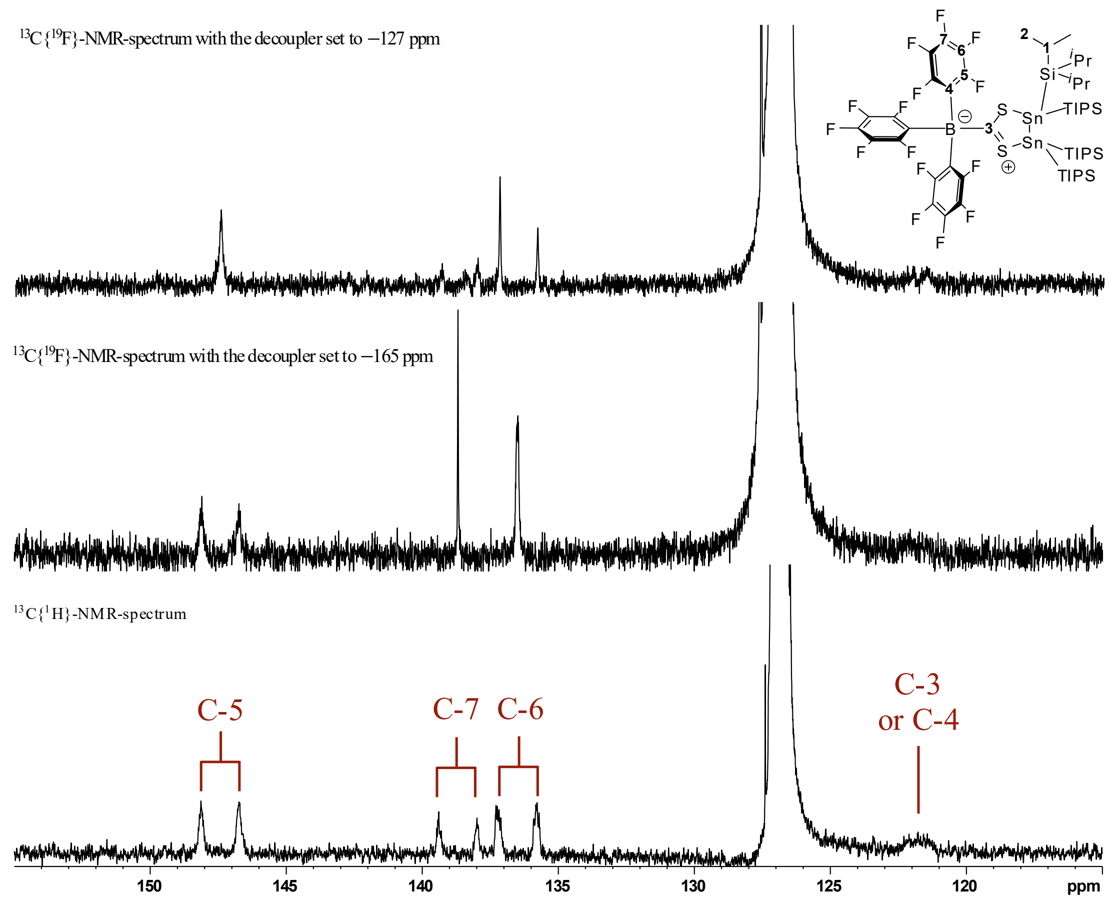

Figure S8: ^{13}C-UDEFT-NMR (176.08 MHz, C_6D_6) of **5** with different decoupler settings.

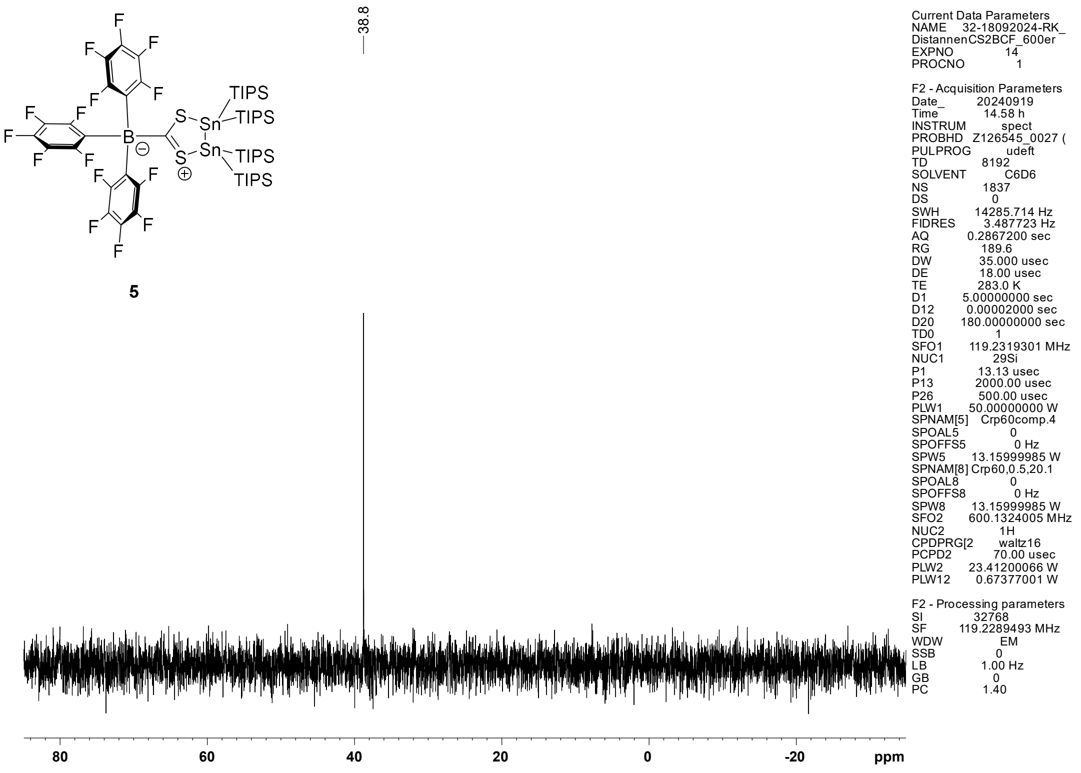

Figure S9: $^{29}Si\{^{1}H\}$-UDEFT-NMR (119.23 MHz, C_6D_6) of **5**.

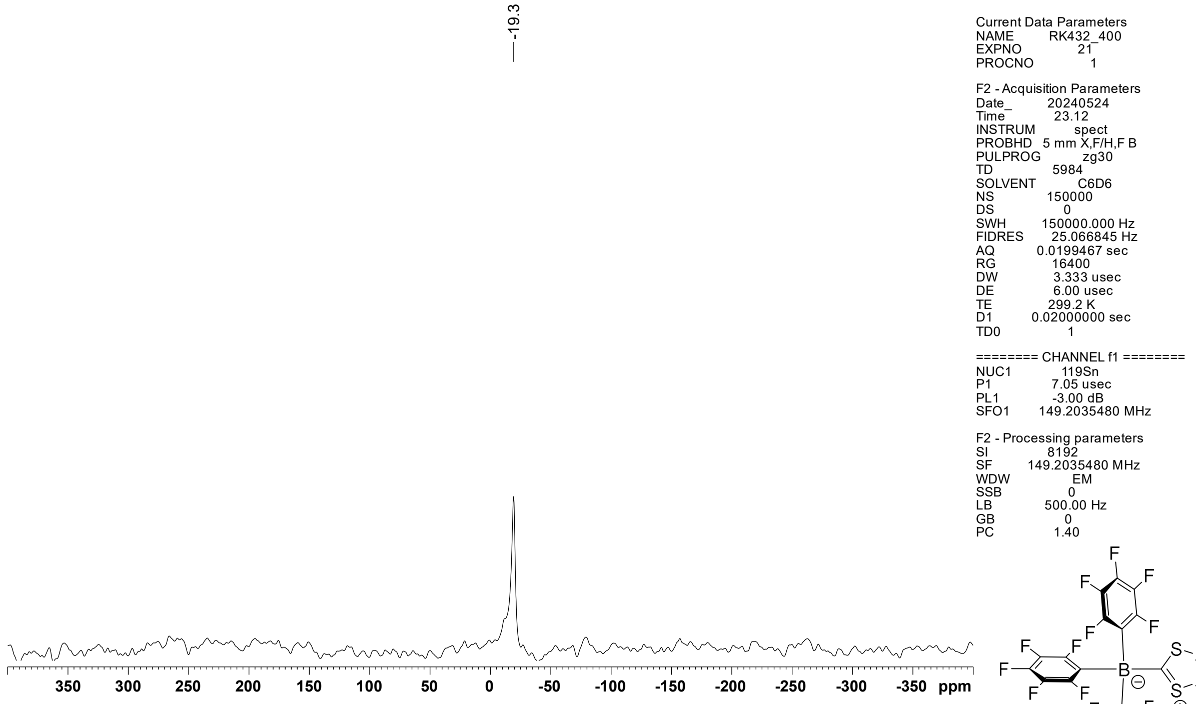

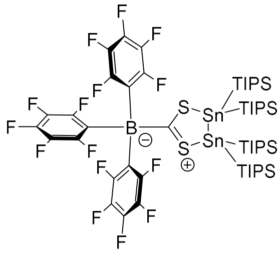

Figure S10: ^{119}Sn-NMR (149.20 MHz, C_6D_6) of **5**.

5

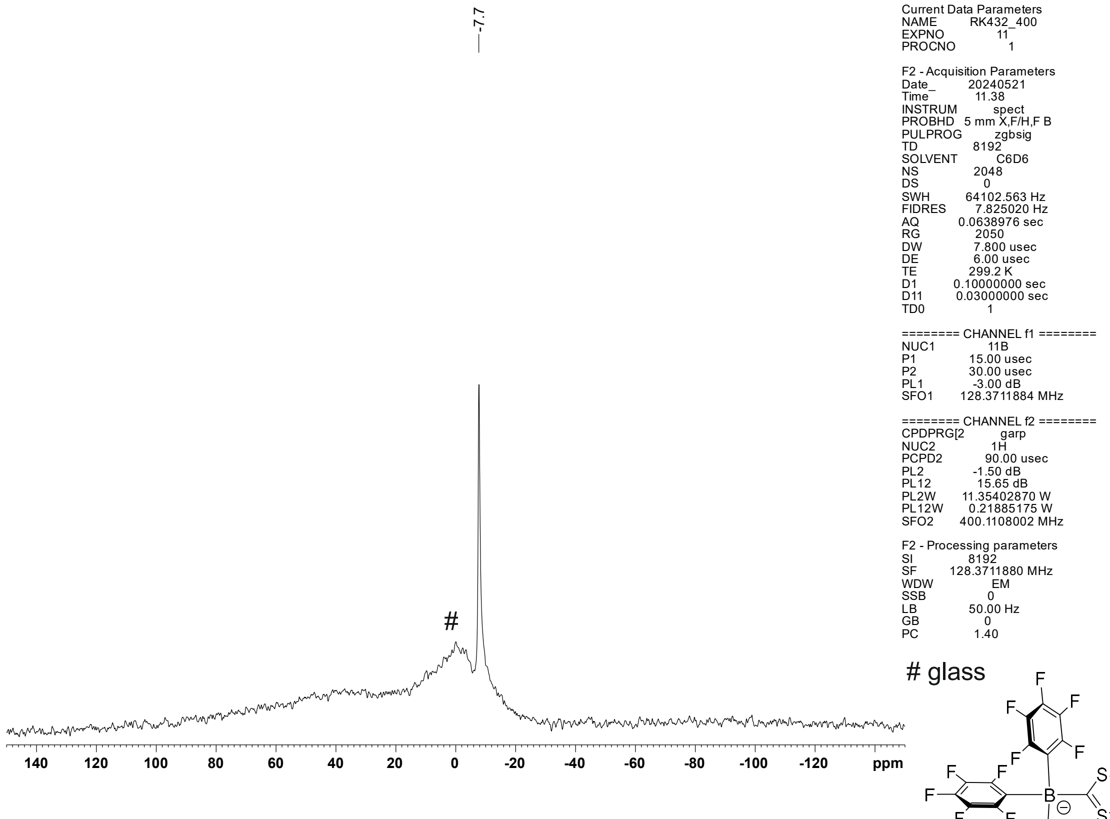

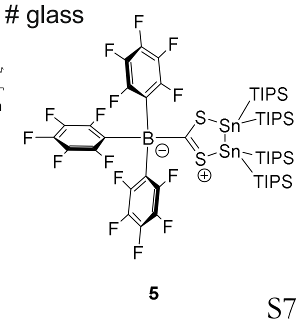

Figure S11: ^{11}B{^{1}H}-NMR (128.37 MHz, C_6D_6) of **5**.

5

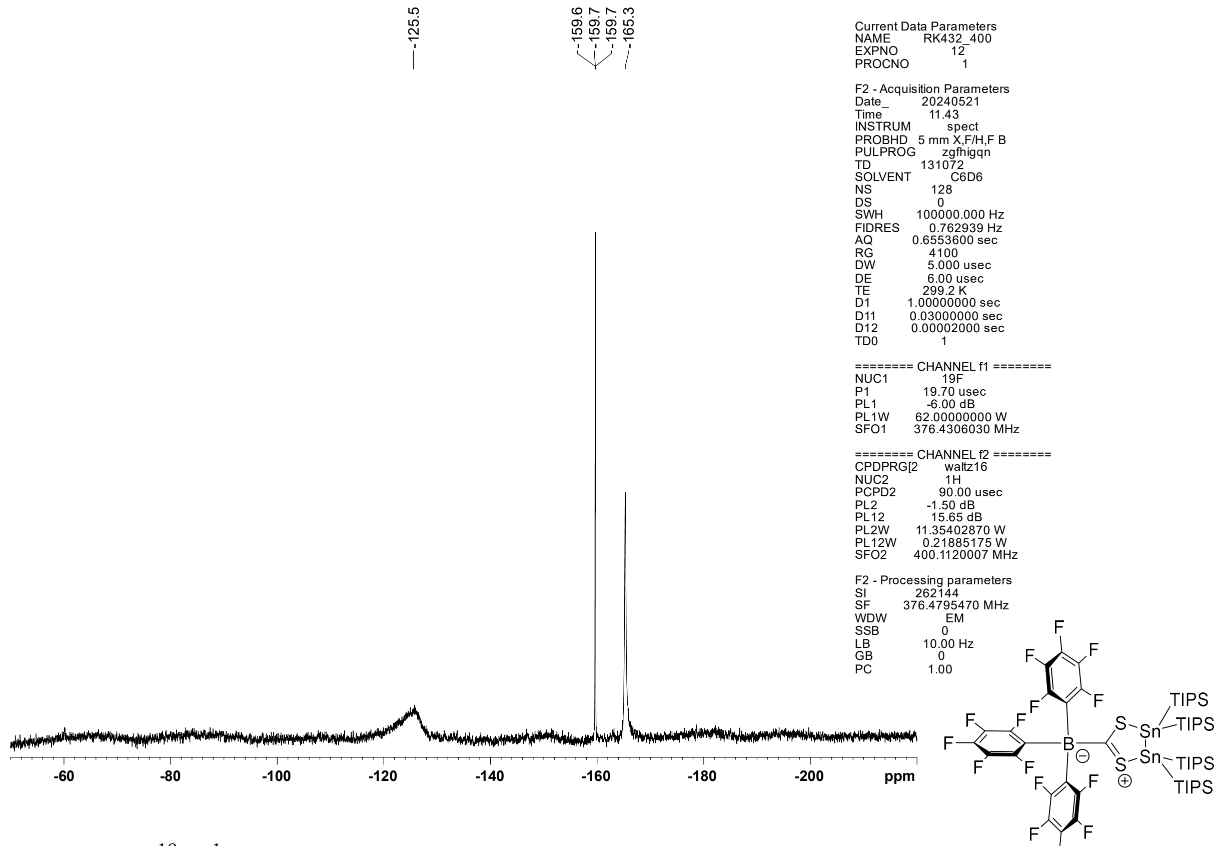

Figure S12: $^{19}F\{^{1}H\}$-NMR (376.40 MHz, C_6D_6) of **5**.

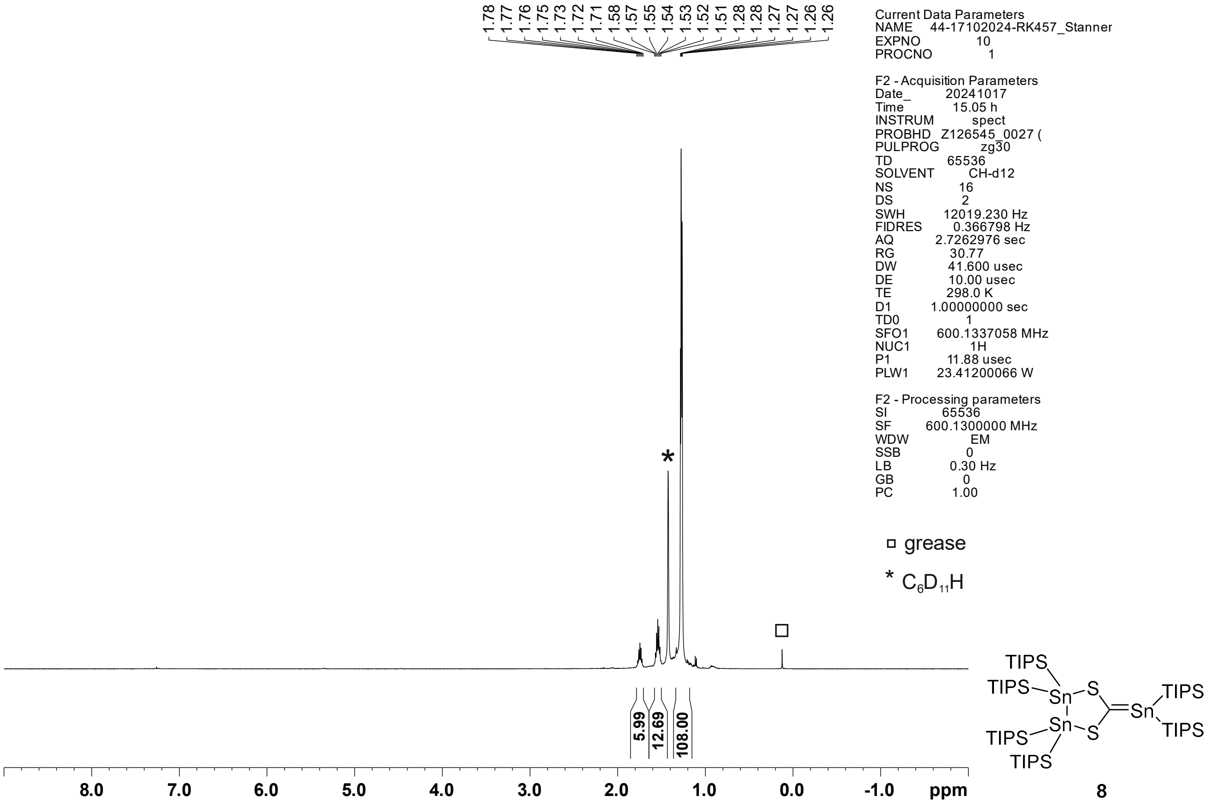

Figure S13: ^{1}H-NMR (600.13 MHz, C_6D_{12}) of **8**.

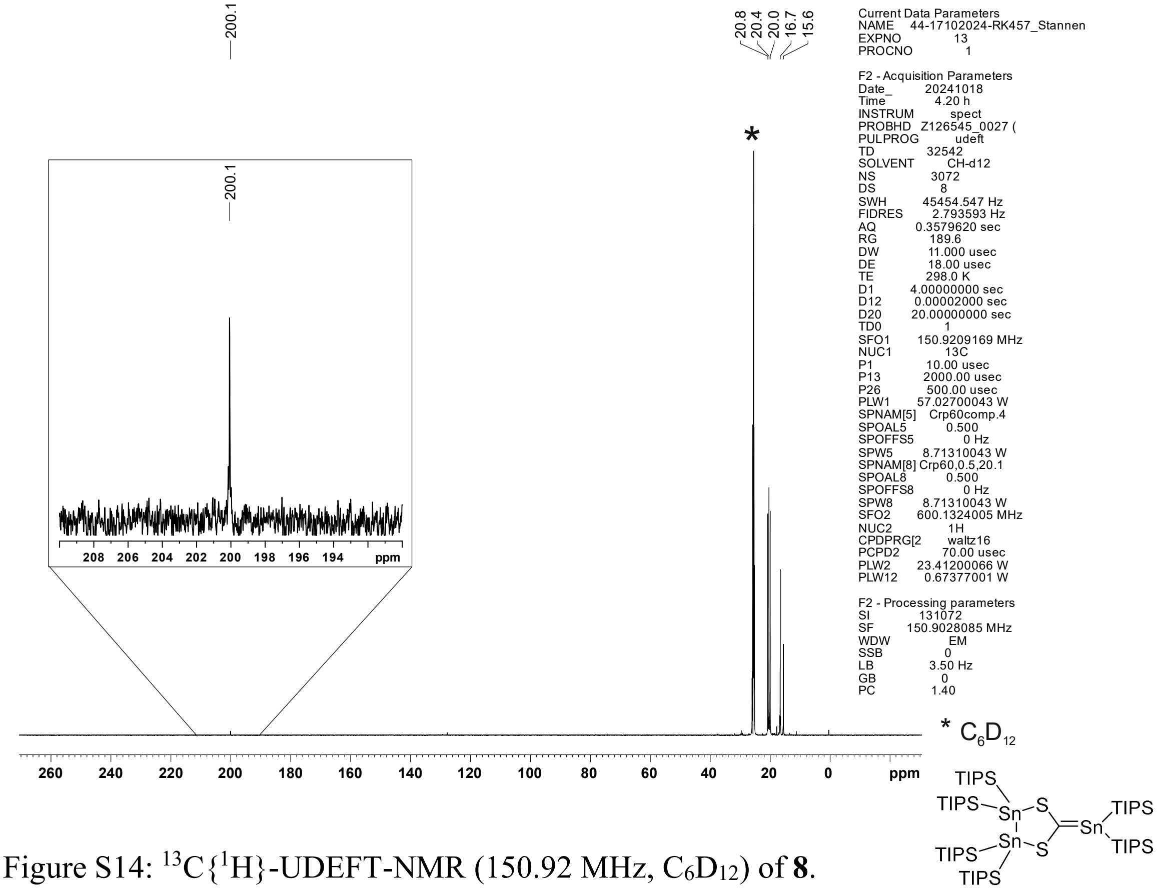

8

Figure S14: ^{13}C{^{1}H}-UDEFT-NMR (150.92 MHz, C_6D_{12}) of **8**.

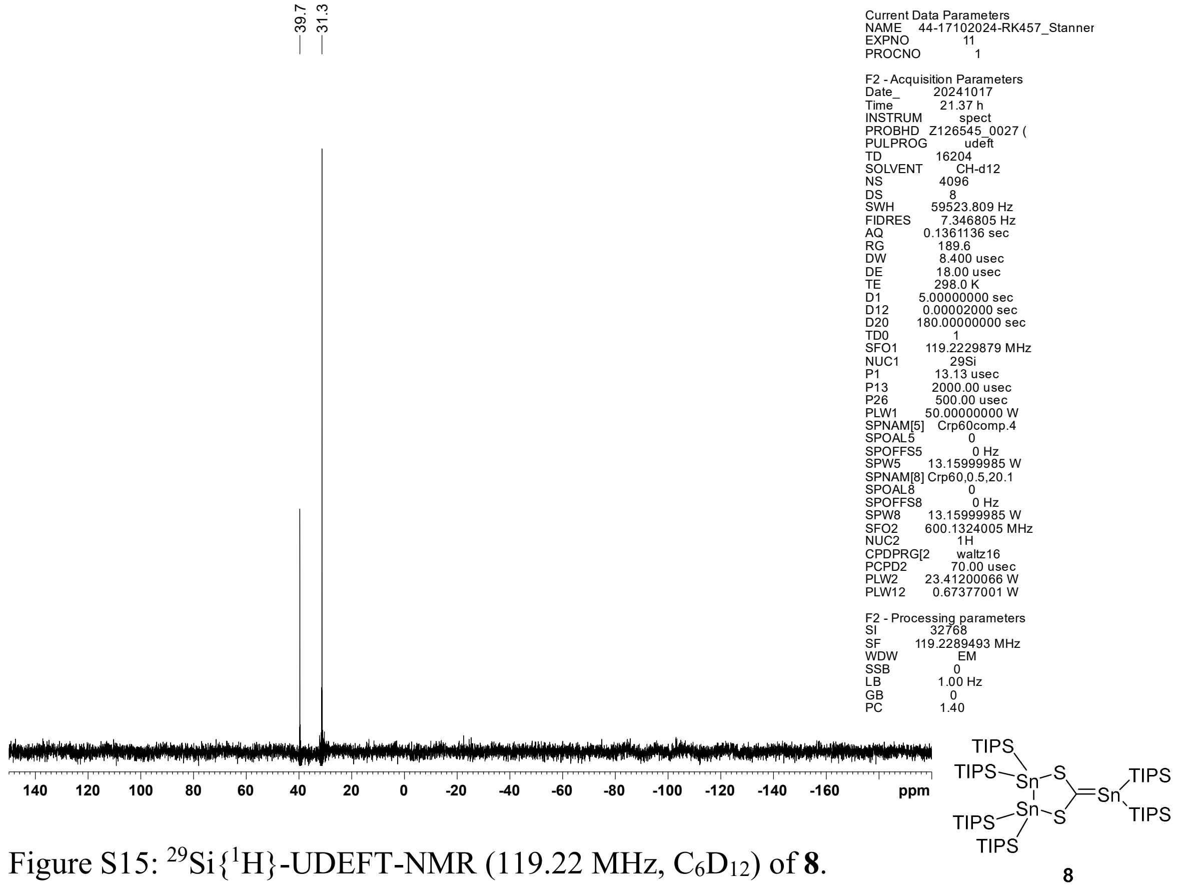

8

Figure S15: ^{29}Si{^{1}H}-UDEFT-NMR (119.22 MHz, C_6D_{12}) of **8**.

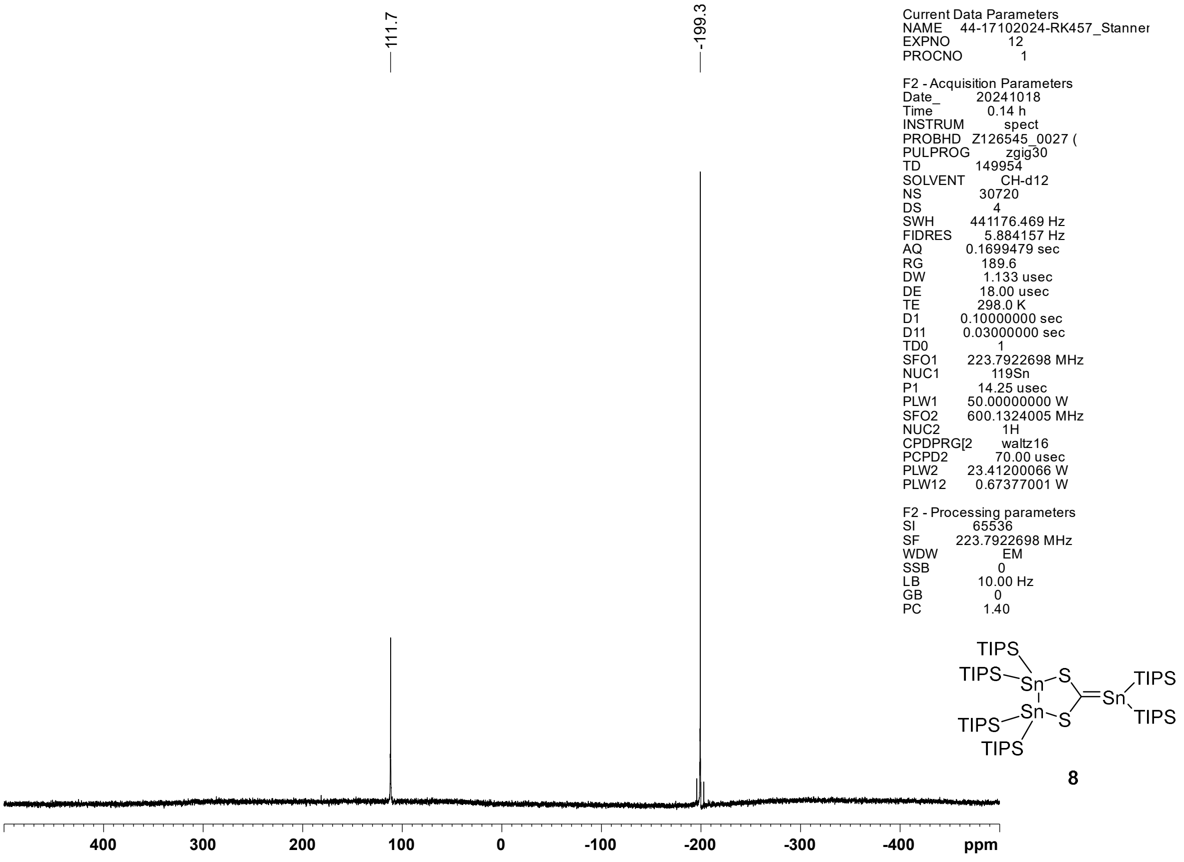

Figure S16: $^{119}Sn\{^{1}H\}$-NMR (223.79 MHz, C_6D_{12}) of **8**.

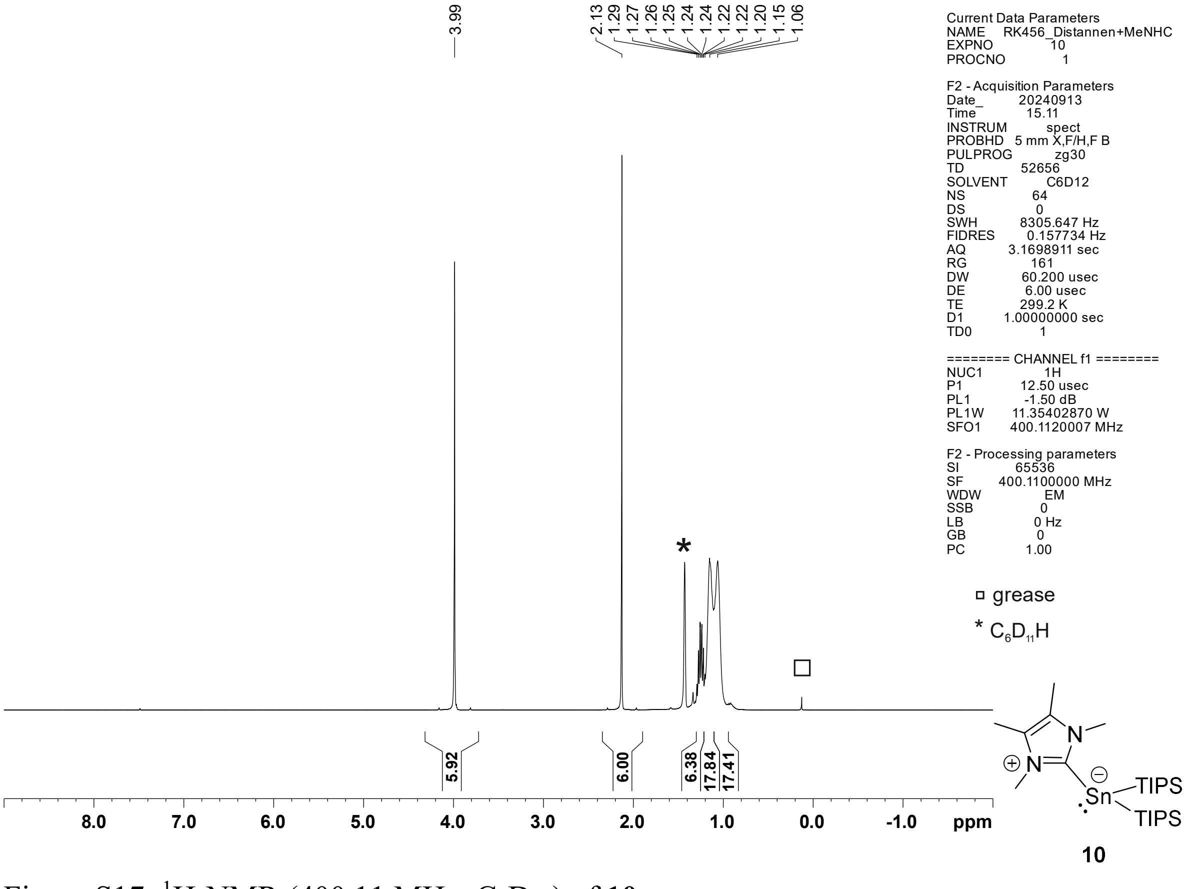

Figure S17: ^{1}H-NMR (400.11 MHz, C_6D_{12}) of **10**.

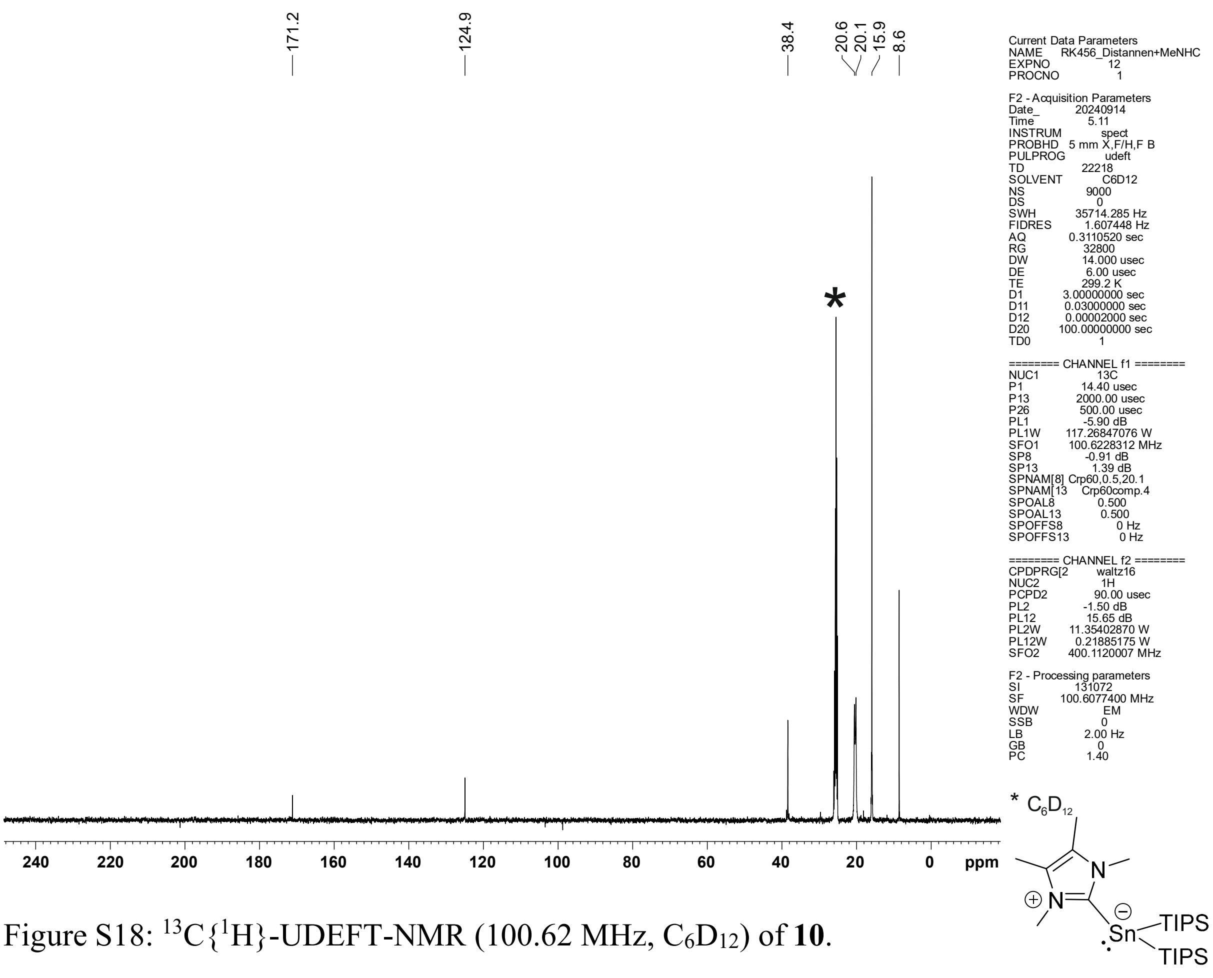

Figure S18: $^{13}C\{^{1}H\}$-UDEFT-NMR (100.62 MHz, C_6D_{12}) of **10**.

10

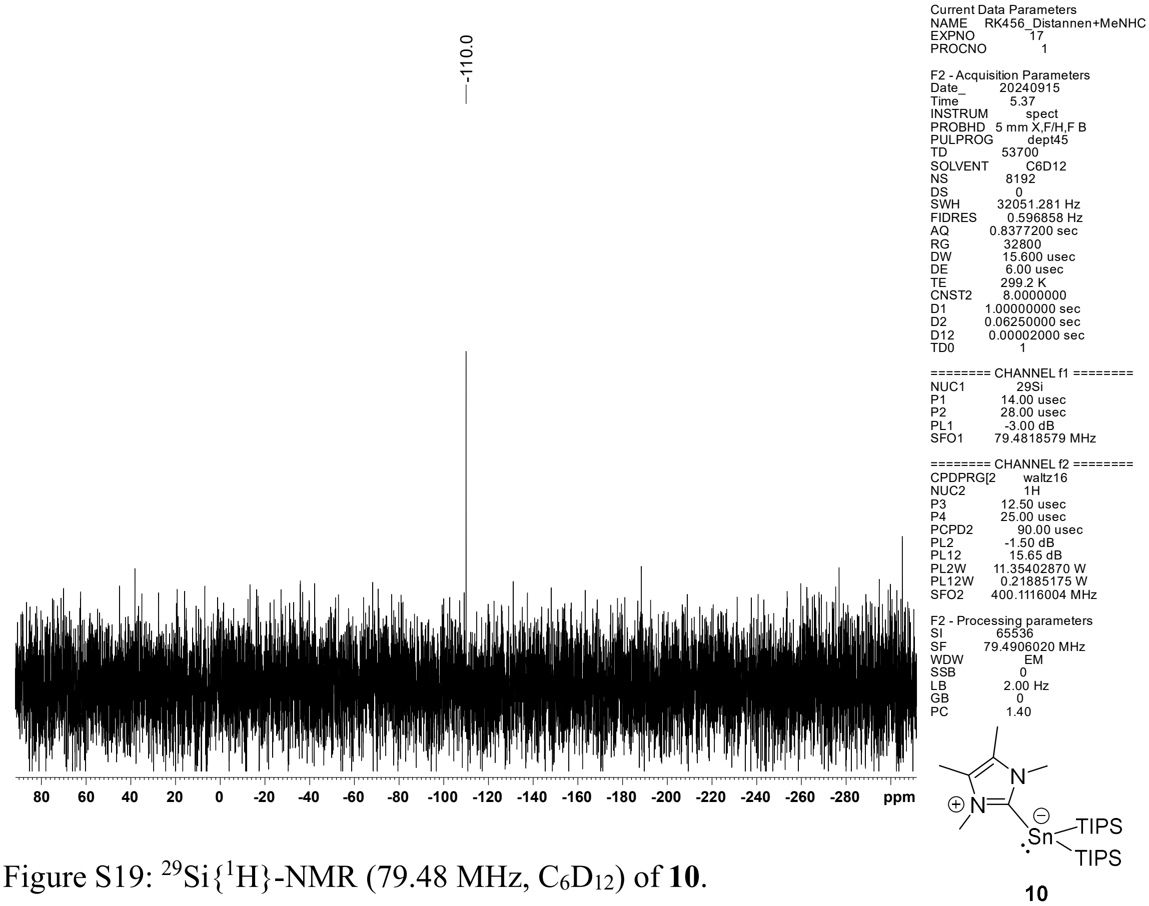

Figure S19: $^{29}Si\{^{1}H\}$-NMR (79.48 MHz, C_6D_{12}) of **10**.

10

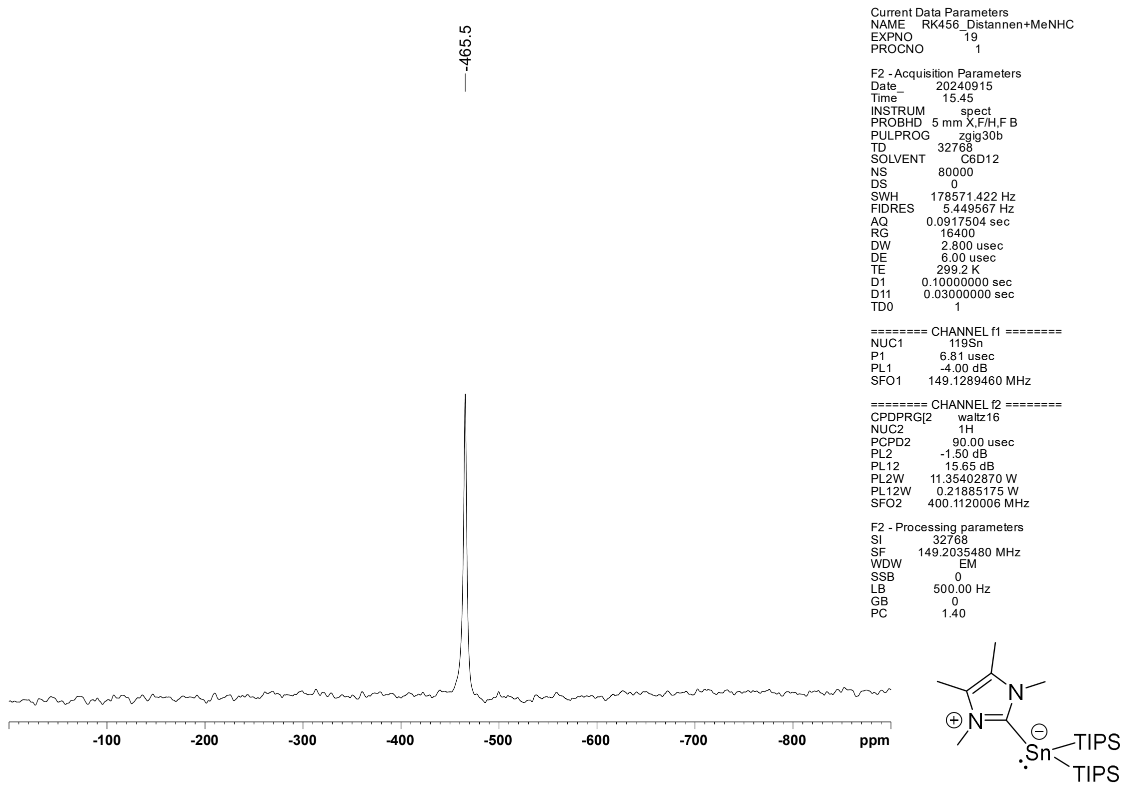

10

Figure S20: $^{119}Sn\{^{1}H\}$-NMR (149.13 MHz, C_6D_{12}) of **10**.

2 UV/Vis data

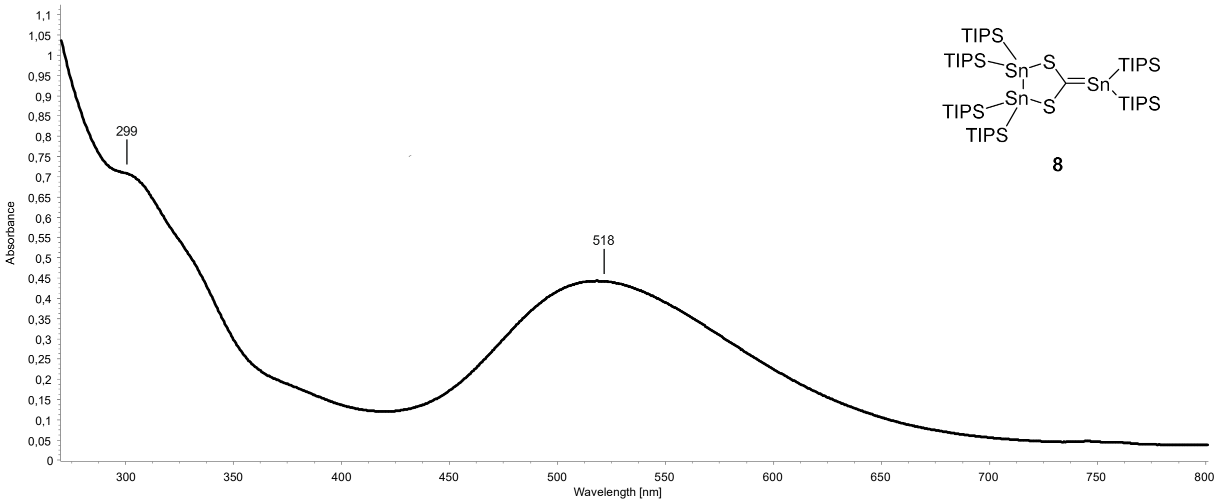

Figure S21: UV/Vis spectrum of **8** measured in pentane.

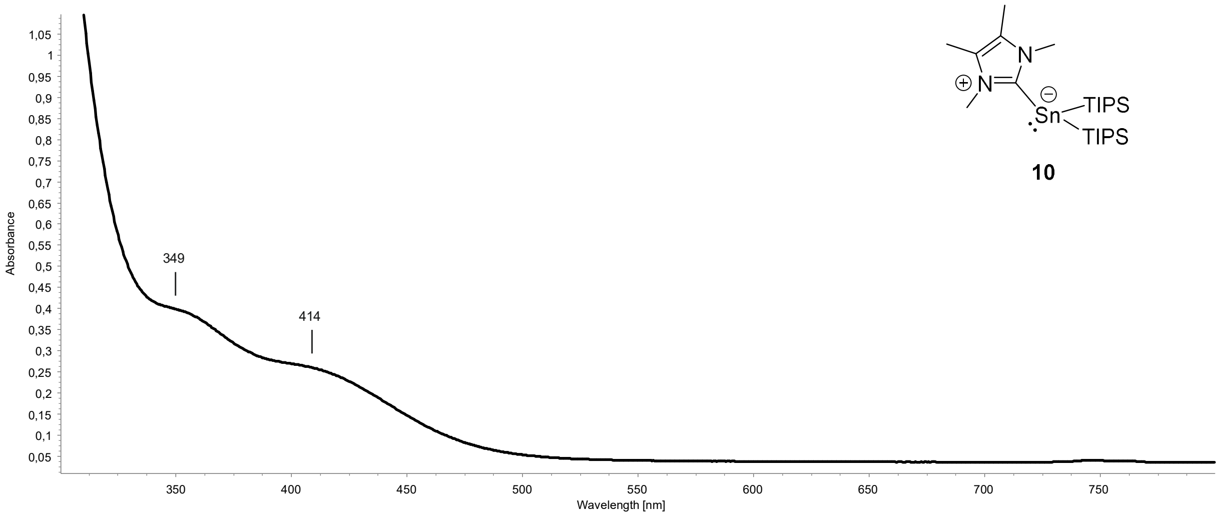

Figure S22: UV/Vis spectrum of **10** measured in pentane.

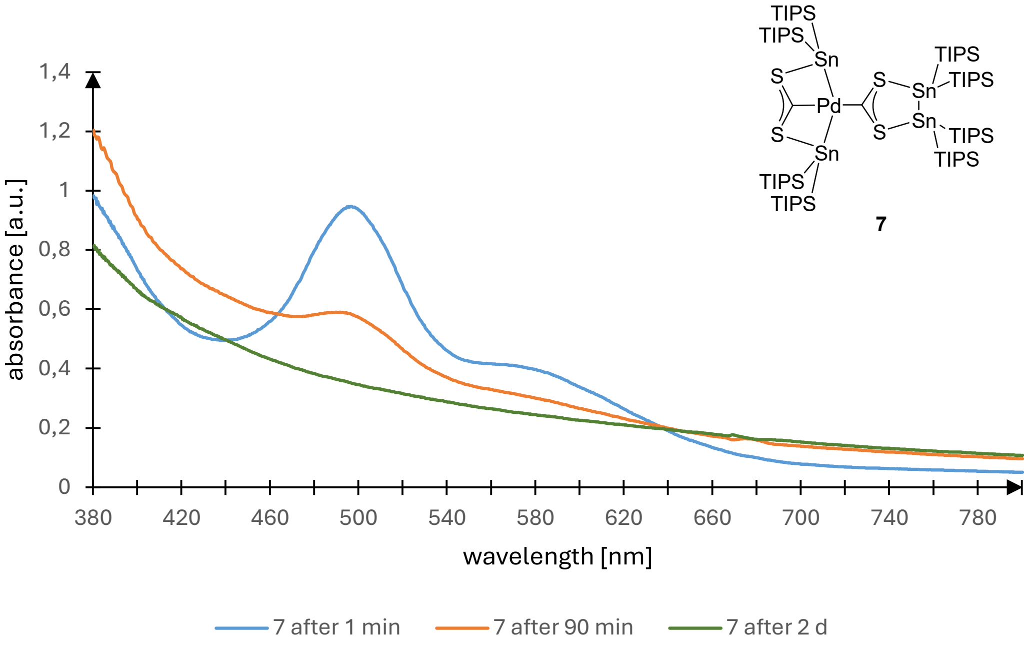

Figure S23: UV/Vis spectrum of **7** measured in CS_2 over two days.

3 EDX-Measurements

Table S1: Results of the EDX measurement on the crystals of **7** at point 118 (Figure 24S) at an acceleration voltage of 10 kV and a tilt angle of 30°.

Element	Norm Wt. %	Norm Wt. % calculated	Norm At. %	Norm At. % calculated	Error (Wt % 3 σ)
Sulfur	13.99	13.73	23.89	23.52	0.62
Tin	50.51	50.83	23.30	23.52	1.97
Palladium	11.44	11.39	5.89	5.88	9.57
Silicon	24.07	24.05	46.93	47.06	1.05

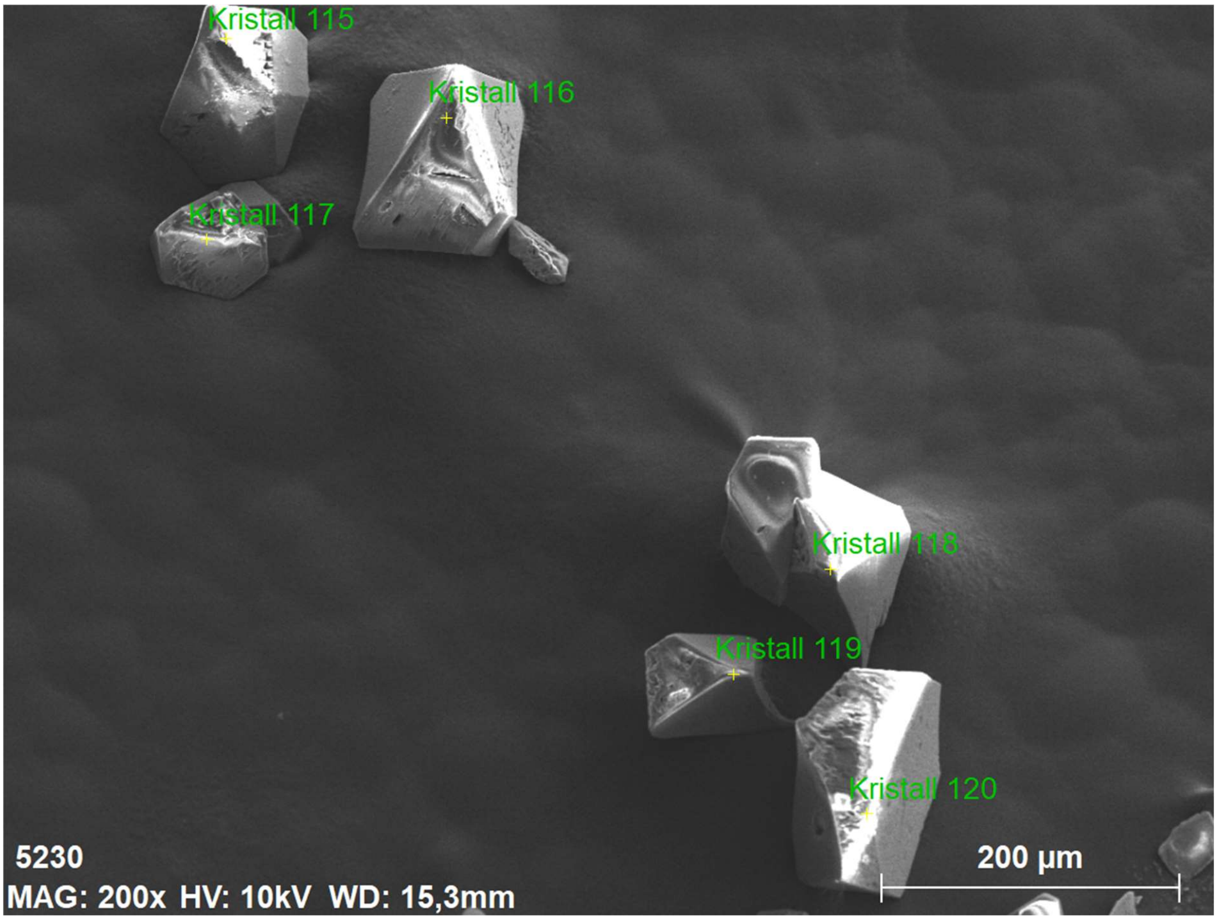

Figure S24: REM pictures of compound **7** and point of measurements.

4 Attempts to capture an SHC, as well as C-C bond cleavage in tetrathiaethylene 2 with metal complexes

The procedure was conducted in the following manner: distannene **1** was intermixed with the metal precursors and both reactants dissolved in a suitable solvent (usually *n*-pentane, in some cases benzene or thf). The addition of CS_2 to this solution occurred in the direct presence of the metal complexes, thereby enabling the carbene formed in situ subsequently engage in adduct formation. For all metal complexes investigated, no adduct formation could be observed and dimer 2 formed instantaneously. The resulting reaction mixture was analyzed further. However, even after heating and/or exposure to light, no C-C bond cleavage of dimer **2** with formation of an SHC-metal complex was observed. The reactants which were employed in the attempted cleavage of a C-C bond in order to facilitate the transfer of an SHC as well as the capture of the intermediately formed SHC are described hereinafter:

$W(CO)_6$, $Ru_3(CO)_{12}$, $Fe(CO)_5$, $Cr(CO)_6$, $Pd(PPh_3)_4$, $Pt(PPh_3)_4$, $Ni(PPh_3)_4$, $CpCo(CO)_2$., $Mo(CO)_4(pip)_2$, $[RhCl(CO)_2]_2$, $TiCl_4(thf)_2$, $TiCl_3(thf)_3$, $HfCl_4(thf)_2$, $ZrCl_4(thf)_2$, $Cr(CO)_3(NCMe)_3$.

5 Possible mechanism for the formation of 7

A potential mechanism for the formation of **7** involves a prior reaction to the target compound SHC-$Pd(PPh_3)_2$. In a subsequent reaction, the $(SHC)_2Pd$ complex is formed under elimination of $Pd(PPh_3)_4$. This hypothesis is supported by the isolation of crystalline $Pd(PPh_3)_4$ from the reaction solution. The $(SHC)_2Pd$ complex then undergoes oxidative addition under Sn-Sn bond cleavage, leading to the formation of X. Both, an inter- and an intramolecular oxidative addition are possible pathways.

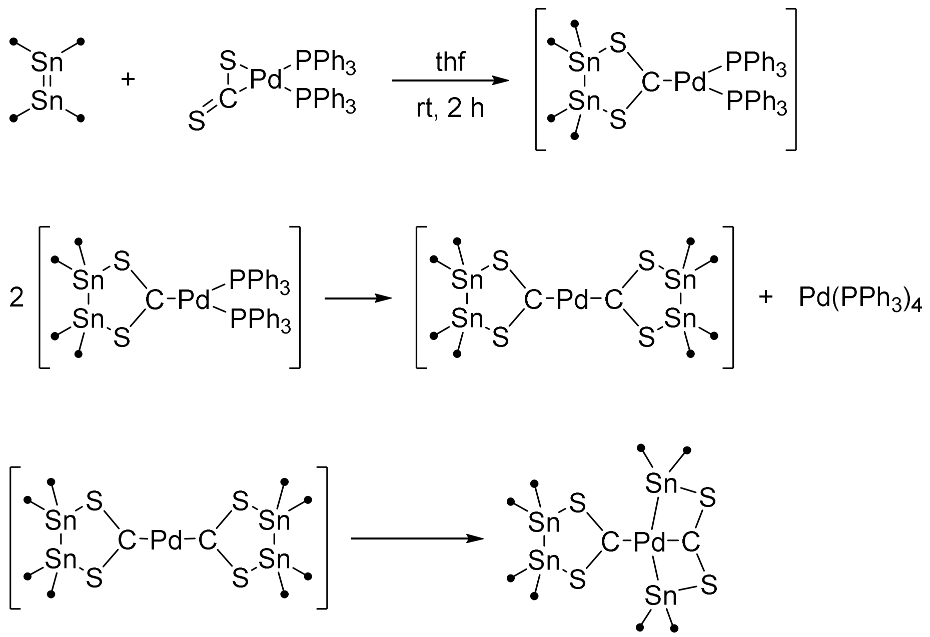

Scheme S1: Possible formation of the Pd complex **7**.

6 Crystallographic data

6.1 Crystal structure of **10**

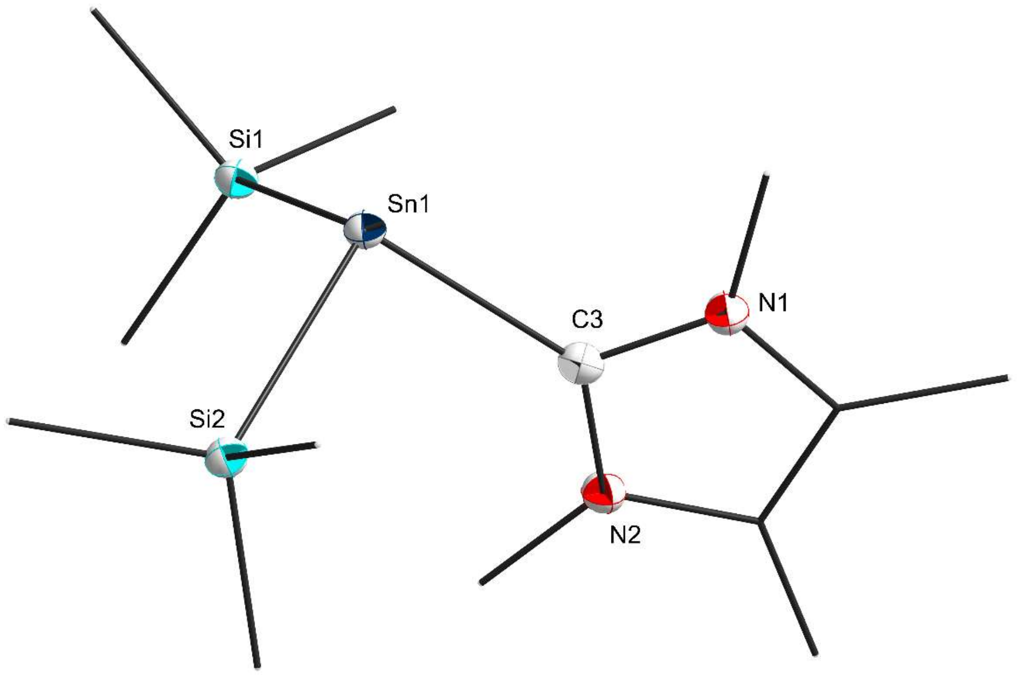

Figure S25: Molecular structure of **10**. Hydrogen atoms and methyl groups of the TIPS substituents are omitted for clarity. Carbon atoms are displayed as wire model, except C12. Silicon (light blue), carbon (grey), nitrogen (yellow) and tin (dark blue) atoms are shown as their displacement ellipsoids with 50% probability. Selected bond lengths [pm] and angles [°]:

Sn1-C3: 230.18(11), Sn1-Si2: 264.25(3), Sn1-Si1: 264.97(3), N2-C3: 135.53(14), N1-C3: 135.95(14), C3-Sn1-Si2: 98.21(3), C3-Sn1-Si1: 98.85(3), Si2-Sn1-Si1: 111.89(1), N2-C3-N1: 103.86(9), N2-C3-Sn1: 133.90(8), N1-C3-Sn1: 122.16(8).

6.2 Crystal structure of a Pd_6 complex

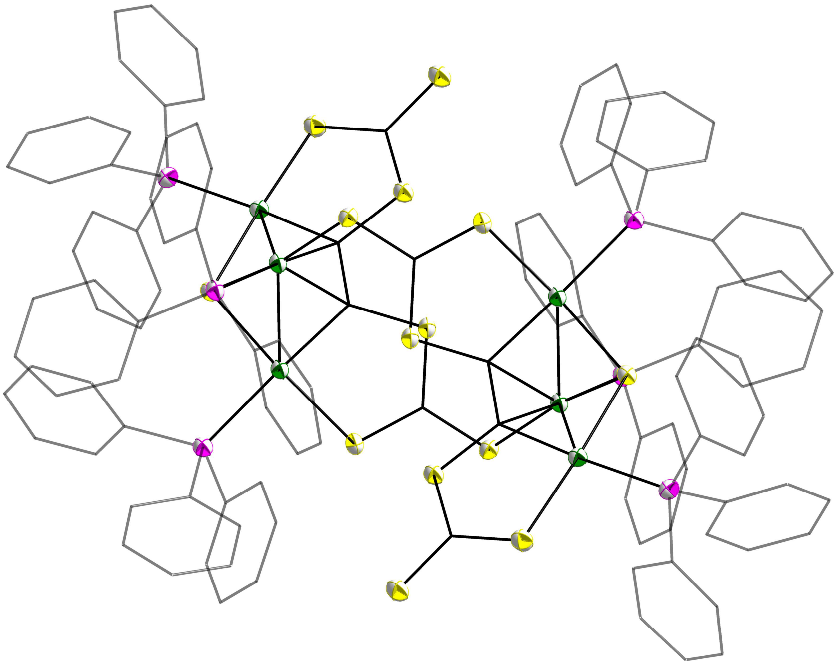

Figure S26: Decomposition product of $(PPh_3)_2Pd\text{-}CS_2$ crystallized from thf. Molecular structure of the decomposition product. Hydrogen atoms are omitted for clarity. Phosphorus (violet), palladium (green) and sulfur (yellow) atoms are shown as their displacement ellipsoids with 50% probability.

6.3 Crystallographic data for compounds **2**, **5**, **7**, **8** and **10** and the Pd_6 complex.

Table S2: Data of crystal structure determination for compounds **2**, **5**, **7** and **8**.

	CS_2-Dimer **2**	BCF-Adduct **5**	Pd-Complex **7**	Stannaethene **8***
Device	A	B	C	A
empirical formula	$C_{74}H_{168}S_4Si_8Sn_4$	$C_{55}H_{84}BF_{15}S_2Si_4Sn_2$	$C_{74}H_{168}PdS_4Si_8Sn_4$	$C_{55}H_{126}S_2Si_6Sn_3$
M [g/mol]	1885.79	1454.89	1992.19	1376.28
T [K]	100(2)	120(2)	149.99(10)	100.08
λ [Å]	0.71073	0.71073	1.54184	0.71073
crystal system	triclinic	monoclinic	monoclinic	triclinic
space group	$P\bar{1}$	$P2_1/n$	I2/a	$P\bar{1}$
Z	1	4	4	2
a [Å]	12.3205(11)	13.2836(3)	24.5326(2)	14.4112(6)
b [Å]	12.9200(11)	21.7744(5)	14.90050(10)	15.3274(6)
c [Å]	16.9960(15)	22.2690(5)	29.7469(2)	19.4710(8)
α [°]	87.705(2)	90	90	88.3490(10)
β [°]	70.119(2)	93.2710(10)	117.4400(10)	86.4880(10)
γ [°]	70.852(2)	90	90	61.9570(10)
V [Å^3]	2395.6(4)	6430.6(3)	9650.54(14)	3788.8(3)
D_c [g/cm^3]	1.307	1.503	1.371	1.206
μ [mm^{-1}]	1.252	0.994	11.606	1.159
F(000)	988	2968	4136	1444
crystal size [mm]	0.178 x 0.062 x 0.043	0.31 x 0.28 x 0.25	0.149 x 0.101 x 0.069	0.36 x 0.228 x 0.212
θ range [°]	2.023 – 26.372	3.072 – 31.075	3.348 – 74.498	1.603 – 30.566
limiting indices	$-15 \leq h \leq 15$	$-19 \leq h \leq 18$	$-29 \leq h \leq 30$	$-20 \leq h \leq 20$
	$-16 \leq k \leq 16$	$-31 \leq k \leq 23$	$-18 \leq k \leq 18$	$-21 \leq k \leq 21$
	$-21 \leq l \leq 21$	$-32 \leq l \leq 29$	$-37 \leq l \leq 37$	$-27 \leq l \leq 27$
reflections collected	40613	153492	192324	88096
independent reflections	9775	20592	9877	23173
R_{int}	0.0827	0.0703	0.0415	0.0216
Completeness [%]	95.2	99.8	100	99.8
absorption correction	multi-scan	multi-scan	gaussian	multi-scan
max., min. transmission	0.6573, 0.7457	0.7046, 0.7462	0.305, 0.676	0.6852, 0.7461
parameter/restraints	431 / 0	736 / 0	436 / 0	764 / 417
R_1, ωR_2 [I > 2σ(I)]	0.0605, 0.1428	0.0422, 0.0572	0.0162, 0.0403	0.0512, 0.1510
R_1, ωR_2 (all data)	0.0853 0.1580	0.0853, 0.0659	0.0165, 0.0404	0.0622, 0.1648
GooF on F^2	1.083	0.997	1.058	1.031
peak / hole [e·Å^{-3}]	3.651, −1.088	0.762, −0.976	0.457, −0.561	1.904, −0.789
CCDC	2411408	2411412	2411411	2411413

*Electron density for disordered solvent molecules was removed with PLATON/SQUEEZE[3-4]

Table S3: Data of crystal structure determination for compound **10** and the Pd_6 complex.

	NHC-Adduct **10**	Pd_6-Complex
Device	B	A
empirical formula	$C_{25}H_{54}N_2Si_2Sn$	$C_{148}H_{154}O_8P_6Pd_6S_{14}$
M [g/mol]	557.57	3333.76
T [K]	100(2)	100.01
λ [Å]	0.71073	0.71073
crystal system	triclinic	Monoclinic
space group	$P\bar{1}$	$P2_1/n$
Z	2	2
a [Å]	8.5508(2)	13.8103(9)
b [Å]	10.9327(2)	22.9982(15)
c [Å]	17.4744(3)	21.4865(14)
α [°]	75.1800(10)	90
β [°]	80.2220(10)	92.994(2)
γ [°]	74.2390(10)	90
V [Å^3]	1510.98(5)	6815.1(8)
D_c [g/cm^3]	1.226	1.625
μ [mm^{-1}]	0.939	1.119
F(000)	592	3392.0
crystal size [mm]	0.35 x 0.30 x 0.27	0.079 × 0.067 × 0.026
θ range [°]	3.284 – 28.294	2.596 – 50.054
limiting indices	$-11 \leq h \leq 11$	$-16 \leq h \leq 16$
	$-14 \leq k \leq 14$	$-27 \leq k \leq 27$
	$-20 \leq l \leq 23$	$-25 \leq l \leq 25$
reflections collected	59510	75638
independent reflections	7485	12053
R_{int}	0.0282	0.1110
Completeness [%]	99.6	100
absorption correction	multi-scan	multi-scan
max., min. transmission	0.7175, 0.7457	0.7454, 0.6709
parameter/restraints	287 / 0	820 / 73
R_1, ωR_2 [$I > 2\sigma(I)$]	0.0163, 0.0409	0.0685, 0.1199
R_1, ωR_2 (all data)	0.0178, 0.0415	0.1144, 0.1425
GooF on F^2	1.062	1.093
peak / hole [e·Å^{-3}]	0.406, −0.207	1.57, –0.94
CCDC	2411410	2411409

7 Theoretical Calculations

7.1 Geometry optimized structures

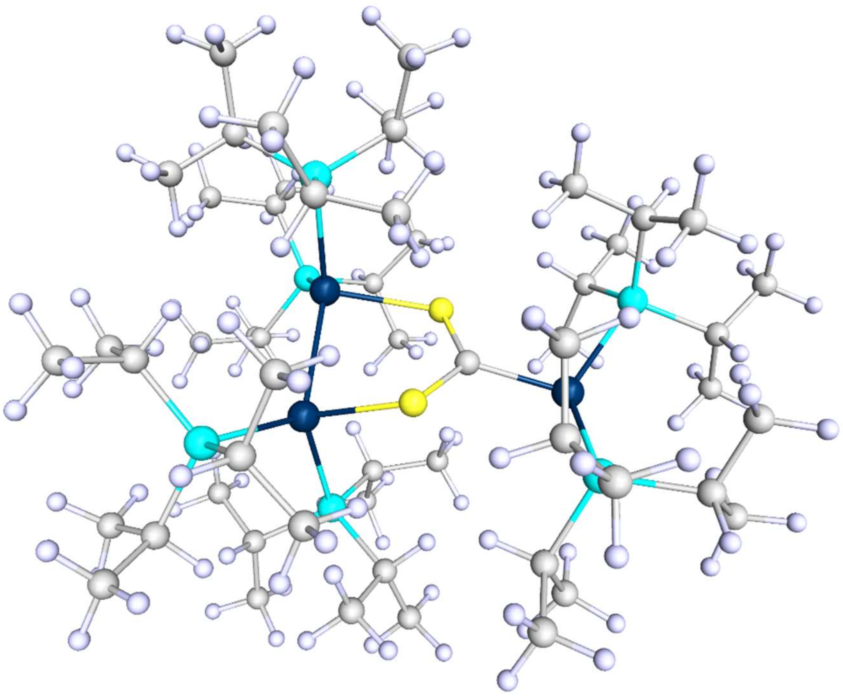

Figure S27: Geometry optimized structure for **8**: Total energy: −5345.71167627976 H, HOMO-LUMO-Gap: 2.622 eV.

Table S4: Atomic coordinates of **8**

Sn	9.076508	4.690641	15.663097
Sn	7.321573	4.776175	13.458061
S	8.633922	7.095790	16.073845
Si	11.676465	4.767235	15.246919
Si	8.297539	3.568879	17.918968
S	8.119725	7.089292	13.060089
Si	8.012778	3.621316	11.198391
Si	4.753705	5.091354	13.924550
C	8.241876	7.892628	14.585650
C	12.339968	6.089733	16.436320
C	12.330902	3.032393	15.664759
C	11.964479	5.287652	13.443349
C	7.705374	4.849711	19.194702
C	9.788560	2.649045	18.684753
C	6.831880	2.428347	17.497109
C	9.371709	2.351144	11.602271
C	6.446686	2.860577	10.410294
C	8.717843	4.872955	9.950294
C	3.962016	3.425709	13.456305
C	4.174756	6.504166	12.797085
C	4.530211	5.564775	15.750136
Sn	7.749900	9.909752	14.610693
H	11.715454	6.963960	16.217403
C	12.113199	5.720632	17.895446
H	14.057669	7.338715	16.844398
H	14.484074	5.675579	16.456072
H	14.250698	3.544949	16.568177
H	14.124089	1.874169	16.024705
H	14.356661	3.167109	14.850789
H	10.670393	2.038241	14.662310
H	12.134914	2.123966	13.694379
H	12.030877	0.975527	15.028211
H	10.806818	7.126423	13.675751
H	12.551543	7.358304	13.787794
H	11.800116	7.071457	12.224269
H	13.415295	3.763839	12.834510
H	13.364039	5.189679	11.798312
H	14.146592	5.274820	13.373322
H	9.698357	5.412498	19.915892
H	8.997986	6.538242	18.749148
H	8.402901	6.505079	20.403363
H	5.603199	4.853992	18.590435
H	6.078879	6.217458	19.602909
H	6.571067	6.171192	17.912714
H	10.154166	1.418203	16.901876
H	11.128279	0.984887	18.307801
H	9.424383	0.555574	18.252526
H	9.506780	3.336250	20.748731
H	4.805251	7.286334	17.035168
H	2.972203	4.129143	16.310519
H	3.144741	5.498338	17.408201
H	2.354325	5.714736	15.850232
C	8.272619	12.844058	16.664771
C	8.533000	10.027534	18.173726
C	10.814783	11.097882	16.404371
C	8.977133	10.003613	11.022890
C	7.473657	12.546434	12.074336
C	5.957438	9.797810	11.493990
H	8.392669	13.197011	15.631581
C	9.088611	13.774060	17.559988
C	6.788836	12.928086	17.007128
H	9.121296	9.115600	18.011932
C	9.037080	10.685771	19.456229
C	7.079745	9.593049	18.318830
H	11.195709	11.594090	17.308677
C	11.240486	11.918777	15.193604
C	11.419342	9.701175	16.332430
H	8.643923	8.991669	10.758076
C	9.132547	10.802694	9.728660
C	10.322655	9.865126	11.722959
H	7.252480	12.803028	11.028526
C	8.818589	13.166150	12.442230

C 13.797119 6.487072 16.206593
H 11.915541 2.830621 16.659329
C 13.847988 2.906140 15.780878
C 11.760653 1.989078 14.713006
H 11.164831 4.791899 12.880234
C 11.767403 6.791987 13.281145
C 13.294589 4.848731 12.837291
H 7.497594 4.230234 20.079328
C 8.765400 5.873654 19.585207
C 6.415524 5.553702 18.799409
H 10.630428 3.337996 18.543682
C 10.141193 1.337184 17.990226
C 9.643867 2.410938 20.187708
H 6.097424 3.114095 17.052922
C 7.137581 1.358297 16.456202
C 6.194629 1.803564 18.736409
H 10.162666 2.964534 12.055910
C 9.952034 1.690962 10.353381
C 8.971990 1.297871 12.628184
H 5.673502 3.624778 10.562102
C 5.971134 1.576031 11.082070
C 6.574703 2.635010 8.903990
H 8.869016 4.255371 9.052846
C 10.067672 5.453598 10.349252
C 7.745175 5.988674 9.583027
H 4.356949 3.224611 12.453156
C 4.462163 2.315735 14.371737
C 2.439220 3.404794 13.347802
H 4.837863 7.339416 13.056805
C 2.735465 6.948041 13.055752
C 4.382440 6.187416 11.322700
H 5.295740 4.988562 16.282747
C 4.837913 7.042815 15.969092
C 3.176573 5.200503 16.354343
Si 8.923608 11.052989 16.616700
Si 7.549927 10.642633 12.124657
H 11.061020 5.519739 18.101830
H 12.686563 4.833663 18.182852
H 12.422897 6.536330 18.557427
H 13.997557 6.780568 15.175570
H 8.794443 1.760048 20.411118
H 10.539905 1.918470 20.580711
H 7.599611 1.779607 15.558950
H 7.814617 0.597773 16.845450
H 6.221509 0.847747 16.145091
H 5.918071 2.549510 19.484627
H 5.286453 1.253248 18.467864
H 6.872648 1.090621 19.213424
H 10.291855 2.420441 9.615004
H 10.809063 1.060148 10.612636
H 9.214352 1.046323 9.867658
H 8.551878 1.746663 13.532617
H 8.227248 0.608326 12.229938
H 9.838296 0.701201 12.928571
H 5.961456 1.640845 12.171395
H 4.957322 1.320924 10.756001
H 6.614714 0.736350 10.809224
H 6.800393 3.552354 8.358773
H 7.360375 1.910984 8.672366
H 5.638266 2.235204 8.500254
H 10.816560 4.680302 10.535016
H 10.454793 6.101911 9.555750
H 9.978893 6.062427 11.251232
H 6.777811 5.611504 9.244495
H 7.563098 6.651046 10.433248
H 8.157190 6.606318 8.777917
H 5.553407 2.286373 14.416900
H 4.102975 2.444121 15.396473
H 4.119797 1.334265 14.026661
H 2.077274 4.071850 12.564402
H 2.093384 2.394925 13.100356
H 1.952880 3.694749 14.281264
H 2.568140 7.244539 14.091927
H 2.485408 7.809057 12.427389
H 2.017828 6.160446 12.818940
H 5.422707 5.939447 11.105470
H 3.761895 5.345602 10.999701
H 4.116778 7.045731 10.696582
H 5.824868 7.327374 15.597623
H 4.106652 7.683665 15.472025
C 6.382294 13.145880 12.955102
H 6.159552 8.744936 11.732552
C 5.758155 9.894912 9.983991
C 4.680040 10.195512 12.222218
H 10.148844 13.777193 17.298188
H 8.726008 14.804491 17.476407
H 9.011082 13.488431 18.611563
H 6.184921 12.246088 16.401784
H 6.613286 12.675252 18.056017
H 6.407399 13.942508 16.849498
H 10.082524 10.996822 19.385217
H 8.444857 11.569262 19.709333
H 8.956803 9.992586 20.300947
H 6.724407 9.066615 17.429622
H 6.966714 8.914617 19.171608
H 6.415268 10.443727 18.487018
H 10.856432 12.941250 15.221134
H 12.332149 11.977146 15.125726
H 10.881156 11.454594 14.271023
H 11.222115 9.115898 17.232850
H 11.006328 9.145141 15.484887
H 12.505706 9.752398 16.198894
H 8.195803 10.913668 9.180288
H 9.516778 11.807058 9.923046
H 9.848906 10.310776 9.061232
H 10.246821 9.260805 12.628584
H 11.055567 9.390849 11.060772
H 10.728934 10.839127 12.008328
H 9.631528 12.832027 11.796399
H 8.772013 14.258851 12.379917
H 9.096378 12.910919 13.468233
H 5.381135 12.837200 12.656900
H 6.514242 12.854203 14.001662
H 6.417834 14.240400 12.919507
H 6.584001 9.446801 9.428826
H 4.843591 9.370126 9.684914
H 5.653428 10.933282 9.653674
H 4.800246 10.179556 13.308987
H 4.350562 11.196663 11.934501
H 3.865639 9.510180 11.966990

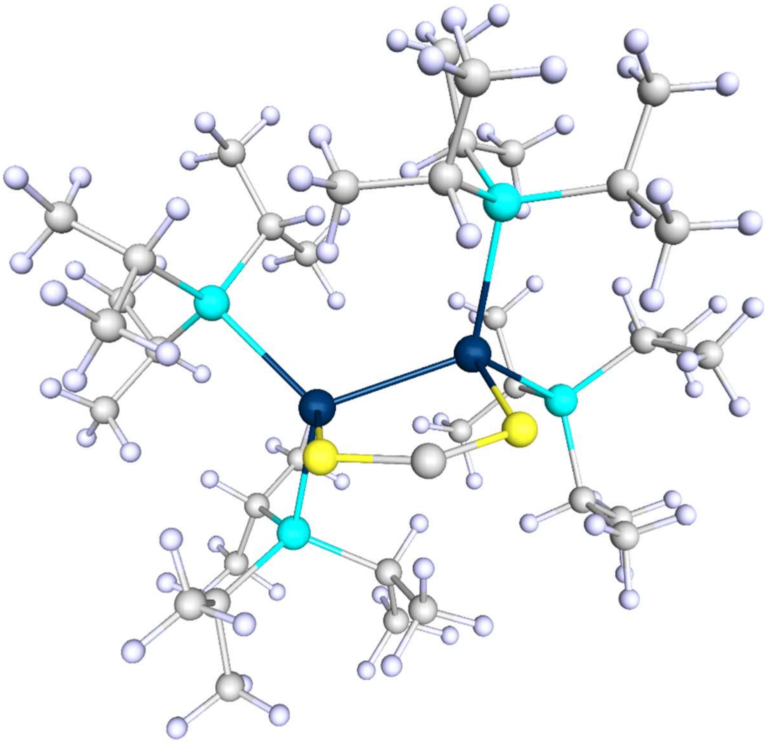

Figure S28: Geometry optimized structure for **4**: Total energy: −3841.85370653102 H, HOMO-LUMO-Gap: 3.743 eV.

Table S5: Atomic coordinates of **4**

Sn 9.968984 6.391052 10.078404	C 5.520708 5.400173 5.156518	H 4.737239 5.995605 9.215183
Sn 7.953115 5.436894 8.351062	C 6.165598 7.626217 4.186919	H 3.982756 5.657456 7.657074
S 9.069374 4.992815 12.046809	C 7.034319 10.127575 6.773254	H 3.625601 7.685081 6.006057
Si 12.513315 5.756945 9.849639	H 7.929962 8.838770 8.199474	H 4.408597 9.229088 6.338819
Si 9.465835 8.698659 11.278944	C 9.009509 8.613956 6.381583	H 3.045169 8.695127 7.322369
S 6.618984 4.563267 10.398392	H 7.826659 4.185049 4.867612	H 4.482544 5.718800 5.275597
Si 6.586714 7.258936 7.036534	C 8.864961 2.454593 4.228075	H 5.714943 4.640871 5.918825
Si 8.436638 3.237460 6.978070	C 9.904651 4.633495 4.893928	H 5.598728 4.914690 4.177651
C 7.540075 4.511036 11.737472	C 7.292998 0.605434 6.811792	H 6.199547 7.167408 3.192602
C 13.451662 6.884102 11.068079	C 5.863965 2.272612 8.003906	H 6.882686 8.448794 4.190392
C 12.809429 3.894032 10.107147	H 10.583857 3.409233 8.006256	H 5.169721 8.054927 4.311332
C 13.110710 6.188457 8.097377	C 10.765740 1.477284 7.141603	H 7.731906 10.955770 6.941050
C 10.634825 8.883871 12.777408	C 9.575624 2.045341 9.288604	H 6.149018 10.319489 7.382872
C 9.763372 10.100134 10.019431	H 13.906155 5.575767 12.734890	H 6.731260 10.168125 5.724095
C 7.663784 8.563034 11.875634	H 12.270757 6.224582 12.793589	H 8.858099 8.565817 5.300113
C 4.877996 7.642057 7.788380	H 13.646205 7.252770 13.199355	H 9.522165 7.695454 6.682841
C 6.477068 6.584158 5.259565	H 15.396763 7.770786 11.417045	H 9.693061 9.447561 6.576774
C 7.695217 8.800149 7.129877	H 15.072342 7.465888 9.711475	H 8.959559 2.778425 3.185812
C 8.754811 3.672176 5.145798	H 15.478660 6.129298 10.785901	H 9.742042 1.845331 4.456724
C 6.910007 2.082421 6.915009	H 12.924767 3.690641 12.272464	H 10.868240 4.171431 5.118705
C 9.945772 2.524224 7.887603	H 14.924404 3.913795 10.626041	H 9.826574 5.539802 5.500154
H 12.964755 7.860966 10.948443	H 14.635676 3.874839 8.885544	H 9.930500 4.941665 3.843070
C 13.302174 6.461461 12.525318	H 14.384477 2.425535 9.859374	H 5.008704 1.610060 7.831968
C 14.927180 7.070135 10.718257	H 11.427314 5.335655 7.051909	H 11.637617 1.185659 7.738650
C 12.336513 3.313351 11.434403	H 12.780379 4.216511 7.213822	H 11.135807 1.850463 6.184724
H 12.204171 3.431715 9.317546	H 13.339368 8.339715 8.415387	H 8.915790 1.175760 9.256981
C 14.267693 3.514412 9.848821	H 11.794585 7.870307 7.703008	H 10.470496 1.751483 9.845303
H 14.196501 6.025337 8.128935	H 11.063843 11.028990 12.589996	H 12.855864 5.537408 6.044040
C 12.517297 5.263818 7.048940	H 11.427617 10.338315 14.168431	H 7.968458 11.297050 10.358555

C 12.865165 7.643102 7.721129	H 11.063502 7.959366 14.689802	H 9.759049 10.657213 13.710852
H 11.629734 8.626748 12.397171	H 10.238672 6.874673 13.567322	H 5.483101 3.296317 8.037900
C 10.721120 10.307105 13.331672	H 9.342501 8.149323 14.367399	H 6.262367 2.045650 8.994530
C 10.302612 7.909968 13.904016	H 9.369602 11.845694 11.275421	H 11.288628 3.537446 11.637044
C 9.052667 11.416732 10.322258	H 9.273877 12.155605 9.543915	H 12.445643 2.223544 11.430627
H 9.347301 9.717974 9.081839	H 11.417268 10.994067 8.933278	H 10.191304 0.569049 6.947116
C 11.253918 10.318294 9.779361	H 11.768179 9.382857 9.553748	H 6.443241 2.365280 5.961018
C 7.231709 9.634907 12.875195	H 11.746446 10.761808 10.647286	H 13.256185 7.858037 6.720999
C 6.671808 8.483999 10.724921	H 7.277482 10.638675 12.446616	H 6.195287 9.460583 13.183769
H 7.637735 7.596710 12.395921	H 7.842364 9.632064 13.778394	H 7.986658 1.810041 4.288177
C 4.166678 6.439287 8.398790	H 5.660304 8.288837 11.095502	H 9.069093 2.817644 9.872046
H 5.098578 8.343073 8.602018	H 6.919079 7.676786 10.031843	H 8.015657 0.406405 6.018816
C 3.948978 8.356165 6.805130	H 6.638442 9.417742 10.155473	H 7.721279 0.240624 7.747611
H 7.491788 6.216740 5.061280	H 3.192838 6.736091 8.802663	H 6.404069 -0.001185 6.608004

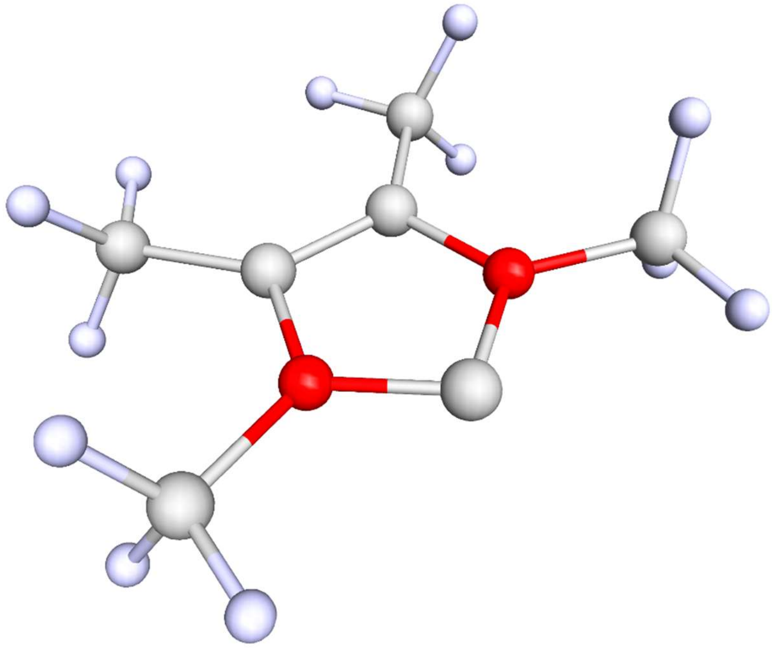

Figure S29: Geometry optimized structure for ^{4Me}NHC: Total energy: −383.13261534142 H, HOMO-LUMO-Gap: 6.799 eV.

Table S6: Atomic coordinates of ^{4Me}NHC

H 9.756516 5.713763 1.946076	C 5.503392 4.173725 3.915348	C 6.489983 7.500742 0.314095
C 7.538808 5.025585 2.850571	C 5.551874 5.886496 2.098352	H 3.809588 6.785718 1.284679
C 9.032237 6.297586 1.382916	C 6.541603 6.482181 1.388102	H 3.672955 6.394558 2.998281
N 6.188802 5.014714 2.970482	H 6.260978 3.610096 4.454763	H 3.575435 5.113420 1.791365
N 7.725749 5.939745 1.867529	H 4.828874 3.477644 3.409808	H 5.456822 7.777119 0.102577
H 9.232741 7.361680 1.533041	H 4.922607 4.767687 4.625938	H 6.931924 7.133644 -0.617258
H 9.136082 6.070183 0.318689	C 4.080977 6.050806 2.042835	H 7.026672 8.413147 0.591600

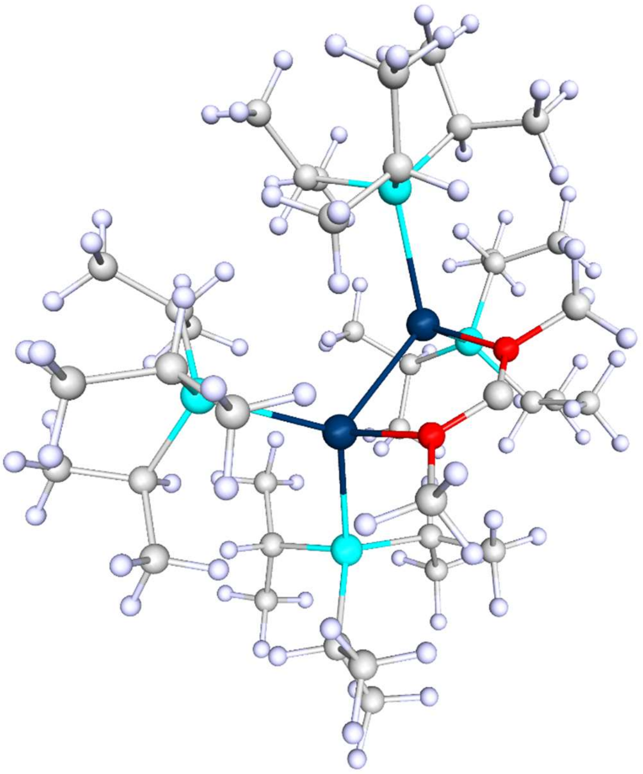

Figure S30: Geometry optimized structure for *N*,*N*-SHC: Total energy: −3234.84576590250 H, HOMO-LUMO-Gap: 3.611 eV.

Table S7: Atomic coordinates of *N*,*N*-SHC

Sn 10.342238 6.677047 9.395019	H 8.069799 9.316030 7.485184	H 5.858873 4.877990 5.701746
Sn 8.217433 5.771598 7.774183	C 9.015180 8.962146 5.611795	H 5.466018 5.061353 3.990717
N 9.292870 5.565867 10.933724	H 8.221261 4.775724 4.266933	H 5.828730 7.251614 2.822447
Si 12.840035 5.820445 9.150008	C 9.484979 3.283086 3.460349	H 6.562659 8.626378 3.643603
Si 10.121462 9.141432 10.364042	C 10.230891 5.441703 4.474916	H 4.897024 8.171557 3.998824
N 7.441241 5.038329 9.659796	C 7.915218 1.007160 5.581374	H 7.678845 11.288236 6.065735
Si 6.693738 7.590504 6.587228	C 6.266127 2.261137 6.952221	H 6.162859 10.636600 6.681804
Si 8.778009 3.654207 6.271716	H 10.800071 3.752296 7.561541	H 6.627714 10.356444 5.004083
C 8.093434 5.044117 10.803102	C 11.142756 1.942082 6.494266	H 8.780700 8.732833 4.568729
C 13.883853 7.014826 10.221312	C 9.699397 2.227734 8.540205	H 9.630400 8.145281 5.997738
C 13.034098 3.987943 9.632667	H 14.342444 5.776429 11.943625	H 9.634491 9.865806 5.614386
C 13.517292 5.993124 7.373968	H 12.777914 6.575084 12.054761	H 9.634742 3.769201 2.489948
C 11.449332 9.596579 11.666759	H 14.256623 7.498427 12.305773	H 10.393804 2.718109 3.681019
C 10.299157 10.286920 8.849287	H 15.867865 7.845645 10.452715	H 11.241393 5.032598 4.503813
C 8.397940 9.171591 11.178708	H 15.442864 7.531679 8.772853	H 10.142171 6.146914 5.304937
C 5.101625 8.087154 7.518594	H 15.872962 6.194546 9.839821	H 10.143798 6.012922 3.544738
C 6.382730 6.826826 4.870368	H 12.731099 4.030363 11.790298	H 5.519534 1.532025 6.618721
C 7.757840 9.160674 6.446794	H 15.032530 4.031500 10.496698	H 11.977126 1.633505 7.135103
C 9.190660 4.332191 4.531308	H 15.036142 3.848324 8.742819	H 11.570841 2.414353 5.608184
C 7.370935 2.397930 5.916731	H 14.594668 2.487104 9.775624	H 9.085605 1.348914 8.328869
C 10.211457 2.878395 7.256023	H 12.114416 4.716575 6.329113	H 10.537237 1.893425 9.160322
H 13.412269 7.992470 10.061551	H 13.570564 3.876328 6.846235	H 13.626592 4.975846 5.468337
C 13.804940 6.698741 11.709659	H 13.512276 8.172551 7.297858	H 8.547169 11.562527 9.108037
C 15.342820 7.148992 9.790028	H 12.065322 7.427073 6.617592	H 10.659642 11.530668 12.315832
C 12.320923 3.543757 10.902616	H 11.821392 11.656644 11.001229	H 5.747923 3.202583 7.130492
H 12.556682 3.460493 8.797421	H 12.374028 11.296706 12.633452	H 6.654841 1.914074 7.911554
C 14.504638 3.572281 9.657017	H 12.086642 9.120392 13.681308	H 11.252283 3.758797 10.863532

H 14.604764 5.990664 7.525116
C 13.189711 4.823612 6.461534
C 13.149725 7.318981 6.721572
H 12.401377 9.259024 11.242339
C 11.576954 11.102687 11.907213
C 11.261702 8.883729 13.001173
C 9.623016 11.651043 8.944698
H 9.798646 9.734240 8.049767
C 11.759521 10.413413 8.426756
C 8.090700 10.418878 12.006003
C 7.272950 8.916520 10.186903
H 8.423428 8.314093 11.863939
C 4.527576 7.029527 8.448995
H 5.435017 8.923785 8.145129
C 4.011151 8.616606 6.587764
H 7.387788 6.502506 4.571757
C 5.500538 5.586771 4.951330
C 5.891079 7.777494 3.781754
C 7.008735 10.422500 6.025688
H 11.223879 7.801724 12.894369
H 10.337586 9.193718 13.495341
H 10.030069 12.258142 9.756178
H 9.766432 12.213525 8.015064
H 11.840571 10.888229 7.443310
H 12.242673 9.437042 8.357546
H 12.341017 11.016423 9.127030
H 8.101305 11.325015 11.395642
H 8.796219 10.563259 12.824502
H 6.314617 8.808443 10.705051
H 7.430851 8.001795 9.610820
H 7.169906 9.747116 9.483327
H 3.640972 7.413012 8.965780
H 5.248536 6.723039 9.206254
H 4.217379 6.136498 7.898883
H 3.601061 7.819548 5.962900
H 4.363305 9.408687 5.925208
H 3.180432 9.022252 7.175350
H 4.471943 5.846081 5.214871
H 12.437646 2.463966 11.046352
H 10.631471 1.032856 6.173736
H 6.915235 2.801210 5.002123
H 13.581556 7.395621 5.717947
H 7.091749 10.337992 12.448422
H 8.667850 2.570437 3.341641
H 9.101368 2.915639 9.142497
H 8.711352 1.021282 4.836691
H 8.307158 0.512985 6.472917
H 7.109935 0.374351 5.192613
C 9.824580 5.605340 12.282344
C 6.183445 4.309110 9.670802
H 9.479332 4.733329 12.839791
H 9.500491 6.500887 12.822466
H 10.916348 5.596009 12.261493
H 6.344459 3.230037 9.736462
H 5.622492 4.514694 8.759858
H 5.576973 4.613179 10.526137

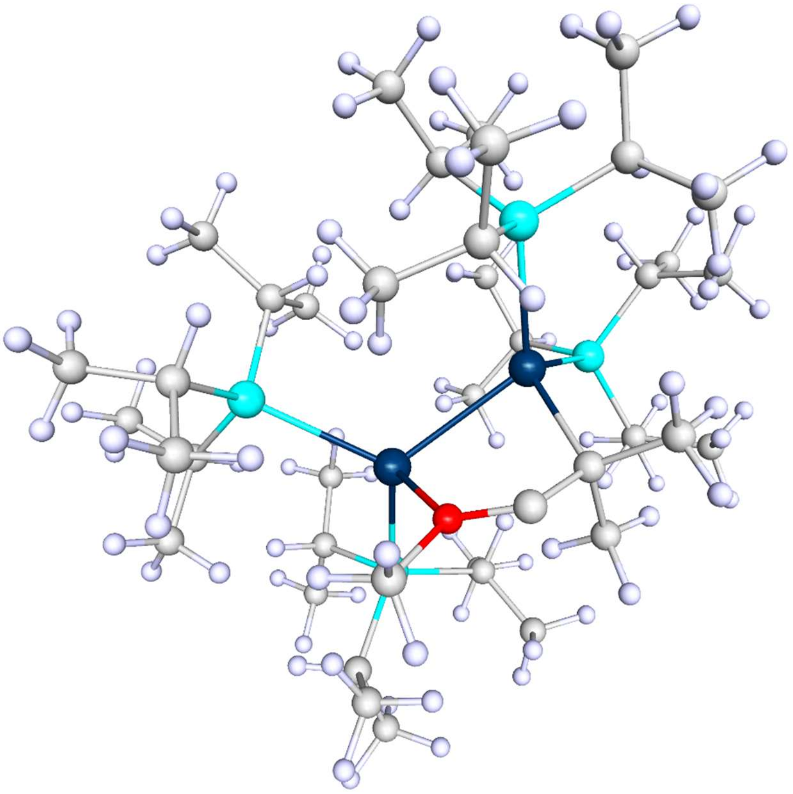

Figure S31: Geometry optimized structure for *N*,*C*-SHC: Total energy: −3258.04962985341 H, HOMO-LUMO-Gap: 3.469 eV.

Table S8: Atomic coordinates of *N,C*-SHC

Sn 10.275912 6.660327 9.594722
Sn 8.238234 5.729392 7.835193
C 9.308512 5.224125 11.061589
Si 12.796542 6.081640 9.083997
Si 9.915233 9.129564 10.512797
N 7.249362 5.274892 9.801571
C 9.407004 8.543443 5.547885
H 7.749753 4.292626 4.436470
C 8.823592 2.653035 3.656862
C 9.763460 4.913062 4.170517
C 7.495102 0.781531 6.297450
C 6.250786 2.387113 7.746277
H 6.272085 6.937621 2.644798
H 7.064889 8.307887 3.417720
H 5.329401 8.068421 3.608415
H 8.403559 11.045968 5.756303
H 6.763903 10.670941 6.276851
H 7.308444 10.205295 4.664011

Si 6.859214 7.556463 6.430175
Si 8.657722 3.433060 6.454014
C 7.843165 5.216615 10.932659
C 13.883824 7.251403 10.128625
C 13.187709 4.231537 9.388267
C 13.194098 6.454421 7.259584
C 11.131577 9.601558 11.914138
C 10.202746 10.335748 9.054485
C 8.122102 9.120193 11.165434
C 5.274368 8.380248 7.125141
C 6.548451 6.674626 4.773428
C 8.131372 8.963166 6.263327
C 8.746750 3.862687 4.589066
C 7.146093 2.254896 6.524896
C 10.268161 2.649287 7.145412
H 13.516210 8.246649 9.844436
C 13.716634 7.123719 11.638071
C 15.361760 7.181907 9.749422
C 13.414721 3.884838 10.857527
H 12.289555 3.697962 9.055293
C 14.363110 3.711159 8.561381
H 14.243520 6.151408 7.142279
C 12.353313 5.620131 6.309852
C 13.085063 7.927990 6.897064
H 12.122675 9.354137 11.515216
C 11.138074 11.097338 12.241187
C 10.939042 8.813387 13.203500
C 9.423579 11.647533 9.104885
H 9.852006 9.783484 8.177922
C 11.688493 10.600710 8.826038
C 7.728422 10.306623 12.042873
C 7.107555 8.939864 10.046329
H 8.075204 8.217110 11.789241
C 4.376204 7.556986 8.034107
H 5.669975 9.208249 7.725255
C 4.430961 8.990645 6.003739
H 7.508517 6.180891 4.592899
C 5.486405 5.588519 4.910236
C 6.288366 7.552473 3.551761
C 7.614005 10.287763 5.710049
H 8.385678 9.139362 7.315161
H 10.757522 3.497877 7.638178
C 11.236224 2.124542 6.087441
C 10.058785 1.557257 8.197194
H 14.224609 6.237810 12.020885
H 12.668901 7.051816 11.933611
H 14.144990 7.989694 12.153529
H 15.935602 7.942960 10.289393
H 15.529139 7.340449 8.681980
H 15.791252 6.211019 10.011368
H 14.395848 4.233666 11.188226
H 15.293774 4.221670 8.822838
H 14.212746 3.832051 7.487954
H 14.516844 2.643330 8.752705
H 11.289553 5.832018 6.448663
H 12.490512 4.549131 6.461013
H 13.745373 8.557647 7.496500
H 12.063321 8.291584 7.033879
H 11.340665 11.725122 11.374603
H 11.913122 11.309454 12.986023
H 11.691434 9.103279 13.944803
H 11.020951 7.738998 13.055452
H 9.958553 9.004802 13.647836
H 9.690792 12.249199 9.976489
H 9.638909 12.251689 8.216304
H 11.849814 11.088319 7.858857
H 12.272629 9.680499 8.826666
H 12.109696 11.254958 9.591126
H 7.815217 11.256129 11.509027
H 8.336897 10.375713 12.944990
H 6.100881 8.800544 10.453040
H 7.331330 8.069083 9.425789
H 7.075098 9.815105 9.392988
H 3.487358 8.134788 8.310649
H 4.882638 7.274731 8.953338
H 4.026132 6.643280 7.545529
H 3.932523 8.214239 5.418775
H 5.007881 9.606712 5.313898
H 3.645248 9.621813 6.432913
H 4.493615 6.019150 5.063791
H 5.686055 4.923337 5.755976
H 5.433260 4.968887 4.008054
H 9.223181 8.304899 4.496658
H 9.863958 7.666409 6.013520
H 10.152143 9.346148 5.572736
H 8.747944 2.984509 2.615270
H 9.769001 2.116689 3.754759
H 10.784637 4.532007 4.222925
H 9.721409 5.806831 4.794446
H 9.589746 5.225241 3.134747
H 5.429541 1.662894 7.706772
H 12.179862 1.823797 6.557087
H 11.471342 2.861830 5.320232
H 9.901108 0.591947 7.714403
H 10.947838 1.460255 8.828849
H 12.597886 5.844203 5.266280
H 8.344640 11.488477 9.135420
H 10.187698 11.423866 12.666549
H 5.809560 3.380182 7.811545
H 6.798521 2.211207 8.676261
H 12.672142 4.327301 11.524018
H 13.392745 2.800288 11.006840
H 10.831896 1.240694 5.587963
H 6.561296 2.590047 5.657224
H 13.344144 8.086887 5.844618
H 6.684882 10.207638 12.361688
H 8.013826 1.943004 3.828772
H 9.209391 1.735242 8.857008
H 8.272959 0.625249 5.547956
H 7.833833 0.317894 7.224327
H 6.606874 0.231098 5.969912
C 9.722572 5.524411 12.492310
C 9.750557 3.809253 10.685394
H 9.341966 4.741807 13.158365
H 10.811439 5.558174 12.597681
H 9.309695 6.470892 12.839902
H 9.186084 3.072463 11.270139
H 10.809318 3.655284 10.887197
H 9.569513 3.606093 9.632389
C 5.810129 5.028811 9.808827
H 5.393773 5.200298 8.816915
H 5.603210 4.000151 10.104903
H 5.325149 5.695024 10.523445

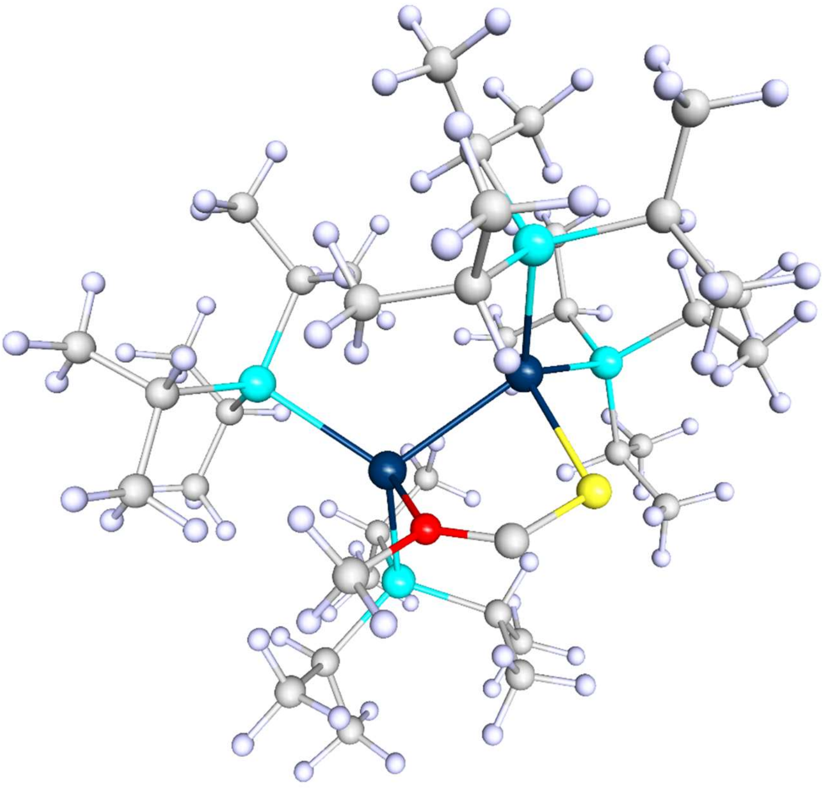

Figure S32: Geometry optimized structure for *N,S*-SHC: Total energy: −3538.35156815834 H, HOMO-LUMO-Gap: 3.681 eV.

Table S9: Atomic coordinates of *N,S*-SHC

Sn 10.339258 6.817525 9.005564	C 7.123572 10.144827 6.168398	H 4.591541 9.369759 5.764028
Sn 8.035697 5.595614 7.986894	H 8.060322 8.857487 7.566939	H 3.147528 8.816305 6.612101
S 9.912913 5.308649 10.987234	C 9.056760 8.605861 5.712858	H 4.716071 5.547617 4.616936
Si 12.799075 6.291372 8.253317	H 8.195886 4.049724 4.850385	H 5.908613 4.564986 5.459588
Si 9.854060 9.074133 10.323546	C 8.966001 2.120403 4.419155	H 5.960780 4.707998 3.699129
N 7.415877 5.333915 10.102277	C 10.287822 4.102518 5.213826	H 6.487992 6.978035 2.629660
Si 6.659544 7.260390 6.473630	C 6.944228 0.639870 6.904598	H 7.012675 8.340277 3.616853
Si 8.343572 3.142402 7.069351	C 5.640292 2.506577 7.944535	H 5.330736 7.823438 3.651837
C 8.229032 5.132005 11.086857	H 10.336022 3.180133 8.413965	H 7.848995 10.957899 6.286239
C 13.875905 7.106060 9.599582	C 10.441705 1.209685 7.607662	H 6.277603 10.367836 6.821327
C 13.010063 4.405427 8.073753	C 9.047229 1.946617 9.563880	H 6.768070 10.178099 5.135218
C 13.327408 7.079522 6.599244	H 14.092296 5.588601 11.137791	H 8.872968 8.598376 4.636145
C 11.201177 9.276582 11.664960	H 12.583475 6.479649 11.255549	H 9.551436 7.665043 5.969183
C 9.908882 10.530369 9.090089	H 14.095326 7.237413 11.753323	H 9.769739 9.413142 5.913526
C 8.142987 8.855167 11.133146	H 15.928909 7.659111 10.024139	H 9.283197 2.362282 3.398764
C 4.906691 7.652896 7.108867	H 15.586609 7.546351 8.297947	H 9.660995 1.370333 4.803797
C 6.669485 6.484692 4.732897	H 15.766090 6.080631 9.263593	H 11.103933 3.463583 5.562130
C 7.773407 8.809004 6.510699	H 13.777334 3.783980 10.007064	H 10.294624 5.001437 5.834821
C 8.952963 3.381442 5.279486	H 15.180010 4.279892 7.915094	H 10.530073 4.405796 4.189929
C 6.714627 2.151669 6.927168	H 14.380178 4.475680 6.356669	H 4.743415 1.896153 7.793025
C 9.639060 2.355932 8.217037	H 14.320895 2.924697 7.192987	H 11.186402 0.849242 8.325656
H 13.539248 8.152698 9.582392	H 11.563578 6.822199 5.367750	H 10.977423 1.509716 6.704889
C 13.642409 6.574413 11.009117	H 12.660444 5.445716 5.305673	H 8.398800 1.072870 9.465429
C 15.367235 7.098443 9.269278	H 13.804551 9.050162 7.431705	H 9.845490 1.682788 10.264300
C 12.898547 3.623745 9.378804	H 12.257696 8.960477 6.584616	H 13.064195 6.938319 4.456157
H 12.162842 4.114587 7.439522	H 11.558003 11.436175 11.455912	H 8.123434 11.632369 9.704538
C 14.290957 4.009822 7.339386	H 12.135377 10.731803 12.962970	H 10.415590 11.016244 12.727431

H 14.381653 6.772654 6.535684
C 12.617021 6.533135 5.370329
C 13.289153 8.600312 6.580631
H 12.142835 9.042199 11.152691
C 11.329046 10.695588 12.222077
C 11.041318 8.289772 12.819657
C 9.187289 11.801474 9.530828
H 9.387456 10.156306 8.202627
C 11.342470 10.837316 8.669344
C 7.828953 9.876163 12.226797
C 7.005961 8.809903 10.121521
H 8.199096 7.867592 11.609349
C 4.103227 6.424755 7.525421
H 5.084045 8.260273 8.006770
C 4.073677 8.483962 6.131212
H 7.709355 6.154783 4.601846
C 5.769669 5.258118 4.626611
C 6.360932 7.465739 3.602307
H 11.918316 8.325688 13.474373
H 10.906366 7.260457 12.488294
H 10.172186 8.539370 13.432968
H 9.610552 12.214450 10.449185
H 9.273150 12.573744 8.758019
H 11.364038 11.481025 7.783601
H 11.900906 9.931688 8.431347
H 11.890649 11.357010 9.457476
H 7.785654 10.895747 11.837268
H 8.561567 9.860659 13.033529
H 6.049308 8.634540 10.625428
H 7.143571 8.004795 9.396978
H 6.911732 9.747459 9.566002
H 3.169716 6.722697 8.014246
H 4.648624 5.786341 8.217941
H 3.833121 5.816217 6.659903
H 3.783835 7.885289 5.264139
H 5.340427 3.553102 7.858361
H 5.982460 2.337246 8.968674
H 12.017611 3.896049 9.962880
H 12.843771 2.549639 9.173579
H 9.806225 0.359055 7.350440
H 6.335874 2.436798 5.936280
H 13.759358 8.986251 5.669876
H 6.852061 9.656921 12.671517
H 7.979717 1.657772 4.351317
H 8.468389 2.745913 10.027938
H 7.693818 0.335693 6.172640
H 7.267536 0.272121 7.880101
H 6.012051 0.120492 6.657326
C 5.994012 5.229023 10.388435
H 5.865107 4.930150 11.429077
H 5.505716 6.194023 10.230856
H 5.518915 4.491511 9.739974

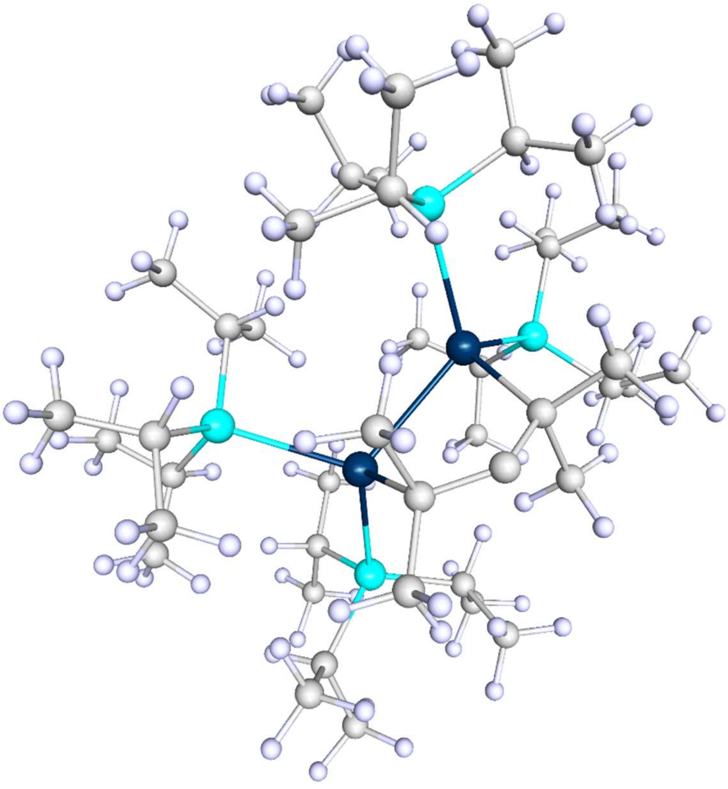

Figure S33: Geometry optimized structure for *C*,*C*-SHC: Total energy: −3281.22141888417 H, HOMO-LUMO-Gap: 3.356 eV.

Table S10: Atomic coordinates of *C,C*-SHC

Sn 10.203943 6.625582 9.645053
Sn 8.142710 5.496128 7.928330
C 9.352272 5.152352 11.191837
Si 12.837452 6.103626 9.338760
Si 9.948904 9.177881 10.503470
C 7.135080 4.924774 9.939357
Si 6.817932 7.333417 6.509493
C 9.364579 3.062131 3.537932
C 10.274245 5.122619 4.613733
C 7.324967 0.942393 5.463257
C 5.831499 2.485385 6.720751
H 10.535212 3.036331 7.778989
C 10.865904 1.517121 6.342385
C 9.281083 1.399363 8.290218
H 7.996028 11.039999 6.692094
H 6.435046 10.399552 7.198499
H 6.885652 10.387138 5.492129
H 8.986249 8.796665 4.790142
H 9.705123 7.816002 6.063522
H 9.886087 9.567717 6.091969
H 9.529723 3.565826 2.578946

Si 8.627243 3.392315 6.341589
C 7.979406 4.772243 11.087036
C 13.872985 7.594316 9.957903
C 13.434707 4.463276 10.137361
C 13.227019 5.905070 7.485939
C 11.312849 9.490955 11.806229
C 10.207639 10.373694 9.035554
C 8.214179 9.428598 11.272247
C 5.071512 7.645259 7.224128
C 6.738479 6.854216 4.664617
C 7.916201 8.879478 6.693028
C 9.152286 4.099842 4.639830
C 7.054580 2.404589 5.822556
C 9.919940 2.307102 7.240827
H 13.245154 8.468588 9.759013
C 14.170798 7.557604 11.454435
C 15.176485 7.805332 9.188357
C 13.362012 4.343517 11.657146
H 12.758020 3.711368 9.711122
C 14.853928 4.106749 9.688658
H 14.324020 5.900110 7.444845
C 12.730038 4.579694 6.935442
C 12.731578 7.064871 6.634704
H 12.214087 9.095356 11.331955
C 11.600799 10.960572 12.112821
C 11.114942 8.709036 13.097581
C 9.804962 11.827844 9.278886
H 9.539514 9.992903 8.260099
C 11.621761 10.291911 8.474223
C 8.111509 10.589458 12.261322
C 7.122910 9.565003 10.215180
H 8.025090 8.507188 11.839021
C 4.277100 6.366404 7.474405
H 5.264529 8.121896 8.195070
C 4.209655 8.605080 6.402771
H 7.762438 6.534619 4.437664
C 5.799904 5.686395 4.384349
C 6.418479 8.013585 3.720704
C 7.264876 10.244407 6.506776
H 8.221864 8.808778 7.741733
C 9.187840 8.755945 5.862588
H 8.227304 4.630274 4.383394
H 14.952100 6.826232 11.672244
H 13.301331 7.297894 12.060687
H 14.537922 8.529750 11.800845
H 15.704104 8.682571 9.578941
H 15.014424 7.970814 8.122602
H 15.851484 6.951958 9.291186
H 14.174777 4.892220 12.133595
H 15.581833 4.816138 10.090861
H 14.971341 4.091140 8.604443
H 15.132315 3.116000 10.064434
H 11.639648 4.546974 6.951784
H 13.096575 3.721286 7.500622
H 13.179857 8.016275 6.927840
H 11.647468 7.175778 6.718942
H 11.940216 11.507114 11.232699
H 12.396313 11.030163 12.863400
H 11.963158 8.863714 13.773207
H 11.029134 7.639492 12.915817
H 10.215244 9.018989 13.634844
H 10.411991 12.307141 10.047812
H 9.934135 12.407998 8.358077
H 11.721414 10.916323 7.579573
H 11.881126 9.271039 8.189661
H 12.369426 10.638609 9.193021
H 8.327475 11.548505 11.785080
H 8.789879 10.480580 13.107394
H 6.129307 9.539073 10.675235
H 7.159017 8.770315 9.470479
H 7.208027 10.512689 9.679535
H 3.374956 6.573530 8.059670
H 4.855036 5.614151 8.010657
H 3.955254 5.914369 6.534171
H 3.899509 8.140400 5.463843
H 4.714130 9.539492 6.159276
H 3.294529 8.854448 6.951003
H 4.756099 6.004229 4.444752
H 5.928719 4.865018 5.091036
H 5.955172 5.287855 3.375722
H 6.477677 7.677549 2.679317
H 7.109692 8.849319 3.832155
H 5.409395 8.398377 3.877909
H 10.240146 2.438037 3.728143
H 11.249822 4.640778 4.678049
H 10.207546 5.834095 5.438718
H 10.257862 5.693500 3.678821
H 4.968468 2.013770 6.237550
H 11.598145 0.970246 6.947201
H 11.422411 2.167665 5.665385
H 8.803360 0.535318 7.827487
H 10.037264 1.017493 8.983926
H 13.046408 4.445881 5.896153
H 8.760748 11.925864 9.577469
H 10.731758 11.484305 12.514898
H 5.557502 3.515995 6.952399
H 5.995572 1.967397 7.666956
H 12.431922 4.723465 12.076632
H 13.459422 3.296749 11.963823
H 10.332844 0.780301 5.737094
H 6.794871 2.913950 4.885789
H 12.960983 6.905047 5.575758
H 7.094059 10.650107 12.663330
H 8.504685 2.403246 3.411234
H 8.523263 1.913344 8.886043
H 8.221025 0.803729 4.857044
H 7.433337 0.327926 6.358113
H 6.480039 0.533543 4.898228
C 9.755288 5.425312 12.630931
C 9.931276 3.780023 10.723088
C 6.104193 3.813164 9.843613
C 6.440091 6.230393 10.424386
H 9.232438 6.300545 13.018617
H 10.827493 5.602051 12.731257
H 9.482616 4.572941 13.261987
H 7.154747 7.029064 10.609974
H 5.883231 6.046701 11.347611
H 5.743867 6.564690 9.657172
H 9.452004 2.944573 11.245581
H 10.989649 3.761529 10.969675
H 9.817078 3.630061 9.651387
H 6.574650 2.864235 9.586068
H 5.619549 3.687762 10.817340
H 5.338804 4.020258 9.09625

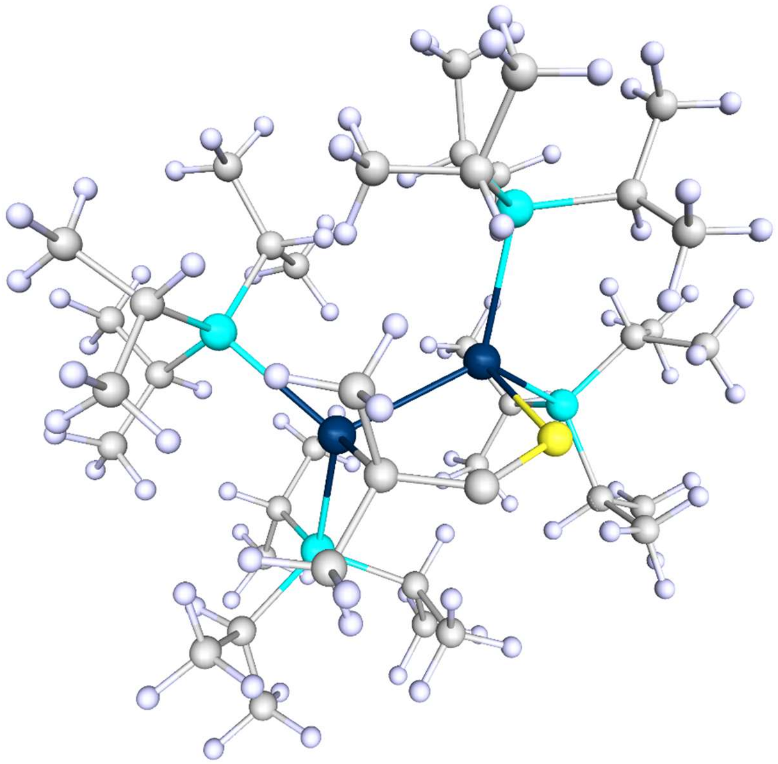

Figure S34: Geometry optimized structure for *C*,*S*-SHC: Total energy: −3561.54282133912 H, HOMO-LUMO-Gap: 3.331eV.

Table S11: Atomic coordinates of *C,S*-SHC

Sn 10.168412 6.683506 9.201371	H 7.963890 8.836591 7.650039	H 5.692065 4.582884 5.496085
Sn 7.855643 5.468445 8.078804	C 8.735075 8.686959 5.693683	H 5.545412 4.882793 3.761075
S 9.531616 5.364203 11.345750	H 7.843199 4.387555 4.569154	H 5.818287 7.250268 2.859226
Si 12.704107 5.923047 8.833288	C 9.141959 2.916255 3.781920	H 6.423418 8.549960 3.882952
Si 9.947501 9.131873 10.248087	C 9.822674 5.150081 4.668873	H 4.776471 7.963865 4.085269
C 7.046744 4.907590 10.203951	C 7.625536 0.663339 6.075792	H 7.536080 10.970432 6.483795
Si 6.470401 7.231796 6.659696	C 5.883829 2.075352 7.160404	H 6.064614 10.315962 7.198653
Si 8.496485 3.366057 6.588970	H 10.588636 3.567739 7.727008	H 6.330591 10.198281 5.458370
C 8.063490 4.725465 11.216883	C 10.911809 1.707079 6.742718	H 8.431975 8.608433 4.647411
C 13.757267 7.216858 9.769502	C 9.610760 2.067956 8.863285	H 9.339758 7.809324 5.928518
C 13.077843 4.136464 9.396153	H 14.472930 6.060104 11.459516	H 9.387980 9.562817 5.776602
C 13.233689 5.993042 7.000587	H 12.856859 6.712653 11.703291	H 9.192861 3.357193 2.780172
C 11.304687 9.303425 11.579645	H 14.263579 7.772465 11.808680	H 10.103660 2.433363 3.968869
C 10.192128 10.390064 8.836473	H 15.689706 8.187709 9.792427	H 10.846402 4.783759 4.603037
C 8.239474 9.314331 11.080917	H 15.130886 7.797188 8.168283	H 9.779696 5.845903 5.509233
C 4.775222 7.511752 7.496120	H 15.756355 6.524303 9.217295	H 9.629875 5.719935 3.753607
C 6.286418 6.597470 4.871553	H 12.985742 4.280561 11.572688	H 5.100599 1.433920 6.741705
C 7.546613 8.814771 6.639703	H 15.140424 4.376289 10.053736	H 11.795560 1.457538 7.341542
C 8.838819 4.000159 4.815857	H 14.995125 4.141600 8.313080	H 11.266225 2.138324 5.805277
C 7.098552 2.087796 6.247013	H 14.770299 2.780566 9.413073	H 8.986031 1.178744 8.747069
C 10.006224 2.660905 7.513802	H 11.894068 4.435425 6.292463	H 10.498537 1.768624 9.429430
H 13.212937 8.158565 9.634337	H 13.518194 3.861783 6.633519	H 13.208110 4.814423 5.183444
C 13.833984 6.924459 11.264617	H 13.037955 8.145212 6.719882	H 8.713753 11.897063 9.402728
C 15.156387 7.437285 9.198568	H 11.606972 7.230730 6.249763	H 10.801273 11.270676 12.405991
C 12.563964 3.690013 10.757517	H 12.010265 11.325176 11.126940	H 5.457075 3.070975 7.296284
H 12.567703 3.522610 8.643869	H 12.446760 10.735673 12.727605	H 6.128139 1.682905 8.148198
C 14.576980 3.850291 9.279290	H 11.866316 8.470743 13.500002	H 11.479563 3.758416 10.830311

H 14.324799 6.098635 7.058669
C 12.947925 4.705211 6.241938
C 12.700325 7.212987 6.262006
H 12.200867 8.886380 11.113980
C 11.651135 10.739231 11.974193
C 11.009813 8.461698 12.817938
C 9.771215 11.825373 9.143634
H 9.536772 10.027323 8.037317
C 11.618162 10.338830 8.300526
C 8.138112 10.481949 12.063268
C 7.091263 9.388436 10.084591
H 8.127564 8.390714 11.664625
C 4.051336 6.203853 7.812786
H 5.027832 7.996789 8.448428
C 3.814946 8.432920 6.743005
H 7.314154 6.340468 4.588877
C 5.434237 5.339051 4.750842
C 5.802252 7.653476 3.877957
C 6.821902 10.140236 6.432702
H 10.793278 7.419419 12.575885
H 10.150776 8.843251 13.374114
H 10.342977 12.256619 9.967684
H 9.935194 12.462228 8.267109
H 11.723650 10.961563 7.405878
H 11.905488 9.321810 8.027623
H 12.342496 10.700713 9.035023
H 8.290758 11.444607 11.570562
H 8.857547 10.409655 12.878083
H 6.126879 9.413594 10.603566
H 7.076415 8.531127 9.411682
H 7.152558 10.289237 9.469194
H 3.223425 6.373078 8.509074
H 4.708817 5.453753 8.255261
H 3.626304 5.763764 6.908931
H 3.455291 7.954686 5.828860
H 4.260145 9.387083 6.463879
H 2.933490 8.645094 7.357885
H 4.374346 5.571478 4.877132
H 12.839843 2.645261 10.939193
H 10.407232 0.767902 6.508897
H 6.746104 2.421805 5.261783
H 13.038101 7.219698 5.220162
H 7.138954 10.503087 12.512034
H 8.378132 2.137752 3.752832
H 9.061347 2.780988 9.481128
H 8.447544 0.597496 5.361640
H 7.979332 0.253283 7.024493
H 6.825701 0.004686 5.719505
C 6.155646 3.675639 10.123202
C 6.218555 6.094659 10.728995
H 6.725395 2.789993 9.846680
H 5.707893 3.489379 11.104756
H 5.351508 3.806097 9.397989
H 6.822518 6.990234 10.840837
H 5.802547 5.843455 11.710120
H 5.391394 6.309020 10.053888

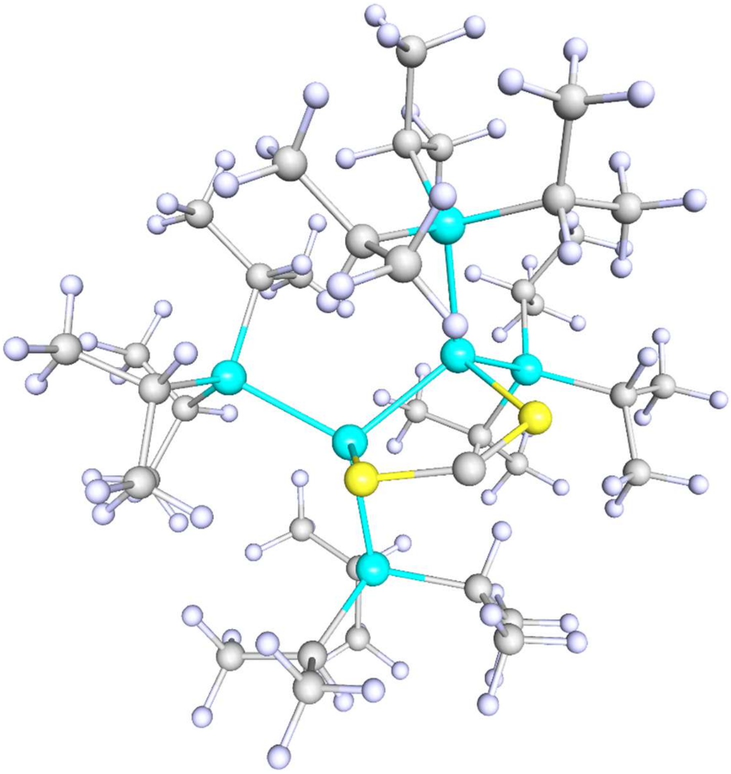

Figure S35: Geometry optimized structure for Si-SHC: Total energy: −3991.92489534577 H, HOMO-LUMO-Gap: 3.867 eV.

Table S12: Atomic coordinates of Si-SHC

Si 0.819271 -0.018146 0.350027
Si -1.255285 -0.776088 -0.656276
S 0.609596 -1.339057 2.174887
Si 2.867925 -0.791584 -0.735045
Si 0.616102 2.039632 1.715675
S -2.203442 -1.220762 1.355103
H -0.508551 2.498377 -3.411755
H 0.371690 1.566531 -2.205931
H -2.817533 4.041132 -0.685537
H -2.530844 4.134893 -2.422133
H -1.301229 4.692384 -1.296701
H -1.165933 0.445846 3.692881
H 4.145997 -2.227618 -3.258898
H 2.370927 -0.154593 -3.670349
H 1.314264 -1.473899 -4.155029
H 0.794462 -0.341258 -2.911837
H 5.869812 -2.686328 0.761065
H 5.362625 -2.667666 -0.922886

Si -2.556250 1.029800 -1.647019
Si -1.369813 -3.108424 -1.404614
C -1.007690 -1.595546 2.437491
C -4.144968 1.208489 -0.603432
C -2.939134 0.831014 -3.506522
C -1.415908 2.537631 -1.422036
C -5.108039 2.290448 -1.094726
H -3.769878 1.508736 0.385218
C -4.926814 -0.090734 -0.426154
H -2.012168 0.455303 -3.953221
C -3.266950 2.150327 -4.207977
C -4.043631 -0.177129 -3.795417
H -1.035129 2.392593 -0.405742
C -0.215029 2.474928 -2.359982
C -2.061585 3.919425 -1.461029
C -1.241429 2.104015 2.246670
C 1.638630 1.711252 3.295126
C 1.050098 3.686462 0.834106
C 3.078572 1.268887 3.091170
C 1.589279 2.854890 4.309643
H 1.102372 0.863698 3.736181
C 1.129654 4.897000 1.766250
H 0.190047 3.848152 0.176724
C 2.288608 3.674777 -0.046460
H -1.748269 1.489940 1.493795
C -1.539801 1.465009 3.601844
C -1.894398 3.484540 2.205402
H -5.433420 -0.372828 -1.351150
H -4.299336 -0.929304 -0.126389
H -5.697178 0.027171 0.342400
H -4.636809 3.261610 -1.238601
H -5.923548 2.421398 -0.375548
H -5.565923 2.001499 -2.044016
H -2.445498 2.865238 -4.170010
H -3.485245 1.961251 -5.264707
H -4.147712 2.632909 -3.778995
H 0.451685 3.325600 -2.191189
H -1.117921 2.051245 4.420954
H -2.622523 1.422969 3.759169
H 0.598952 3.301867 4.410078
H 1.886274 2.490333 5.298546
H 2.281851 3.653625 4.039940
H 1.117018 5.819218 1.175236
H 0.310967 4.954634 2.481636
H 2.063912 4.893001 2.330149
H 2.349522 4.598408 -0.632910
H 2.295705 2.838396 -0.743205
H 3.197780 3.618779 0.554209
C 2.263564 -1.797226 -2.231407
C 3.893899 -1.859508 0.479176
C 3.919912 0.679131 -1.348662
C -0.063277 -4.036100 -0.331238
C -3.080647 -3.952492 -1.194957
C -0.866280 -3.360811 -3.241627
H 0.226504 -3.293674 -3.210603
C -1.212095 -4.751769 -3.777030
C -1.339624 -2.307361 -4.231807
C 0.731912 -5.108312 -1.074584
C -0.568577 -4.649503 0.971334
H 0.642699 -3.246483 -0.053955
H -2.981578 3.383012 2.293645
H -1.689610 4.037304 1.288725
H -1.568082 4.102660 3.045491
H 3.949499 -1.267471 1.401500
C 3.261848 -3.202214 0.831483
C 5.330870 -2.099847 0.009437
C 1.656611 -0.890314 -3.295962
C 3.281098 -2.752636 -2.849615
H 1.453545 -2.410569 -1.823919
C 4.898370 0.358559 -2.479353
C 4.659952 1.376142 -0.213182
H 3.186808 1.385832 -1.756958
H 3.648076 -3.487182 -2.132250
H 2.819826 -3.306150 -3.675924
H 5.890424 -1.178708 -0.145752
H 5.644669 -0.379202 -2.179451
H 4.403471 -0.019376 -3.373714
H 5.439829 1.266592 -2.766355
H 4.009454 1.594813 0.630198
H 5.093873 2.321293 -0.554497
H 5.480176 0.761893 0.163056
H 3.531248 0.977015 4.044757
H 3.155141 0.411255 2.419982
H 3.691584 2.074291 2.678062
H -1.107307 -1.298625 -3.895375
H -2.415455 -2.360102 -4.400748
H -0.856317 -2.451291 -5.204537
H -0.757964 -4.895424 -4.763522
H -0.858236 -5.557907 -3.135589
H -2.289457 -4.878299 -3.900972
H -4.106314 -0.385704 -4.868519
H -3.891380 -1.124135 -3.282123
H -5.016777 0.212486 -3.486978
C -3.758161 -3.999637 0.171296
C -4.130742 -3.551599 -2.225626
H -2.765912 -4.981940 -1.434106
H -3.091498 -4.272924 0.984314
H -4.199253 -3.035539 0.426388
H -4.575324 -4.729296 0.145788
H -3.749699 -3.502515 -3.244997
H -4.556764 -2.577839 -1.977729
H -4.958570 -4.268714 -2.218173
H 3.167644 -3.841789 -0.048652
H 3.892029 -3.735410 1.550866
H 2.274223 -3.101100 1.279342
H -1.074860 -3.935240 1.618839
H -1.250914 -5.481049 0.776181
H 0.277770 -5.054533 1.536578
H 0.107830 -5.974982 -1.306474
H 1.543126 -5.470086 -0.434664
H 1.180019 -4.758732 -2.005740

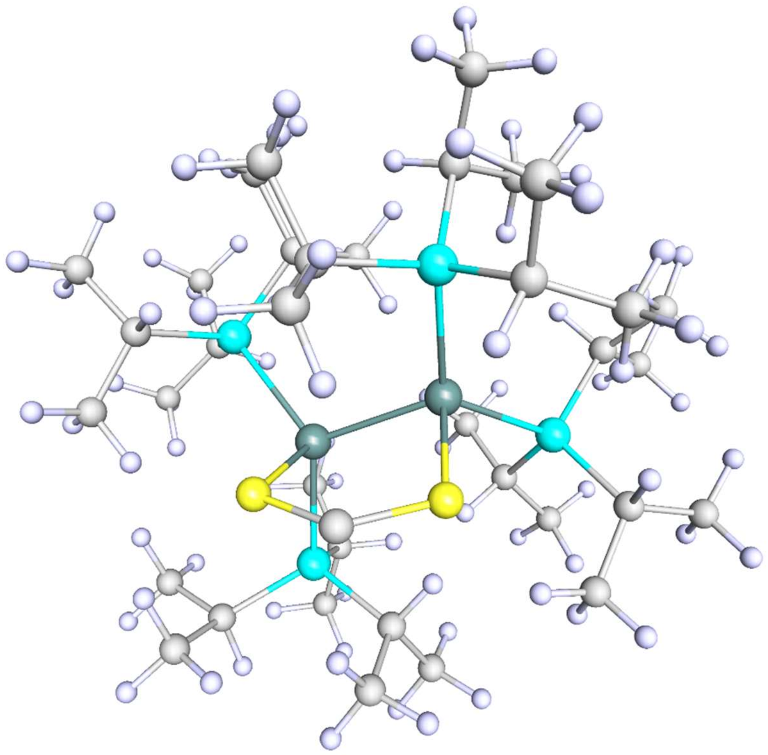

Figure S36: Geometry optimized structure for Ge-SHC: Total energy: −7566.69217932734 H, HOMO-LUMO-Gap: 3.841 eV.

Table S13: Atomic coordinates of Ge-SHC

Ge 0.704733 -0.064364 0.320524	H -0.881046 2.450192 -3.527090	H 4.122784 -2.380605 -3.187872
Ge -1.448141 -0.876888 -0.697272	H 0.040348 1.466932 -2.393654	H 2.429032 -0.302847 -3.705777
S 0.449950 -1.388501 2.269639	H -3.063246 3.948343 -0.701240	H 1.294796 -1.566761 -4.162852
Si 2.800124 -0.893841 -0.721890	H -2.849312 4.040532 -2.448237	H 0.841023 -0.373526 -2.949405
Si 0.459387 2.033538 1.666289	H -1.577921 4.609470 -1.375934	H 5.711612 -2.809481 0.914562
S -2.375010 -1.361359 1.436324	H -1.315339 0.458242 3.655314	H 5.273484 -2.797563 -0.789160
Si -2.825112 0.940297 -1.661265	H -1.285580 2.072137 4.364976	H 5.791095 -1.309298 -0.002039
Si -1.497146 -3.240741 -1.448229	H -2.780858 1.423274 3.702031	H 5.637488 -0.521934 -2.040696
C -1.156315 -1.663912 2.494499	H 0.402848 3.370994 4.315724	H 4.451409 -0.186933 -3.296624
C -4.371181 1.093273 -0.555468	H 1.699721 2.613170 5.236657	H 5.466201 1.109740 -2.675810
C -3.267807 0.716803 -3.504408	H 2.081007 3.741979 3.942132	H 3.874838 1.542035 0.634492
C -1.683074 2.453280 -1.493854	H 1.034019 5.774886 0.944280	H 5.045337 2.205219 -0.505754
C -5.368137 2.161515 -1.007237	H 0.231339 4.993538 2.305180	H 5.352540 0.663947 0.286478
H -3.962446 1.396519 0.418790	H 1.977688 4.862629 2.113981	H 3.367163 1.076584 4.051483
C -5.121628 -0.221073 -0.352666	H 2.122026 4.434840 -0.879967	H 3.013281 0.449132 2.444256
H -2.346245 0.362975 -3.979182	H 2.017669 2.673093 -0.894105	H 3.532314 2.125950 2.648308
C -3.654852 2.025111 -4.196870	H 3.008955 3.493899 0.310680	H -1.179430 -1.311160 -3.828763
C -4.352395 -0.321963 -3.755901	C 2.218554 -1.897373 -2.227313	H -2.519736 -2.320038 -4.361215
H -1.248668 2.313574 -0.498293	C 3.761718 -1.955009 0.544811	H -0.977220 -2.399157 -5.201328
C -0.532890 2.391448 -2.493643	C 3.881815 0.562886 -1.313194	H -0.836030 -4.859007 -4.883754
C -2.338817 3.830721 -1.506950	C -0.168047 -4.137721 -0.387461	H -0.931174 -5.602315 -3.290077
C -1.396017 2.098476 2.190145	C -3.185861 -4.123920 -1.259839	H -2.369826 -4.905262 -4.025782
C 1.476397 1.764853 3.257746	C -0.967018 -3.405861 -3.284282	H -4.448305 -0.528672 -4.827038
C 0.880211 3.632324 0.698875	H 0.124960 -3.331264 -3.235832	H -4.152016 -1.265308 -3.252833
C 2.922245 1.329880 3.083353	C -1.294554 -4.770202 -3.892879	H -5.325269 0.036149 -3.410424
C 1.402027 2.939592 4.234730	C -1.439308 -2.297264 -4.213127	C -3.850170 -4.187542 0.112009
H 0.942917 0.924995 3.717648	C 0.613220 -5.213870 -1.139407	C -4.246048 -3.713723 -2.276054

C 1.029676 4.877823 1.572960
H -0.009137 3.782545 0.077859
C 2.070258 3.545025 -0.242770
H -1.905454 1.481002 1.440712
C -1.697729 1.472258 3.550685
C -2.032472 3.486479 2.141195
H -5.652813 -0.514526 -1.260229
H -4.467371 -1.046636 -0.073600
H -5.867744 -0.117540 0.441299
H -4.915565 3.139480 -1.166413
H -6.157654 2.279969 -0.257600
H -5.857233 1.867751 -1.939417
H -2.853618 2.763401 -4.186021
H -3.901014 1.827656 -5.245925
H -4.535011 2.483397 -3.740826
H 0.159726 3.223730 -2.337778
C -0.640896 -4.733299 0.935118
H 0.537266 -3.337667 -0.137945
H -3.118710 3.402737 2.255604
H -1.840547 4.021742 1.211570
H -1.677107 4.112902 2.963546
H 3.786546 -1.347162 1.458687
C 3.090711 -3.279923 0.891381
C 5.212473 -2.221625 0.136992
C 1.669096 -0.983237 -3.316787
C 3.232812 -2.882180 -2.803338
H 1.380050 -2.485926 -1.841518
C 4.908522 0.210959 -2.390820
C 4.570331 1.281572 -0.159704
H 3.172107 1.263818 -1.768900
H 3.558915 -3.617790 -2.067312
H 2.787064 -3.433077 -3.639724
H -2.863241 -5.146978 -1.515244
H -3.178920 -4.487864 0.911762
H -4.270089 -3.220067 0.390344
H -4.680488 -4.901921 0.082786
H -3.868247 -3.618823 -3.293747
H -4.692845 -2.759224 -1.991524
H -5.057892 -4.448562 -2.293001
H 3.025788 -3.934515 0.019414
H 3.676152 -3.810089 1.649648
H 2.085345 -3.151199 1.290502
H -1.166579 -4.019528 1.567014
H -1.296470 -5.591746 0.766377
H 0.222657 -5.095897 1.502848
H -0.020822 -6.073651 -1.371921
H 1.426882 -5.586038 -0.508328
H 1.056449 -4.862127 -2.072112

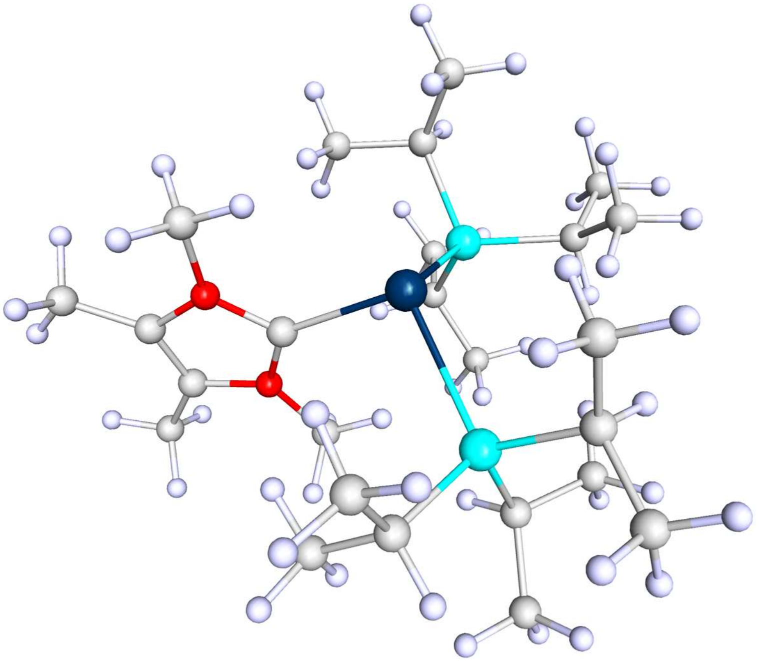

Figure S37: Geometry optimized structure for NHC-SnTIPS$_2$: Total energy: −1886.96443281319 H, HOMO-LUMO-Gap: 4.043 eV.

Table S14: Atomic coordinates of NHC-SnTIPS$_2$

Sn 9.278908 4.061642 3.945738
Si 8.558629 1.573358 3.412325
Si 8.693845 4.676755 6.456792
H 9.765982 5.690260 1.763723
C 7.524639 5.010270 2.804474
C 9.362438 0.376160 4.671677
C 6.687389 1.142672 3.274374
C 9.283344 1.258468 1.668326
C 9.528888 7.403140 6.021655
H 10.222216 3.181524 7.614857
C 10.338698 4.943504 8.805076
C 11.606513 4.501747 6.695775
H 9.101539 7.193036 1.072463
H 9.073249 5.687505 0.122833
C 5.511513 4.312356 4.046802
C 5.533244 5.700099 1.982565
H 8.070049 1.837303 6.973130
H 6.739875 1.833247 8.136440
H 5.803674 4.044076 8.995106
H 6.333498 5.581772 8.311232
H 7.396767 4.679479 9.382738
H 6.863138 8.032139 6.023754
H 6.216221 6.403984 6.236257
H 7.145983 6.805916 4.788481

C 7.240497 3.814547 7.385019
C 8.374803 6.563691 6.556874
C 10.295073 4.262503 7.438408
C 8.986132 6.109316 1.125693
N 6.186270 4.974818 2.961447
N 7.706086 5.773200 1.704853
H 8.640474 0.328086 5.496818
C 9.538435 -1.043140 4.136971
C 10.670092 0.903060 5.252470
H 6.237169 2.034317 2.819760
C 6.048900 0.932651 4.642456
C 6.358026 -0.035855 2.361005
H 9.005598 0.228829 1.406354
C 10.801733 1.370803 1.603394
C 8.649160 2.197728 0.647166
H 6.434598 3.735030 6.646227
C 7.616707 2.395096 7.796337
C 6.666242 4.576508 8.577314
H 8.266722 6.780254 7.628449
C 7.076635 6.968416 5.867921
C 6.502270 6.205982 1.178237
H 10.224102 -1.070452 3.285921
H 8.594123 -1.481777 3.808239
H 9.954992 -1.700960 4.908535
H 11.070420 0.214952 6.005841
H 10.533364 1.873050 5.735947
H 11.437410 1.028766 4.484714
H 4.956004 0.882311 4.572243
H 6.305100 1.722519 5.352152
H 6.383316 -0.009791 5.085165
H 6.786772 -0.968303 2.736412
H 6.728178 0.107756 1.344393
H 5.273468 -0.185912 2.298584
H 11.161557 1.220741 0.578657
H 11.298343 0.632121 2.235575
H 11.134109 2.360978 1.930531
H 8.936022 3.232222 0.859414
H 7.556603 2.152724 0.652392
H 8.987924 1.967250 -0.369471
H 8.335425 2.403475 8.620287
H 9.756421 7.145848 4.982309
H 10.444292 7.258612 6.597587
H 9.283674 8.471316 6.055335
H 11.236195 4.643827 9.358407
H 9.475287 4.694001 9.424110
H 10.369558 6.032723 8.709057
H 11.761040 5.559343 6.470696
H 11.651815 3.969374 5.743767
H 12.454878 4.170720 7.305941
H 6.262181 3.761564 4.607616
H 4.761053 3.618793 3.666988
H 5.033887 5.042318 4.701973
C 4.059648 5.824527 1.930183
C 6.415707 7.050156 -0.034041
H 3.763489 6.450938 1.089657
H 3.661339 6.278003 2.841916
H 3.576284 4.851237 1.807996
H 5.373669 7.260195 -0.271894
H 6.863727 6.557527 -0.901335
H 6.926695 8.007350 0.101255

7.2 Selected frontier orbitals of **10**

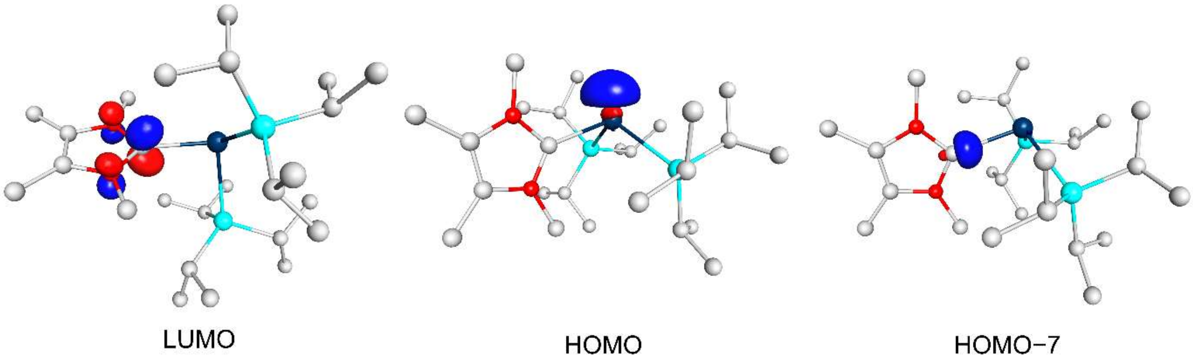

Figure S38: Visualization of a carbene stabilized stannylene **10** by DFT calculation.

7.3 Selected frontier orbitals of **8**

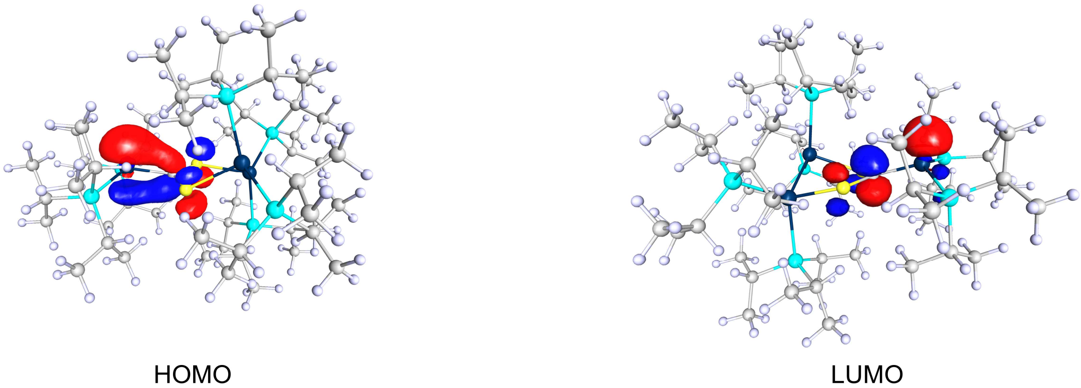

Figure S39: Visualization of a stannaethene **8** by DFT calculation.

7.4 Calculated UV/Vis spectrum of **8**

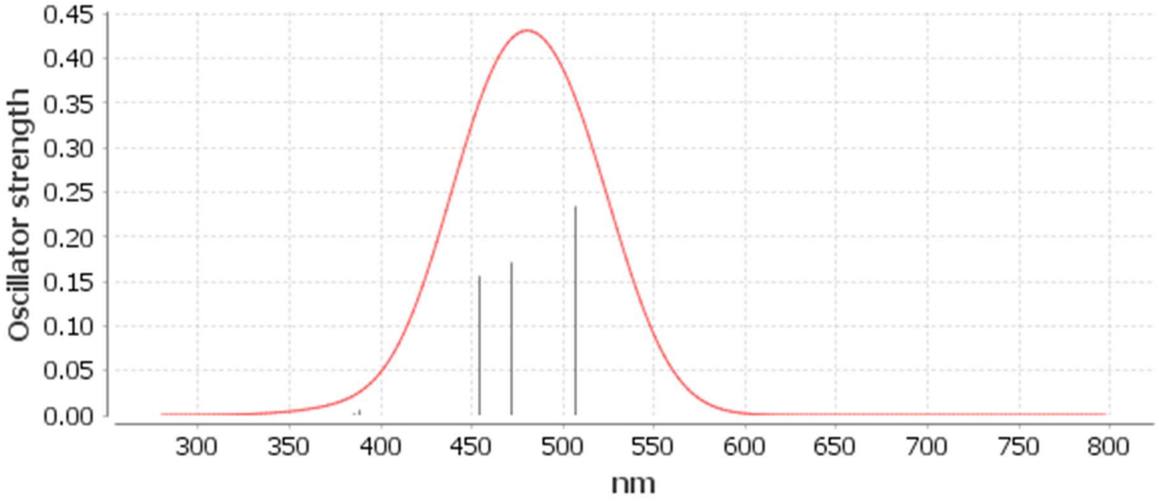

Figure S40: Calculated UV/Vis spectrum of **8**.

8 References

(1) Edgar, M.; Zeinali, F.; Mojally, M.; Hughes, C.; Riaz, S.; Weaver, G. W. NMR spectral analysis of second-order ^{19}F-^{19}F, ^{19}F-^{1}H and ^{13}C-^{19}F coupling constants in pentafluorobenzene and tetrafluoro-4-(morpholino)pyridine using ANATOLIA. *J. Fluorine Chem.* **2019**, *224*, 35-44.

(2) McConnell, H. M.; McLean, A. D.; Reilly, C. A. Analysis of Spin-Spin Multiplets in Nuclear Magnetic Resonance Spectra. *J. Chem. Phys.* **1955**, *23* (6), 1152-1159.

(3) Spek, A. L. Single-crystal structure validation with the program PLATON. *J. Appl. Crystallogr.* **2003**, *36* (1), 7-13.

(4) Van der Sluis, P.; Spek, A. L. BYPASS: an effective method for the refinement of crystal structures containing disordered solvent regions. *Acta Crystallogr. Sect. A: Found. Crystallogr.* **1990**, *46* (3), 194-201.
